# Design, Synthesis, Biological Evaluation and Molecular Docking Studies of New *N*-Heterocyclic Compounds as Aromatase Inhibitors

**DOI:** 10.3390/ph19020224

**Published:** 2026-01-27

**Authors:** Fatih Tok, Begüm Nurpelin Sağlık Özkan, Yusuf Özkay, Zafer Asım Kaplancıklı

**Affiliations:** 1 Department of Pharmaceutical Chemistry, Faculty of Pharmacy, Marmara University, 34854 İstanbul, Türkiye; fatih.tok@marmara.edu.tr; 2 Department of Pharmaceutical Chemistry, Faculty of Pharmacy, Anadolu University, 26470 Eskişehir, Türkiye; 3 Central Research Laboratory (MERLAB), Faculty of Pharmacy, Anadolu University, 26470 Eskişehir, Türkiye

**Keywords:** aromatase, thiadiazole, triazole, antiproliferative

## Abstract

**Background/Objectives:** Breast cancer is the most common cancer and the second leading cause of cancer death in women. The aromatase enzyme plays a role in estrogen biosynthesis and is an important biological target for breast cancer treatment. For this purpose, some new 1,3,4-thiadiazole (**4a**–**4j**) and 1,2,4-triazole (**5a**–**5j**) structures were designed and synthesized based on the structures of the existing aromatase inhibitors letrozole and anastrozole. **Methods:** The antiproliferative activities of the compounds were tested against MCF-7 cancer cells. The NIH3T3 healthy cells were used to evaluate the selectivity of the compounds. The inhibitory activities of all compounds were tested against the aromatase enzyme. **Results:** The 1,2,4-triazole derivatives **5b**, **5c**, **5e**, **5f** and **5g** exhibited the highest antiproliferative activity against MCF7 cells with IC_50_ values ranging from 3.142 to 10.415 μM. Similar to the antiproliferative activity results, triazole derivatives **5b**, **5c**, **5e**, **5f** and **5g** exhibited comparable anti-aromatase activity to letrozole (IC_50_ = 0.031 μM) with IC_50_ values ranging from 0.064 to 2.224 μM and demonstrated the highest anti-aromatase activity within the series. The interactions of compound **5c**, the most potent compound based on activity results, with the aromatase enzyme have been elucidated through molecular docking and MD simulation studies. **Conclusions:** According to experimental studies and molecular docking findings, compound **5c** shows promise for further studies with its aromatase enzyme inhibitory potential.

## 1. Introduction

Breast cancer is the most commonly diagnosed cancer in women in the world [1]. Approximately one in eight women will be diagnosed with invasive breast cancer in their lifetime [2]. According to 2024 data, 310,720 women in the United States were diagnosed with invasive breast cancer, while 42,250 women died from breast cancer. According to studies conducted between 2012 and 2021, the incidence of breast cancer increased by 1% each year [3]. Treatment options for breast cancer include surgical removal of part or all of the cancerous tissue, radiation or hormonal therapy and chemotherapy [4]. As in all types of cancer, the need for highly effective and selective chemotherapeutic agents is increasing day by day in the fight against breast cancer. Approximately 80% of breast cancer cases are estrogen-dependent, meaning that cancerous tissue has the chance to develop in the presence of estrogen [5]. Therefore, molecules that stop estrogen production or block estrogen receptors are needed in the cancer treatment [6].

**Figure 1:**
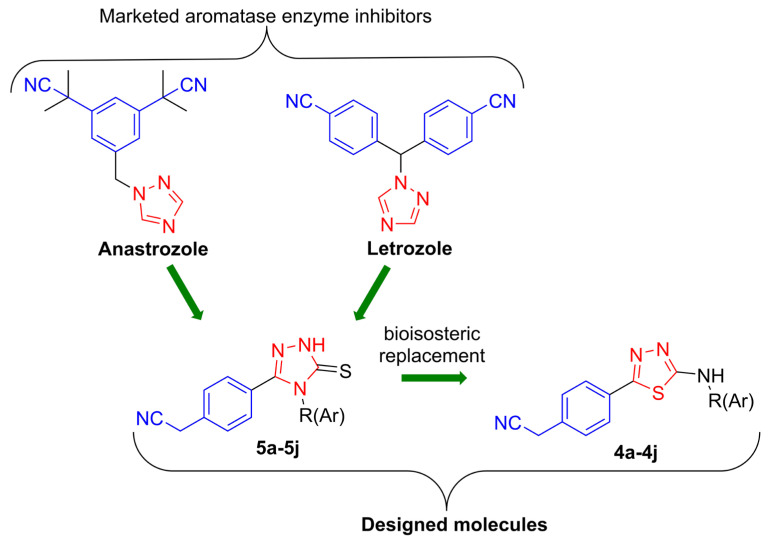
The design strategies of target compounds.

Aromatase is an enzyme of the cytochrome P450 superfamily that can catalyze the aromatization of androgens to estrogen in humans [7]. Aromatases are commonly found in the ovary, placenta, brain and adipose tissue. The ovaries in premenopausal women and adipose tissue in postmenopausal women are the main sources of aromatase production [8]. Aromatase inhibitors are used in the treatment of breast cancer by suppressing estrogen production [9]. There are three generations of aromatase inhibitors developed to date: the first-generation aromatase inhibitor, aminoglutethimide; second-generation aromatase inhibitors, adrozole and formestane; and third-generation aromatase inhibitors, anastrozole, letrozole and exemestane. The activity, selectivity and reversible binding of molecules against aromatase enzyme increase from first-generation to third-generation inhibitors [10]. According to their chemical structure and mechanism of action, third-generation aromatase inhibitors are divided into two classes: steroidal and non-steroidal [11]. Exemestane is a steroidal and irreversible inhibitor that covalently binds to the enzyme, while anastrozole and letrozole are non-steroidal and reversible inhibitors without covalent interaction [12]. If we divide the chemical structures of the anastrozole and letrozole molecules into two parts, one of these parts carries a 1,2,4-triazole ring in common. The nitrogen atoms in the triazole ring interact with the heme iron of the aromatase enzyme and stop the enzyme from working [13,14]. The other part consists of 2-phenylacetonitrile or benzonitrile moieties containing a cyano group in both inhibitor molecules. Based on this information, potent aromatase inhibitors have been designed as new drug candidates. As in aromatase inhibitors, the target compounds have 2-phenylacetonitrile moieties in their general structure. Half of the target compounds have a 1,2,4-triazole ring in their structure, as in aromatase inhibitors, while the other half of the target compounds have 1,3,4-thiadiazole rings, which are the bioisosters of 1,2,4-triazole structure (Figure 1). Thus, the activities of 1,2,4-triazole ring and 1,3,4-thiadiazole ring can be compared in aromatase enzyme studies.

In accordance with the strategy outlined above, this study aimed to synthesize new 1,2,4-triazole and 1,3,4-thiadiazole structures with high aromatase enzyme inhibitory activity and evaluate their biological activity. First, the cytotoxic activities of the synthesized compounds were examined on MCF-7 and NIH3T3 cell lines. Then, the activity of all compounds against the aromatase enzyme was tested. In silico molecular docking and ADME studies were then conducted to determine the potential of these compounds as drug candidates.

## 2. Results and Discussion

### 2.1. Chemistry

**Scheme 1:**
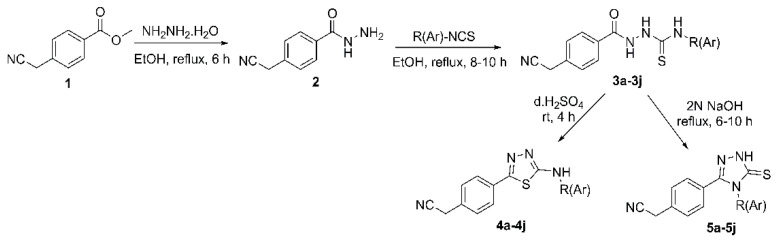
The synthesis pathways of target compounds, 1,3,4-thiadiazole (**4a**–**4j**) and 1,2,4-triazole (**5a**–**5j**). R(Ar): Phenyl (**a**), 4-fluorophenyl (**b**), 4-chlorophenyl (**c**), 4-bromophenyl (**d**), 4-methylphenyl (**e**), 4-methoxyphenyl (**f**), 4-nitrophenyl (**g**), methyl (**h**), ethyl (**i**), and cyclohexyl (**j**).

The synthesis of the target compounds, 2,5-disubstituted-1,3,4-thiadiazol-5-amine (**4a**–**4j**) and 1,2-disubstituted-5-thioxo-4,5-dihydro-1*H*-1,2,4-triazol-3-yl (**5a**–**5j**), is completed at four different steps (Scheme 1). Firstly, 4-(cyanomethyl)benzohydrazide (**2**) is synthesized by the nucleophilic substitution reaction of methyl 4-(cyanomethyl)benzoate (**1**) with hydrazine monohydrate in ethanol. In the second step, new thiosemicarbazide derivatives (**3a**–**3j**) are formed by nucleophilic addition and the reaction of hydrazide compounds (**2**) with various isothiocyanate derivatives in ethanol. In the third step, the thiosemicarbazide derivatives (**3a**–**3j**) are directly stirred with concentrated sulfuric acid and 1,3,4-thiadiazole derivatives (**4a**–**4j**) are obtained by ring closure reaction. In the fourth step, the thiosemicarbazide derivatives (**3a**–**3j**) are heated in a dilute alkaline medium and 1,2,4-triazole derivatives (**5a**–**5j**) are gained as a result of ring closure reaction. The target compounds, 1,3,4-thiadiazole (**4a**–**4j**) and 1,2,4-triazole (**5a**–**5j**) derivatives, were prepared in high yields (75–85%) and in pure form. Different techniques such as IR, ^1^H-NMR, ^13^C-NMR spectroscopies and elemental analysis were used for the structural characterization of the compounds.

The N-H, C=O and C=S bonds in the structure of thiosemicarbazide derivatives (**3a**–**3j**) gave specific IR bands in the range of 3130–3342 cm^−1^, 1662–1678 cm^−1^ and 1207–1238 cm^−1^, respectively. The characteristic C=O bands of thiosemicarbazide have been replaced by C=N bands of the 1,3,4-thiadiazole (**4a**–**4j**) and 1,2,4-triazole structures (**5a**–**5j**) in the range of 1599–1714 cm^−1^. In addition, N-H bonds of 1,3,4-thiadiazole and 1,2,4-triazole derivatives were detected with distinct bands in the range of 3146–3402 cm^−1^.

When the ^1^H-NMR spectra of thiosemicarbazides (**3a**–**3j**) and the hydrazide compound (**2**) are compared, the most important difference is that the peak (NH_2_) detected with the 2H integral at 4.50 ppm of the hydrazide is not seen in the spectra of thiosemicarbazides. In addition, three NH protons forming the thiosemicarbazide structures were recorded as singlet peaks from 7.74 ppm to 10.70 ppm. These three NH protons were replaced by a single NH proton in the range of 7.83–11.62 ppm in the ^1^H-NMR spectra of thiadiazole structures (**4a**–**4j**). Similarly, NH protons in triazole structures (**5a**–**5j**) were found as singlet peaks between 12.42 ppm and 14.22 ppm.

In the ^13^C-NMR spectra of thiosemicarbazides, thion (C=S) and carbonyl (C=O) carbons were detected in the ranges of 180.64–184.84 ppm and 165.45–166.03 ppm, respectively. The peaks of thiadiazole derivatives (**4a**–**4j**) in the range of 155.85–172.32 ppm were attributed to (C=N) carbons forming the heterocyclic structure. Similarly, the carbons forming the triazole ring (**5a**–**5j**) showed specific peaks in the range of 150.30–172.74 ppm.

In a previous study, in the IR spectrum of 2,5-disubstituted-1,3,4-thiadiazole structures, N-H and C=N stretching bands were detected in the range of 3134–3481 cm^−1^ and 1593–1633 cm^−1^, respectively. The NH proton attached to the 1,3,4-thiadiazole ring was recorded in the range of 10.35–11.28 ppm in the ^1^H-NMR spectrum. The imine (C=N) carbons of the thiadiazole structure are visualized in the downfield region (156.08–165.97 ppm) in the ^13^C-NMR spectrum [15].

In another study synthesized based on thiosemicarbazides, the C=S absorption bands of 1,2,4-triazole-3-thione derivatives were shown at 1323–1326 cm^−1^. The NH protons were observed in the range of 14.15–14.31 ppm in ^1^H-NMR spectra. The C=S carbon attached to the 1,2,4-triazole ring was reported in the range of 168.97–169.62 ppm in ^13^C-NMR spectrums [16]. The spectral data in these two recent studies were found to be compatible with the data in this study.

### 2.2. Biological Activity

The antiproliferative activity of 1,3,4-thiadiazole (**4a**–**4j**) and 1,2,4-triazole (**5a**–**5j**) derivatives was evaluated against human breast cancer cells (MCF-7) and mouse embryonic fibroblast cells (NIH3T3). The NIH3T3 cells were used to determine the selectivity of all compounds. The compounds with a high selectivity index and cytotoxic effect against MCF-7 were identified as **4a**, **4b**, **4c**, **4e**, **4f**, and **4g** among thiadiazole derivatives and **5a**, **5b**, **5c**, **5d**, **5e**, **5f**, and **5g** among triazole derivatives. All compounds were also tested on the aromatase enzyme. Among the compounds, **5b**, **5c**, **5e**, **5f** and **5g** exhibited significant enzyme inhibitory activity. Doxorubicin and letrozole were used as negative controls in this study (Table 1).

#### Structure–Activity Relationship Studies

Aromatic (substituted phenyl ring), cycloaliphatic (cyclohexane) and alkyl (methyl, ethyl) groups were preferred as R(Ar) groups in the chemical structures of the compounds. Some interesting structure–activity relationships were obtained based on the cytotoxic and aromatase inhibitory activity results.

✓ Based on both cytotoxic and aromatase activity results, compounds bearing a substituted phenyl ring as the R(Ar) group were found to exhibit higher activity (except for the 4-bromophenyl compound). The highest antiproliferative activity among both thiadiazole (**4a**–**4j**) and triazole (**5a**–**5j**) compounds was observed in derivatives bearing 4-fluorophenyl, 4-chlorophenyl, 4-methylphenyl, 4-methoxyphenyl, and 4-nitrophenyl groups, respectively.
✓ 1,2,4-Triazole derivatives (**5a**–**5j**) generally exhibited higher antiproliferative and anti-aromatase activities than 1,3,4-thiadiazole compounds (**4a**–**4j**).
✓ Only certain 1,2,4-triazole compounds (**5b**, **5c**, **5e**, **5f** and **5g**) have demonstrated significant inhibitory activity against the aromatase enzyme.
✓ No antiproliferative or anti-aromatase activity has been observed in thiadiazole and triazole compounds bearing alkyl and alicyclic hydrocarbon (**4h**, **4i**, **4j**, **5h**, **5i** and **5j**).
✓ Compounds **5c**, **5e** and **5f** in the series were identified as the most potent aromatase inhibitors with IC_50_ values lower than 0.1 μM.
✓ The anti-aromatase activity was highest with *p*-Cl substitution as a halogen on the aromatic phenyl ring in the triazole compounds (**5a**–**5j**). Anti-aromatase activity also decreased with *p*-F substitution and disappeared with *p*-Br substitution. A similar situation was observed in the ranking of the potency of antiproliferative compounds.

### 2.3. In Silico Studies

#### 2.3.1. Molecular Docking Studies

**Figure 2:**
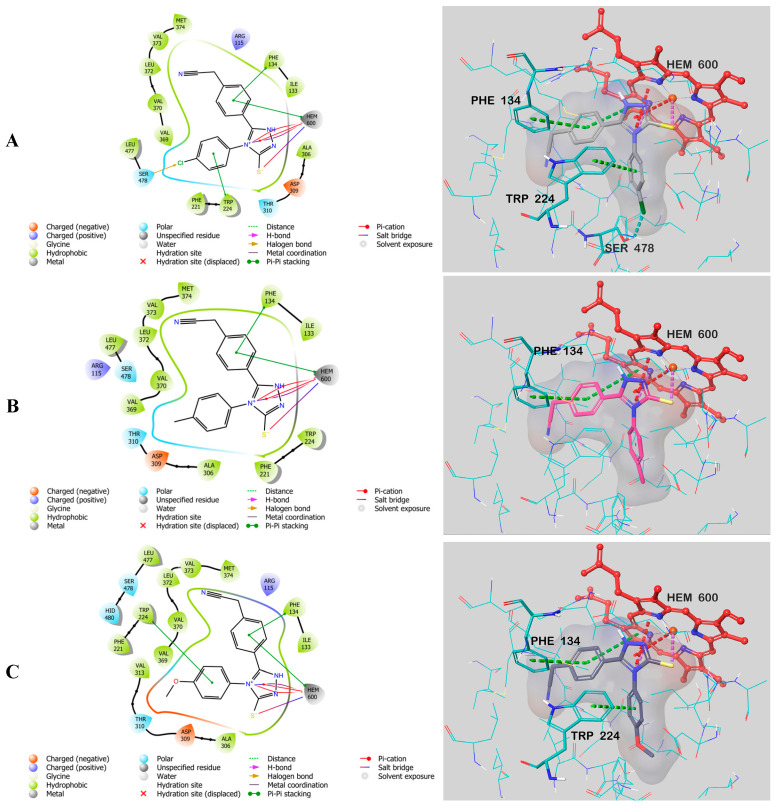
Overlay of the binding poses of compounds **5c**, **5e** and **5f** with the aromatase enzyme. Two-dimensional and three-dimensional interaction profiles of derivatives **5c** (**A**), **5e** (**B**), and **5f** (**C**) with the active site of aromatase (PDB ID: 3EQM). Note: High-resolution and enlarged versions of these figures are available in the Supplementary Material for detailed inspection (Supplementary Figure S121).

The pharmacological activity of compounds identified as effective in vitro is further evaluated in silico by analyzing their enzyme–substrate binding profiles through molecular docking studies. To this end, molecular docking simulations were initially conducted on the aromatase enzyme. Upon examination of the aromatase enzyme inhibition assay results, it was observed that several compounds exhibited varying degrees of inhibitory activity. At this stage, the three compounds demonstrating the highest inhibition potential (**5c**, **5e**, and **5f**) were selected for detailed molecular docking analysis. The resulting docking poses are presented in Figure 2 and Supplementary Figure S121. Furthermore, the interaction profiles of the selected compounds with the active site of the aromatase enzyme are detailed in Table 2.

As shown in Figure 2, the compounds evaluated in this study exhibited similar interactions. In all three compounds, the triazole ring, which was a common structural motif, formed π–π and cation–π interactions as well as a salt bridge with the HEM molecule. Additionally, a hydrogen bond was established between the nitrogen atom on the triazole ring and the HEM molecule. The sulfur atom attached to the triazole ring also formed another hydrogen bond with the HEM molecule. A π–π interaction was further detected between the HEM molecule and the phenyl ring within the structure. Moreover, this phenyl ring was involved in an additional π–π interaction with the phenyl group of Phe134. Except for compound **5e**, π–π interactions were also observed between the 4-Cl-phenyl and 4-OCH_3_-phenyl rings of compounds **5c** and **5f**, respectively, and the indole ring of Trp224. In addition to the common interactions described above, compound **5c** uniquely formed a halogen bond between the chlorine atom at the 4-position of its phenyl ring and hydroxyl of Ser478. This specific interaction may account for the slightly superior inhibitory potency of **5c** compared to the other compounds (IC_50_ = 0.064 ± 0.003 µM). Collectively, these findings suggest that the studied compounds possess strong inhibitory potential against aromatase enzymes, supporting their further investigation as promising anti-aromatase agents.

#### 2.3.2. Molecular Dynamic Studies on Aromatase Enzyme

**Figure 3:**
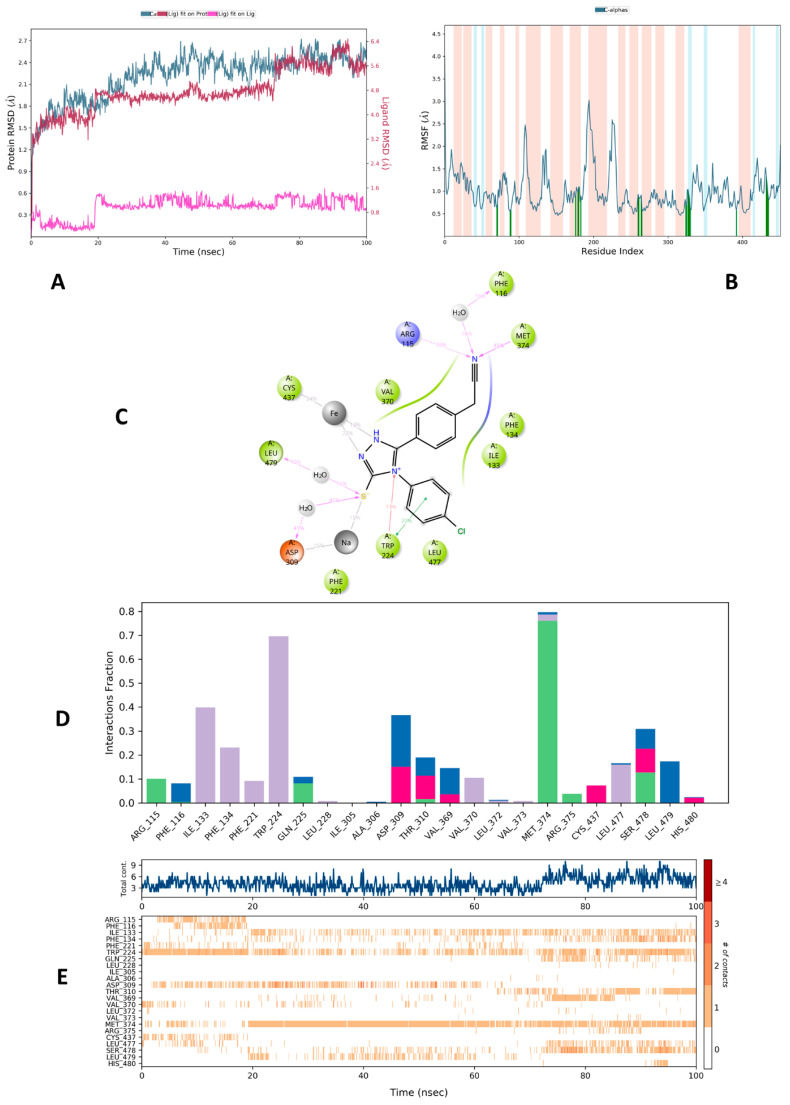
Molecular dynamic results (**A**–**E**) of compound **5c**-3EQM complex.

**Figure 4:**
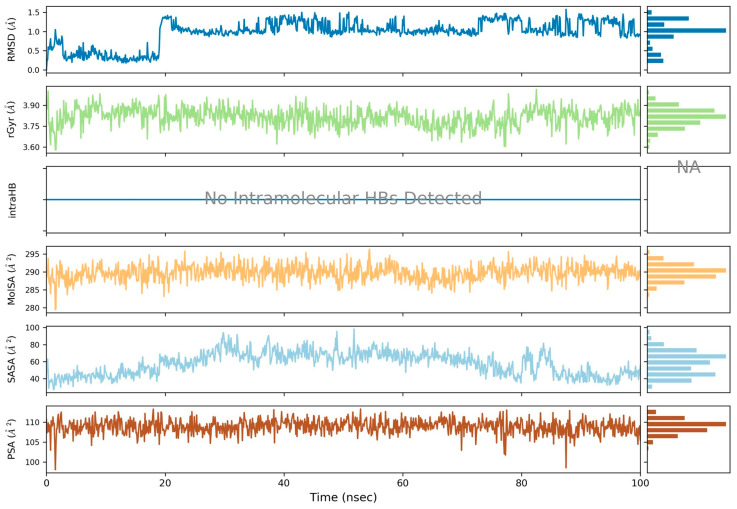
Molecular dynamic results (F) of compound **5c**-3EQM complex.

Molecular dynamics (MD) simulations were carried out for 100 ns using the crystal structures of aromatase (PDB ID: 3EQM) in conjunction with a POPE membrane model. Separate simulations were conducted for the active compound **5c**, which was identified as the most potent aromatase enzyme inhibitor. The simulation results revealed that the complexes formed between this ligand and the crystal structures of 3EQM remained structurally stable throughout the entire simulation period. Analyses of root mean square deviation (RMSD) and root mean square fluctuation (RMSF), as well as the interaction profiles of amino acid residues, are presented in Figure 3 and Figure 4, Table 3 and Table 4.

The structural stability of the ligand–enzyme complexes was evaluated using both root mean square deviation (RMSD) and root mean square fluctuation (RMSF) analyses. The overall structural stability of the protein–ligand complex throughout the simulation was first evaluated by calculating the Root Mean Square Deviation (RMSD) of the protein backbone (Cα atoms) and the ligand heavy atoms relative to their initial positions (Figure 3A). The protein Cα RMSD exhibited a gradual increase during the initial 40 ns, after which it achieved a plateau, fluctuating around a stable average of approximately 2.4 Å. This indicates that the protein underwent a period of conformational relaxation before reaching a state of dynamic equilibrium. Similarly, the RMSD of the ligand, when fitted to the protein backbone, followed the protein’s trend, stabilizing at approximately 5.6 Å, which suggests that the ligand remained securely positioned within the binding pocket while accommodating the protein’s conformational adjustments. The ligand’s internal stability was confirmed by fitting its heavy atoms to its initial coordinates, which showed a low RMSD value fluctuating around 1.1 Å after an initial conformational shift at ~20 ns. Collectively, the RMSD data confirm that the protein–ligand complex achieved and maintained a stable equilibrium throughout the latter half of the simulation.

To further investigate the dynamic behavior and flexibility of the protein backbone upon ligand binding, the Root Mean Square Fluctuation (RMSF) of each residue in the **5c**-3EQM complex was calculated (Figure 3B). The resulting RMSF profile revealed that residues located within secondary structural elements (α-helices and β-sheets) exhibited minimal fluctuations, typically below 1.5 Å, which is indicative of their structural rigidity. In contrast, the highest fluctuations were observed in the loop regions, consistent with their inherently greater conformational freedom. Notably, the majority of the active site residues that form direct interactions with compound **5c** (summarized in Table 3) displayed low RMSF values. This finding strongly suggests that the binding of the ligand confers a significant degree of stabilization to the binding pocket. By restricting the conformational mobility of these key residues, the ligand promotes a highly stable and persistent binding mode throughout the simulation.

A detailed analysis of the protein–ligand interactions was conducted to identify the key residues responsible for anchoring the ligand in the binding pocket. The interactions were monitored throughout the 100 ns trajectory, and their frequencies are summarized in Figure 3C–E.

A detailed analysis of the binding profile was conducted to identify the key interactions stabilizing compound **5c** within the aromatase active site. The two-dimensional interaction schematic (Figure 3C) and a comprehensive summary of all observed interactions (Table 4) reveal a persistent network of contacts. The ligand was found to interact consistently with several critical active site residues, including Arg115, Phe116, Trp224, Asp309, Met374, Cys437 and Leu479. Significant contributions to the binding affinity arise from specific hydrogen bonds and non-polar contacts. Critical hydrogen bonds were formed with the side chains of Asp309 (showing ~37% occupancy) and Arg115 (33% occupancy). The complex was further stabilized by several water-mediated contacts, particularly with Asp309. Concurrently, the binding was anchored by hydrophobic interactions with residues such as Phe134 (~40% occupancy) and Ile133 (~23% occupancy). As visualized in the timeline plot, the consistent presence of this specific set of contacts confirms that compound **5c** maintains a well-defined and persistent binding mode throughout the simulation.

Figure 3D displays the interaction fractions observed throughout the simulation, classified by interaction type: water-mediated hydrogen bonds (blue), direct hydrogen bonds (green), ionic interactions (pink), and hydrophobic contacts (purple). These visualizations highlight the predominant interactions formed between compound **5c** and key residues within the active site of the respective enzyme. Detailed numerical data corresponding to these interactions are provided in Table 4. These interactions further support the structural stability and favorable binding conformations of the aromatase enzyme complex formed by compound **5c**.

Figure 3E reveals the number of interactions and the corresponding residue interaction profiles throughout the simulation. Analysis of Figure 3E, corresponding to the **5c**-3EQM complex, revealed that the ligand consistently interacted with key residues in the aromatase active site, including Ile133, Trp224, Asp309, and Met374. Persistent interactions with these residues reflect the binding stability of **5c** and its potential as a strong aromatase inhibitor.

Finally, to assess the conformational stability of compound **5c** upon binding, its intrinsic structural properties were analyzed (Figure 4). The Radius of Gyration (rGyr), a key parameter measuring the ligand’s compactness, was evaluated for the **5c**-3EQM complex. The rGyr profile remained highly stable throughout the simulation, fluctuating tightly within a narrow range of 3.7 Å to 3.9 Å (Table 3), with an average value of approximately 3.8 Å. This lack of significant deviation indicates that the ligand maintained a compact conformation, without experiencing any unfolding events or structural instability within the binding site. This conclusion is further corroborated by the stable profiles of other ligand properties, including the Molecular Surface Area (MolSA), Solvent Accessible Surface Area (SASA), and Polar Surface Area (PSA). The consistent fluctuation of these metrics around stable mean values demonstrates that compound **5c** adopted a favorable and energetically stable conformation within the binding pocket, free from significant internal strain.

In conclusion, the 100 ns molecular dynamics simulation of the **5c**-3EQM complex provides strong evidence that compound **5c** adopts and maintains a stable binding conformation within the active site of the aromatase enzyme. This structural stability is comprehensively supported by consistent RMSD, RMSF, and Radius of Gyration (rGyr) values, all of which reached equilibrium and exhibited low fluctuations. The binding is driven by a persistent combination of hydrophobic and π-stacking interactions, primarily with key residues Met374 and Trp224, and is further anchored by key hydrogen bonds with catalytic residues such as Asp309. The minimal fluctuations and the consistent interaction profile confirm that the ligand remains tightly bound, validating the observed binding pose as a highly favorable one. Overall, these findings demonstrate that compound **5c** not only exhibits favorable initial binding but also retains structural stability over time, highlighting it as a promising candidate for further investigation in the development of novel, enzyme-targeted therapeutics.

#### 2.3.3. ADME Prediction

In order for active compounds to be developed as new drugs, certain physicochemical, pharmacokinetic, and drug-like properties must be evaluated beforehand [17]. Thus, some problems that may be experienced in the later stages of drug discovery processes will be prevented. In this context, some physicochemical and pharmacokinetic properties of synthesized 1,3,4-thiadiazole (**4a**–**4j**) and 1,2,4-triazole compounds (**5a**–**5j**) were calculated using SwissAdme online tools. As a result of the physicochemical parameters given in Table 5, it was observed that all compounds were in accordance with drug-likeness properties such as Lipinski, Ghose, Veber, Egan, and Muegge. Of these compounds, only **4g** and **5g** show one deviation from the Egan rule due to the high TPSA value of the nitro group. On the other hand, log *S* values, a measure of water solubility properties, indicate that the compounds are moderately soluble or soluble.

**Figure 5:**
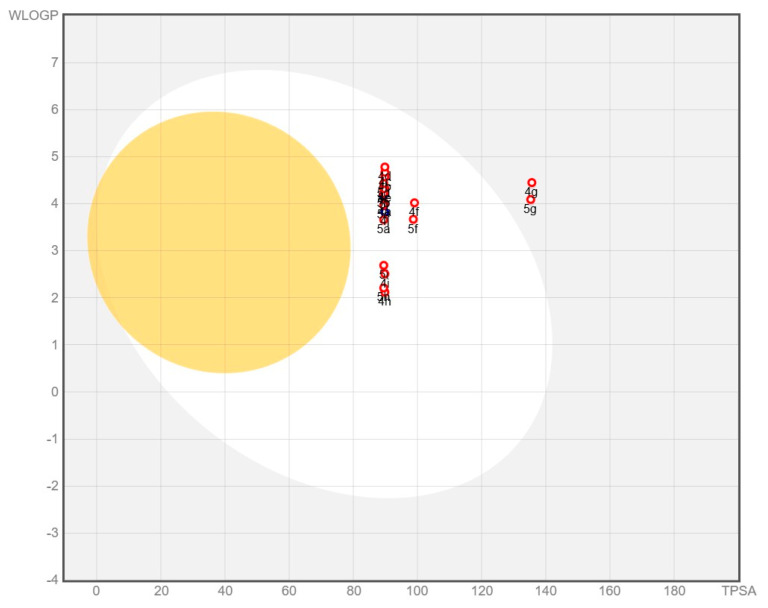
The BOILED–Egg model of all synthesized compounds.

The BOILED-Egg model obtained when the gastrointestinal and blood–brain barrier permeation of the compounds were evaluated is shown in Figure 5. Accordingly, it was determined that all of the compounds (except **4g** and **5g**) had high gastrointestinal permeability because they were found in the white area. The gastrointestinal passage of **4g** and **5g** carrying bulky nitro groups in the gray area was found to be low. In addition, as seen in Figure 5, none of the compounds entered the yellow area, so there was no blood–brain barrier permeation. The inability of these compounds to cross the blood–brain barrier is a positive feature in terms of central nervous system depression or a low side effect profile [18].

## 3. Materials and Methods

### 3.1. Chemistry

All chemicals used in this study were obtained from Sigma-Aldrich. The status of the reactions was monitored by thin-layer chromatography (TLC). For this, silica gel as a stationary phase and a dichloromethane/methanol (1:1, *v*/*v*) mixture as mobile a phase were preferred, and all spots were determined under 254 nm UV light. The IR spectra of the compounds were obtained with a Shimadzu FTIR-8400S spectrophotometer. ^1^H-NMR and ^13^C-NMR spectra of the compounds were taken with a Bruker Avance III HD 600 instrument against an internal reference standard of TMS in deuterodimethylsulfoxide. Elemental analyses were performed by a Leco CHNS-932 apparatus.

*General synthesis method of hydrazide compound (* **2** *)*

Methyl 4-(cyanomethyl)benzoate (1 mmol, 0.35 g) is dissolved in 10 mL of ethanol. 3 mmol of Hydrazine monohydrate (0.3 mL) is added to this mixture. The mixture is refluxed for 6 h, and the reaction is terminated by TLC [19].

4-(Cyanomethyl)benzohydrazide (2) CAS number: 1388215-23-3) was previously synthesized in our study [20].

*General synthesis method of thiosemicarbazide derivatives (***3a**–**3j***)*

4-(Cyanomethyl)benzohydrazide (2 mmol, 0.35 g) is dissolved in 15 mL of ethanol by heating. Isothiocyanate derivatives in equal molar ratios are added dropwise. The mixture is refluxed for 8–10 h. The excess solvent is evaporated under vacuum to give the solid thiosemicarbazides [21].

2-[4-(Cyanomethyl)benzoyl]-N-phenylhydrazine-1-carbothioamide (**3a**)

Yield: 93%; Color: white powder; Melting Point: 172.0–172.7 °C; FT-IR (cm^−1^): 3319 and 3277 (N-H), 3047 (=C-H), 2993 and 2953 (C-H), 2247 (C≡N), 1668 (C=O), 1220 (C=S). ^1^H NMR (DMSO-*d*_6_, 300 MHz, *δ* ppm): 10.59 (s, 1H, Ar-NH-CS), 9.83 and 9.74 (2s, 2H, CO-NHNH-CS), 7.99 (d, *J* = 8.0 Hz, 2H, Ar-H), 7.49 (d, *J* = 8.1 Hz, 3H, Ar-H), 7.45 (s, 1H, Ar-H), 7.34 (t, *J* = 7.8 Hz, 2H, Ar-H), 7.23–7.11 (m, 1H, Ar-H), 4.15 (s, 2H, Ar-CH_2_). ^13^C NMR (DMSO-*d*_6_, 75 MHz, *δ* ppm): 180.64 (C=S), 165.49 (C=O), 139.19, 135.17, 131.86, 128.52, 127.94, 118.90 (C≡N), 22.33 (Ar-CH_2_). Anal. calcd for C_16_H_14_N_4_OS: C 61.92, H 4.55, N 18.05, S 10.33%. Found: C 61.60, H 4.52, N 18.10, S 10.37%.

2-[4-(Cyanomethyl)benzoyl]-*N*-(4-fluorophenyl)hydrazine-1-carbothioamide (**3b**)

Yield: 79%; Color: white powder; Melting Point: 179.6–180.5 °C; FT-IR (cm^−1^): 3313 and 3174 (N-H), 3045 (=C-H), 2993 (C-H), 2251 (C≡N), 1664 (C=O), 1207 (C=S). ^1^H NMR (DMSO-*d*_6_, 300 MHz, *δ* ppm): 10.60 (s, 1H, Ar-NH-CS), 9.82 (s, 2H, CO-NHNH-CS), 8.00 (d, *J* = 6.0 Hz, 2H, Ar-H), 7.54–7.37 (m, 4H, Ar-H), 7.17 (t, *J* = 9.0 Hz, 2H, Ar-H), 4.15 (s, 2H, Ar-CH_2_). ^13^C-APT NMR (DMSO-*d*_6_, 150 MHz, *δ* ppm): 183.27 (C=S), 166.02 (C=O), 159.96 (d, *J* = 241.5 Hz), 136.03 (d, *J* = 3.0 Hz), 135.69, 132.35, 129.02, 128.44 (d, *J* = 12.0 Hz), 128.08, 119.36 (C≡N), 115.12 (d, *J* = 21.0 Hz), 22.85 (Ar-CH_2_). Anal. calcd for C_16_H_13_FN_4_OS: C 58.53, H 3.99, N 17.06, S 9.76%. Found: C 58.99, H 3.95, N 17.71, S 9.46%.

2-[4-(Cyanomethyl)benzoyl]-*N*-(4-chlorophenyl)hydrazine-1-carbothioamide (**3c**)

Yield: 80%; Color: yellow powder; Melting Point: 201.4–202.0 °C; FT-IR (cm^−1^): 3321 and 3213 (N-H), 3016 (=C-H), 2914 (C-H), 2249 (C≡N), 1668 (C=O), 1217 (C=S). ^1^H NMR (DMSO-*d*_6_, 300 MHz, *δ* ppm): 10.62 (s, 1H, Ar-NH-CS), 9.87 (s, 2H, CO-NHNH-CS), 7.99 (d, *J* = 6.0 Hz, 2H, Ar-H), 7.50 (d, *J* = 6.0 Hz, 4H, Ar-H), 7.40 (d, *J* = 9.0 Hz, 2H, Ar-H), 4.15 (s, 2H, Ar-CH_2_). ^13^C-APT NMR (DMSO-*d*_6_, 150 MHz, *δ* ppm): 181.62 (C=S), 166.03 (C=O), 138.71, 135.73, 132.31, 129.02, 128.47, 128.08, 119.35 (C≡N), 22.87 (Ar-CH_2_). Anal. calcd for C_16_H_13_ClN_4_OS: C 55.73, H 3.80, N 16.25, S 9.30%. Found: C 54.69, H 3.74, N 16.37, S 9.45%.

2-[4-(Cyanomethyl)benzoyl]-*N*-(4-bromophenyl)hydrazine-1-carbothioamide (**3d**)

Yield: 86%; Color: yellow powder; Melting Point: 201.0–201.7 °C; FT-IR (cm^−1^): 3325 and 3207 (N-H), 3010 (=C-H), 2958 and 2906 (C-H), 2252 (C≡N), 1668 (C=O), 1217 (C=S). ^1^H NMR (DMSO-*d*_6_, 300 MHz, *δ* ppm): 10.61 (s, 1H, Ar-NH-CS), 9.86 (s, 2H, CO-NHNH-CS), 7.99 (d, *J* = 8.2 Hz, 2H, Ar-H), 7.50 (q, *J* = 8.0, 7.1 Hz, 6H, Ar-H), 4.15 (s, 2H, Ar-CH_2_). ^13^C NMR (DMSO-*d*_6_, 75 MHz, *δ* ppm): 181.01 (C=S), 165.49 (C=O), 138.63, 135.24, 131.77, 130.80, 128.51, 127.97, 118.89 (C≡N), 22.35 (Ar-CH_2_). Anal. calcd for C_16_H_13_BrN_4_OS: C 49.37, H 3.37, N 14.39, S 8.24%. Found: C 49.05, H 3.43, N 14.45, S 8.17%.

2-[4-(Cyanomethyl)benzoyl]-*N*-(*p*-tolyl)hydrazine-1-carbothioamide (**3e**)

Yield: 85%; Color: white powder; Melting Point: 144.6–145.9 °C; FT-IR (cm^−1^): 3308 and 3211 (N-H), 3005 (=C-H), 2918 (C-H), 2256 (C≡N), 1664 (C=O), 1219 (C=S). ^1^H NMR (DMSO-*d*_6_, 300 MHz, *δ* ppm): 10.56 (s, 1H, Ar-NH-CS), 9.76 and 9.66 (2s, 2H, CO-NHNH-CS), 7.98 (d, *J* = 7.9 Hz, 2H, Ar-H), 7.49 (d, *J* = 7.9 Hz, 2H, Ar-H), 7.31 (d, *J* = 8.0 Hz, 2H, Ar-H), 7.14 (d, *J* = 8.0 Hz, 2H, Ar-H), 4.15 (s, 2H, Ar-CH_2_), 2.29 (s, 3H, CH_3_). ^13^C NMR (DMSO-*d*_6_, 75 MHz, *δ* ppm): 180.91 (C=S), 165.49 (C=O), 136.60, 135.15, 134.19, 131.89, 128.50, 127.92, 118.90 (C≡N), 22.33 (Ar-CH_2_), 20.53 (Ar-CH_3_). Anal. calcd for C_17_H_16_N_4_OS: C 62.94, H 4.97, N 17.27, S 9.88%. Found: C 63.67, H 5.00, N 17.15, S 9.95%.

2-[4-(Cyanomethyl)benzoyl]-*N*-(4-methoxyphenyl)hydrazine-1-carbothioamide (**3f**)

Yield: 86%; Color: white powder; Melting Point: 151.3–152.5 °C; FT-IR (cm^−1^): 3298 and 3198 (N-H), 3010 (=C-H), 2995 and 2835 (C-H), 2249 (C≡N), 1670 (C=O), 1238 (C=S). ^1^H NMR (DMSO-*d*_6_, 300 MHz, *δ* ppm): 10.55 (s, 1H, Ar-NH-CS), 9.72 and 9.62 (2s, 2H, CO-NHNH-CS), 7.98 (d, *J* = 9.0 Hz, 2H, Ar-H), 7.48 (d, *J* = 9.0 Hz, 2H, Ar-H), 7.28 (d, *J* = 9.0 Hz, 2H, Ar-H), 6.90 (d, *J* = 9.0 Hz, 2H, Ar-H), 4.15 (s, 2H, Ar-CH_2_), 3.75 (s, 3H, OCH_3_). ^13^C NMR (DMSO-*d*_6_, 75 MHz, *δ* ppm): 180.95 (C=S), 165.52 (C=O), 156.74, 135.13, 131.95, 128.52, 127.91, 118.90 (C≡N), 113.20, 55.17 (Ar-OCH_3_), 22.33 (Ar-CH_2_). Anal. calcd for C_17_H_16_N_4_O_2_S: C 59.98, H 4.74, N 16.46, S 9.42%. Found: C 59.34, H 4.78, N 16.51, S 9.47%.

2-[4-(Cyanomethyl)benzoyl]-*N*-(4-nitrophenyl)hydrazine-1-carbothioamide (**3g**)

Yield: 92%; Color: orange powder; Melting Point: 184.2–185.5 °C; FT-IR (cm^−1^): 3311 and 3184 (N-H), 3099 (=C-H), 2960 and 2850 (C-H), 2251 (C≡N), 1662 (C=O), 1219 (C=S). ^1^H NMR (DMSO-*d*_6_, 300 MHz, *δ* ppm): 10.70 (s, 1H, Ar-NH-CS), 10.15 (s, 2H, CO-NHNH-CS), 8.20 (d, *J* = 9.0 Hz, 2H, Ar-H), 7.95 (d, *J* = 8.6 Hz, 4H), 7.50 (d, *J* = 9.0 Hz, 2H, Ar-H), 4.16 (s, 2H, Ar-CH_2_). ^13^C NMR (DMSO-*d*_6_, 75 MHz, *δ* ppm): 180.74 (C=S), 165.57 (C=O), 145.46, 144.16, 135.36, 132.32, 128.47, 128.05, 123.56, 118.88 (C≡N), 22.34 (Ar-CH_2_). Anal. calcd for C_16_H_13_N_5_O_3_S: C 54.08, H 3.69, N 19.71, S 9.02%. Found: C 53.75, H 3.71, N 19.78, S 9.07%.

2-[4-(Cyanomethyl)benzoyl]-*N*-methylhydrazine-1-carbothioamide (**3h**)

Yield: 92%; Color: lilac powder; Melting Point: 156.3–157.0 °C; FT-IR (cm^−1^): 3342 and 3230 (N-H), 3025 (=C-H), 2978 and 2937 (C-H), 2245 (C≡N), 1662 (C=O), 1219 (C=S). ^1^H NMR (DMSO-*d*_6_, 300 MHz, *δ* ppm): 10.38 (s, 1H, CO-NHNH-CS), 9.34 (s, 1H, CO-NHNH-CS), 8.06 (q, *J* = 4.3 Hz, 1H, CS-NH-CH_3_), 7.93 (d, *J* = 9.0 Hz, 2H, Ar-H), 7.48 (d, *J* = 9.0 Hz, 2H, Ar-H), 4.15 (s, 2H, Ar-CH_2_), 2.88 (d, *J* = 4.4 Hz, 3H, NH-CH_3_). ^13^C NMR (DMSO-*d*_6_, 75 MHz, *δ* ppm): 182.25 (C=S), 165.52 (C=O), 135.14, 131.81, 128.43, 127.93, 118.90 (C≡N), 30.91 (NH-CH_3_), 22.32 (Ar-CH_2_). Anal. calcd for C_11_H_12_N_4_OS: C 53.21, H 4.87, N 22.56, S 12.91%. Found: C 53.88, H 4.84, N 22.65, S 12.85%.

2-[4-(Cyanomethyl)benzoyl]-*N*-ethylhydrazine-1-carbothioamide (**3i**)

Yield: 95%; Color: white powder; Melting Point: 210.0–210.7 °C; FT-IR (cm^−1^): 3311 and 3254 (N-H), 3093 (=C-H), 2980 and 2937 (C-H), 2243 (C≡N), 1662 (C=O), 1213 (C=S). ^1^H NMR (DMSO-*d*_6_, 300 MHz, *δ* ppm): 10.35 (s, 1H, CO-NHNH-CS), 9.27 (s, 1H, CO-NHNH-CS), 8.11 (t, *J* = 5.7 Hz, 1H, CS-NH-CH_2_CH_3_), 7.94 (d, *J* = 9.0 Hz, 2H, Ar-H), 7.47 (d, *J* = 9.0 Hz, 2H, Ar-H), 4.14 (s, 2H, Ar-CH_2_), 3.55–3.40 (m, 2H, NH-CH_2_CH_3_), 1.07 (t, *J* = 7.1 Hz, 3H, NH-CH_2_CH_3_). ^13^C NMR (DMSO-*d*_6_, 75 MHz, *δ* ppm): 181.84 (C=S), 165.45 (C=O), 135.14, 131.83, 128.45, 127.93, 118.89 (C≡N), 38.47 (NH-CH_2_CH_3_), 22.32 (Ar-CH_2_), 14.46 (NH-CH_2_CH_3_). Anal. calcd for C_12_H_14_N_4_OS: C 54.94, H 5.38, N 21.36, S 12.22%. Found: C 55.53, H 5.30, N 21.50, S 12.10%.

2-[4-(Cyanomethyl)benzoyl]-*N*-cyclohexylhydrazine-1-carbothioamide (**3j**)

Yield: 98%; Color: lilac powder; Melting Point: 207.1–207.9 °C; FT-IR (cm^−1^): 3211 and 3130 (N-H), 2926, 2852 (=C-H and C-H), 2256 (C≡N), 1678 (C=O), 1230 (C=S). ^1^H NMR (DMSO-*d*_6_, 300 MHz, *δ* ppm): 10.31 (s, 1H, CO-NHNH-CS), 9.22 (s, 1H, CO-NHNH-CS), 7.94 (d, *J* = 9.0 Hz, 2H, Ar-H), 7.74 (d, *J* = 8.3 Hz, 1H, CS-NH-cyclohexyl), 7.48 (d, *J* = 9.0 Hz, 2H, Ar-H), 4.14 (s, 2H, Ar-CH_2_), 1.80 (d, *J* = 8.9 Hz, 2H, cyclohexyl protons), 1.69 (dd, *J* = 9.5, 4.2 Hz, 2H, cyclohexyl protons), 1.58 (d, *J* = 12.2 Hz, 1H, cyclohexyl protons), 1.38–1.15 (m, 5H, cyclohexyl protons), 1.14–1.01 (m, 1H, cyclohexyl protons). ^13^C-APT NMR (DMSO-*d*_6_, 150 MHz, *δ* ppm): 184.84 (C=S), 165.85 (C=O), 135.62, 132.41, 128.92, 128.46, 119.35 (C≡N), 53.48, 32.31, 25.65, 25.39 (cyclohexyl carbons), 22.83 (Ar-CH_2_). Anal. calcd for C_16_H_20_N_4_OS: C 60.73, H 6.37, N 17.71, S 10.13%. Found: C 60.19, H 6.29, N 17.79, S 10.01%.

*General synthesis method of 2,5-disubstituted-1,3,4-thiadiazol-5-amine (***4a**–**4j***)*

Weigh 1 mmol of thiosemicarbazide derivatives and put into a beaker. Concentrated sulfuric acid (1–1.5 mL) is added dropwise until the substance is dissolved. The mixture is stirred at room temperature for 4 h. The mixture is poured into ice-water and neutralized with a dilute base solution to obtain thiadiazole derivatives. The solid material is crystallized from ethanol [22].

2-{4-[5-(Phenylamino)-1,3,4-thiadiazol-2-yl]phenyl}acetonitrile (**4a**)

Yield: 80%; Color: orange powder; Melting Point: 270.0–270.5 °C; FT-IR (cm^−1^): 3333 and 3184 (N-H), 3053 (=C-H), 2947 and 2890 (C-H), 2366 (C≡N), 1658 (C=N). ^1^H NMR (DMSO-*d*_6_, 300 MHz, *δ* ppm): 10.57 (s, 1H, NH), 7.92–7.76 (m, 2H, Ar-H), 7.70–7.59 (m, 2H, Ar-H), 7.45–7.32 (m, 3H, Ar-H), 7.15–6.91 (m, 2H, Ar-H), 4.38 (s, 2H, CH_2_CN). ^13^C NMR (DMSO-*d*_6_, 150 MHz, *δ* ppm): 172.26 (C=N), 164.43, 158.23 (C=N), 142.45, 141.03, 139.34, 130.43, 129.61, 128.92, 127.08, 122.54, 118.01 (C≡N), 116.89, 42.44 (Ar-CH_2_). Anal. calcd for C_16_H_12_N_4_S: C 65.73, H 4.14, N 19.16, S 10.97%. Found: C 65.10, H 4.19, N 19.29, S 10.87%.

2-(4-{5-[(4-Fluorophenyl)amino]-1,3,4-thiadiazol-2-yl}phenyl)acetonitrile (**4b**)

Yield: 75%; Color: white powder; Melting Point: 192.7–194.0 °C; FT-IR (cm^−1^): 3352 and 3146 (N-H), 3039 (=C-H), 2899 and 2891 (C-H), 2258 (C≡N), 1651 and 1606 (C=N). ^1^H NMR (DMSO-*d*_6_, 300 MHz, *δ* ppm): 10.55 (s, 1H, NH), 7.92–7.86 (m, 1H, Ar-H), 7.82–7.75 (m, 1H, Ar-H), 7.75–7.65 (m, 1H, Ar-H), 7.50–7.47 (m, 2H, Ar-H), 7.43–7.37 (m, 1H, Ar-H), 7.28–7.16 (m, 2H, Ar-H), 4.14 (s, 2H, CH_2_CN). ^13^C NMR (DMSO-*d*_6_, 75 MHz, *δ* ppm): 171.72 (C=N), 163.97, 157.51 (C=N), 138.85, 137.01 (d, *J* = 2.25 Hz), 129.92, 129.59, 128.93, 128.37, 127.24, 126.58, 119.12 (C≡N), 115.65 (d, *J* = 21.75 Hz), 41.94 (CH_2_CN). Anal. calcd for C_16_H_11_FN_4_S: C 61.92, H 3.57, N 18.05, S 10.33%. Found: C 62.55, H 3.53, N 18.09, S 10.25%.

2-(4-{5-[(4-Chlorophenyl)amino]-1,3,4-thiadiazol-2-yl}phenyl)acetonitrile (**4c**)

Yield: 80%; Color: white powder; Melting Point: 216.9–217.5 °C; FT-IR (cm^−1^): 3236 and 3180 (N-H), 3030 (=C-H), 2995 and 2937 (C-H), 2251 (C≡N), 1666 and 1599 (C=N). ^1^H NMR (DMSO-*d*_6_, 300 MHz, *δ* ppm): 10.69 (s, 1H, NH), 7.85 (d, *J* = 8.1 Hz, 2H, Ar-H), 7.75–7.67 (m, 2H, Ar-H), 7.46–7.34 (m, 3H, Ar-H), 6.96 (s, 1H, Ar-H), 4.14 (s, 2H, CH_2_CN). ^13^C NMR (DMSO-*d*_6_, 150 MHz, *δ* ppm): 172.22 (C=N), 164.05, 158.45 (C=N), 139.91, 139.46, 133.98, 130.44, 130.02, 129.45, 128.81, 127.81, 127.14, 125.90, 119.55 (C≡N), 42.60 (CH_2_CN). Anal. calcd for C_16_H_11_ClN_4_S: C 58.81, H 3.39, N 17.14, S 9.81%. Found: C 58.67, H 3.40, N 17.17, S 9.76%.

2-(4-{5-[(4-Bromophenyl)amino]-1,3,4-thiadiazol-2-yl}phenyl)acetonitrile (**4d**)

Yield: 80%; Color: yellow powder; Melting Point: 211.6–212.7 °C; FT-IR (cm^−1^): 3236 and 3176 (N-H), 3030 (=C-H), 2945 and 2850 (C-H), 2366 (C≡N), 1662 (C=N). ^1^H NMR (DMSO-*d*_6_, 300 MHz, *δ* ppm): 10.67 (s, 1H, NH), 7.89–7.77 (m, 2H, Ar-H), 7.70–7.62 (m, 2H, Ar-H), 7.57–7.50 (m, 2H, Ar-H), 7.43–7.36 (m, 2H, Ar-H), 4.40 (s, 2H, CH_2_CN). ^13^C NMR (DMSO-*d*_6_, 150 MHz, *δ* ppm): 171.72 (C=N), 163.48, 157.99 (C=N), 139.79, 138.97, 131.79, 129.94, 128.27, 126.64, 119.39 (C≡N), 113.24, 41.94 (Ar-CH_2_). Anal. calcd for C_16_H_11_BrN_4_S: C 51.76, H 2.99, N 15.09, S 8.64%. Found: C 51.42, H 3.01, N 15.01, S 8.74%.

2-(4-{5-[(4-Methylphenyl)amino]-1,3,4-thiadiazol-2-yl}phenyl)acetonitrile (**4e**)

Yield: 75%; Color: yellow powder; Melting Point: 212.2–213.2 °C; FT-IR (cm^−1^): 3342 and 3174 (N-H), 3030 (=C-H), 2916 and 2858 (C-H), 2254 (C≡N), 1633 and 1610 (C=N). ^1^H NMR (DMSO-*d*_6_, 300 MHz, *δ* ppm): 10.44 (s, 1H, NH), 7.91–7.75 (m, 2H, Ar-H), 7.59–7.51 (m, 2H, Ar-H), 7.40 (d, *J* = 8.3 Hz, 2H, Ar-H), 7.23–7.14 (m, 2H, Ar-H), 4.24 (s, 2H, CH_2_CN), 2.27 (s, 3H, CH_3_). ^13^C NMR (DMSO-*d*_6_, 150 MHz, *δ* ppm): 172.26 (C=N), 164.66, 157.86 (C=N), 139.25, 138.65, 133.75, 131.57, 130.41, 130.01, 129.42, 128.98, 127.69, 127.03, 118.18 (C≡N), 42.45 (CH_2_CN), 20.84 (Ar-CH_3_). Anal. calcd for C_17_H_14_N_4_S: C 66.64, H 4.61, N 18.29, S 10.46%. Found: C 66.97, H 4.65, N 18.37, S 10.41%.

2-(4-{5-[(4-Methoxyphenyl)amino]-1,3,4-thiadiazol-2-yl}phenyl)acetonitrile (**4f**)

Yield: 77%; Color: lilac powder; Melting Point: 198.6–199.9 °C; FT-IR (cm^−1^): 3335 and 3173 (N-H), 3053 (=C-H), 2953 and 2835 (C-H), 2283 (C≡N), 1656 and 1599 (C=N). ^1^H NMR (DMSO-*d*_6_, 300 MHz, *δ* ppm): 10.34 (s, 1H, NH), 7.90–7.73 (m, 2H, Ar-H), 7.62–7.52 (m, 2H, Ar-H), 7.51–7.35 (m, 2H, Ar-H), 6.99–6.93 (m, 2H, Ar-H), 4.13 (s, 2H, CH_2_CN), 3.75 (s, 3H, OCH_3_). ^13^C NMR (DMSO-*d*_6_, 150 MHz, *δ* ppm): 172.30 (C=N), 165.14, 156.72 (C=N), 155.02, 139.15, 134.46, 133.66, 130.75, 129.41, 129.05, 127.63, 127.03, 119.79 (C≡N), 114.85, 55.70 (Ar-OCH_3_), 42.44 (CH_2_CN). Anal. calcd for C_17_H_14_N_4_OS: C 63.34, H 4.38, N 17.38, S 9.94%. Found: C 63.78, H 4.42, N 17.30, S 9.99%.

2-(4-{5-[(4-Nitrophenyl)amino]-1,3,4-thiadiazol-2-yl}phenyl)acetonitrile (**4g**)

Yield: 85%; Color: yellow powder; Melting Point: 225.0–226.2 °C; FT-IR (cm^−1^): 3335 and 3186 (N-H), 3053 (=C-H), 2951 and 2835 (C-H), 2258 (C≡N), 1658 and 1608 (C=N). ^1^H NMR (DMSO-*d*_6_, 300 MHz, *δ* ppm): 11.62 and 11.28 (2s, 1H, NH), 8.32–8.22 (m, 2H, Ar-H), 7.88–7.81 (m, 3H, Ar-H), 7.47–7.36 (m, 2H, Ar-H), 6.95 (d, *J* = 8.9 Hz, 1H, Ar-H), 4.40 (s, 2H, CH_2_CN). ^13^C NMR (DMSO-*d*_6_, 150 MHz, *δ* ppm): 172.21 (C=N), 163.27, 160.14 (C=N), 146.68, 141.35, 139.84, 130.86, 130.51, 129.68, 128.50, 127.38, 125.98, 117.49 (C≡N), 42.58 (CH_2_CN). Anal. calcd for C_16_H_11_N_5_O_2_S: C 56.97, H 3.29, N 20.76, S 9.50%. Found: C 56.67, H 3.25, N 20.71, S 9.54%.

2-{4-[5-(Methylamino)-1,3,4-thiadiazol-2-yl]phenyl}acetonitrile (**4h**)

Yield: 85%; Color: white powder; Melting Point: 204.4–205.6 °C; FT-IR (cm^−1^): 3336 and 3161 (N-H), 3028 (=C-H), 2978 and 2879 (C-H), 2330 (C≡N), 1633 (C=N). ^1^H NMR (DMSO-*d*_6_, 300 MHz, *δ* ppm): 7.90–7.83 (m, 1H, NH), 7.77–7.64 (m, 2H, Ar-H), 7.53–7.32 (m, 2H, Ar-H), 3.47 (s, 2H, CH_2_CN), 2.93 (d, *J* = 4.4 Hz, 3H, CH_3_). ^13^C NMR (DMSO-*d*_6_, 150 MHz, *δ* ppm): 172.32 (C=N), 169.62, 156.27 (C=N), 138.65, 130.31, 129.52, 126.67, 42.41 (CH_2_CN), 31.79 (CH_3_). Anal. calcd for C_11_H_10_N_4_S: C 57.37, H 4.38, N 24.33, S 13.92%. Found: C 57.88, H 4.33, N 24.43, S 13.83%.

2-{4-[5-(Ethylamino)-1,3,4-thiadiazol-2-yl]phenyl}acetonitrile (**4i**)

Yield: 82%; Color: white powder; Melting Point: 226.6–227.1 °C; FT-IR (cm^−1^): 3342 and 3163 (N-H), 3041 (=C-H), 2999 and 2866 (C-H), 2339 (C≡N), 1633 (C=N). ^1^H NMR (DMSO-*d*_6_, 300 MHz, *δ* ppm): 7.94–7.86 (m, 1H, NH), 7.73–7.64 (m, 2H, Ar-H), 7.56–7.32 (m, 2H, Ar-H), 3.47 (s, 2H, CH_2_CN), 3.43 (s, 2H, CH_2_CH_3_), 1.20 (t, *J* = 7.2 Hz, 3H, CH_2_CH_3_). ^13^C NMR (DMSO-*d*_6_, 150 MHz, *δ* ppm): 172.29 (C=N), 168.70, 156.10 (C=N), 138.63, 130.29, 129.74, 129.54, 126.62, 42.59 (Ar-CH_2_), 39.64 (CH_2_CH_3_), 14.74 (CH_2_CH_3_). Anal. calcd for C_12_H_12_N_4_S: C 58.99, H 4.95, N 22.93, S 13.12%. Found: C 59.19, H 4.97, N 23.04, S 13.01%.

2-{4-[5-(Cyclohexylamino)-1,3,4-thiadiazol-2-yl]phenyl}acetonitrile (**4j**)

Yield: 82%; Color: white powder; Melting Point: 242.5–243.3 °C; FT-IR (cm^−1^): 3288 and 3161 (N-H), 3018 (=C-H), 2939 and 2856 (C-H), 2341 (C≡N), 1678 and 1633 (C=N). ^1^H NMR (DMSO-*d*_6_, 300 MHz, *δ* ppm): 7.88 (d, *J* = 7.2 Hz, 1H, NH), 7.72–7.63 (m, 2H, Ar-H), 7.39–7.30 (m, 2H, Ar-H), 3.65–3.50 (s, 2H, CH_2_CN), 1.99 (m, 2H, cyclohexyl protons), 1.79–1.48 (m, 3H, cyclohexyl protons), 1.40–1.13 (m, 6H, cyclohexyl protons). ^13^C NMR (DMSO-*d*_6_, 150 MHz, *δ* ppm): 172.30 (C=N), 167.91, 155.85 (C=N), 138.56, 130.62, 130.28, 129.57, 126.63, 119.44 (C≡N), 54.25, 42.42 (Ar-CH_2_), 32.58, 25.71, 24.77, 22.68. Anal. calcd for C_16_H_18_N_4_S: C 64.40, H 6.08, N 18.78, S 10.74%. Found: C 64.82, H 6.00, N 18.99, S 10.67%.

*General synthesis method of 1,2-disubstituted-5-thioxo-4,5-dihydro-1H-1,2,4-triazol-3-yl (***5a**–**5j***)*

Weigh 1 mmol of thiosemicarbazide derivatives and put into a reaction flask. 2–3 mL of NaOH solution (2N) is added on it until the substance is dissolved. The mixture is refluxed at 100 °C for 6–10 h. Ice pieces are poured onto the reaction mixture and neutralization is carried out with 1N HCl to obtain the triazole derivatives in solid form. The solid residue is crystallized from ethanol [22].

2-[4-(4-Phenyl-5-thioxo-4,5-dihydro-1*H*-1,2,4-triazol-3-yl)phenyl]acetonitrile (**5a**)

Yield: 75%; Color: gray powder; Melting Point: 257.0–258.3 °C; FT-IR (cm^−1^): 3309 (N-H), 3037 (=C-H), 2978 and 2914 (C-H), 2339 (C≡N), 1707 (C=N), 1236 (C=S). ^1^H NMR (DMSO-*d*_6_, 300 MHz, *δ* ppm): 14.12 (s, 1H, NH), 7.54–7.47 (m, 3H, Ar-H), 7.39–7.34 (m, 2H, Ar-H), 7.28–7.19 (m, 4H, Ar-H), 3.57 (s, 2H, CH_2_CN). ^13^C NMR (DMSO-*d*_6_, 150 MHz, *δ* ppm): 172.18 (C=N), 168.54, 150.43 (C=N), 137.46, 134.55, 129.62, 129.41, 129.33, 128.73, 128.02, 124.07, 40.20. Anal. calcd for C_16_H_12_N_4_S: C 65.73, H 4.14, N 19.16, S 10.97%. Found: C 64.89, H 4.09, N 19.30, S 10.83%.

2-{4-[4-(4-Fluorophenyl)-5-thioxo-4,5-dihydro-1*H*-1,2,4-triazol-3-yl]phenyl}acetonitrile (**5b**)

Yield: 80%; Color: white powder; Melting Point: 244.1–245.0 °C; FT-IR (cm^−1^): 3379 and 3296 (N-H), 3039 (=C-H), 2931 and 2885 (C-H), 2254 (C≡N), 1672 (C=N), 1225 (C=S). ^1^H NMR (DMSO-*d*_6_, 300 MHz, *δ* ppm): 14.03 (s, 1H, NH), 7.49–7.41 (m, 2H, Ar-H), 7.40–7.31 (m, 2H, Ar-H), 7.26 (s, 4H, Ar-H), 3.58 (s, 2H, CH_2_CN). ^13^C NMR (DMSO-*d*_6_, 150 MHz, *δ* ppm): 172.67 (C=N), 169.16, 162.52 (d, *J* = 244.5 Hz), 150.98 (C=N), 138.06, 131.62 (d, *J* = 9.0 Hz), 131.37 (d, *J* = 3.0 Hz), 130.17, 128.62, 124.51, 116.78 (d, *J* = 22.5 Hz), 40.76. Anal. calcd for C_16_H_11_FN_4_S: C 61.92, H 3.57, N 18.05, S 10.33%. Found: C 62.60, H 3.50, N 18.19, S 10.45%.

2-{4-[4-(4-Chlorophenyl)-5-thioxo-4,5-dihydro-1*H*-1,2,4-triazol-3-yl]phenyl}acetonitrile (**5c**)

Yield: 85%; Color: white powder; Melting Point: 255.5–256.0 °C; FT-IR (cm^−1^): 3358 and 3277 (N-H), 3032 (=C-H), 2925 and 2877 (C-H), 2278 (C≡N), 1672 (C=N), 1230 (C=S). ^1^H NMR (DMSO-*d*_6_, 300 MHz, *δ* ppm): 13.92 (s, 1H, NH), 7.63–7.53 (m, 2H, Ar-H), 7.48–7.38 (m, 2H, Ar-H), 7.26 (s, 4H, Ar-H), 3.58 (s, 2H, CH_2_CN). ^13^C-APT NMR (DMSO-*d*_6_, 150 MHz, *δ* ppm): 172.62 (C=N), 169.03, 150.87 (C=N), 138.10, 134.51, 133.97, 131.20, 130.20, 129.89, 128.66, 124.44, 40.76. Anal. calcd for C_16_H_11_ClN_4_S: C 58.81, H 3.39, N 17.14, S 9.81%. Found: C 57.91, H 3.45, N 17.20, S 9.87%.

2-{4-[4-(4-Bromophenyl)-5-thioxo-4,5-dihydro-1*H*-1,2,4-triazol-3-yl]phenyl}acetonitrile (**5d**)

Yield: 80%; Color: white powder; Melting Point: 238.8–239.8 °C; FT-IR (cm^−1^): 3228 (N-H), 3097 (=C-H), 2974 and 2897 (C-H), 2272 (C≡N), 1703 (C=N), 1259 (C=S). ^1^H NMR (DMSO-*d*_6_, 300 MHz, *δ* ppm): 14.18 and 12.52 (2s, 1H, NH), 7.77–7.64 (m, 2H, Ar-H), 7.43–7.30 (m, 2H, Ar-H), 7.27 (s, 4H, Ar-H), 3.59 (s, 2H, CH_2_CN). ^13^C NMR (DMSO-*d*_6_, 150 MHz, *δ* ppm): 172.12 (C=N), 168.39, 150.30 (C=N), 137.58, 133.87, 132.34, 130.97, 129.72, 128.16, 123.91, 122.63, 40.23. Anal. calcd for C_16_H_11_BrN_4_S: C 51.76, H 2.99, N 15.09, S 8.64%. Found: C 51.08, H 3.05, N 14.98, S 8.73%.

2-{4-[4-(4-Methylphenyl)-5-thioxo-4,5-dihydro-1*H*-1,2,4-triazol-3-yl]phenyl}acetonitrile (**5e**)

Yield: 82%; Color: yellow powder; Melting Point: 304.6–305.4 °C; FT-IR (cm^−1^): 3302 (N-H), 3037 (=C-H), 2941 and 2877 (C-H), 2254 (C≡N), 1672 (C=N), 1232 (C=S). ^1^H NMR (DMSO-*d*_6_, 300 MHz, *δ* ppm): 14.09 (s, 1H, NH), 7.33–7.27 (m, 3H, Ar-H), 7.27–7.20 (m, 5H, Ar-H), 3.57 (s, 2H, CH_2_CN), 2.36 (s, 3H, CH_3_). ^13^C NMR (DMSO-*d*_6_, 150 MHz, *δ* ppm): 172.67 (C=N), 169.17, 150.97 (C=N), 139.47, 137.94, 137.82, 132.48, 130.23, 128.92, 128.52, 124.66, 118.58, 40.72, 21.26. Anal. calcd for C_17_H_14_N_4_S: C 66.64, H 4.61, N 18.29, S 10.46%. Found: C 66.16, H 4.63, N 18.22, S 10.41%.

2-{4-[4-(4-Methoxyphenyl)-5-thioxo-4,5-dihydro-1*H*-1,2,4-triazol-3-yl]phenyl}acetonitrile (**5f**)

Yield: 80%; Color: purple powder; Melting Point: 257.8–259.0 °C; FT-IR (cm^−1^): 3402 (N-H), 3076 (=C-H), 2910 and 2850 (C-H), 2312 (C≡N), 1697 (C=N), 1255 (C=S). ^1^H NMR (DMSO-*d*_6_, 300 MHz, *δ* ppm): 14.07 and 12.42 (2s, 1H, NH), 7.35–7.19 (m, 6H, Ar-H), 7.10–6.98 (m, 2H, Ar-H), 3.80 (s, 3H, OCH_3_), 3.58 (s, 2H, CH_2_CN). ^13^C NMR (DMSO-*d*_6_, 150 MHz, *δ* ppm): 172.67 (C=N), 169.34, 160.02, 151.08 (C=N), 137.88, 130.41, 130.13, 128.51, 127.58, 124.72, 114.96, 55.87, 40.69. Anal. calcd for C_17_H_14_N_4_OS: C 63.34, H 4.38, N 17.38, S 9.94%. Found: C 63.88, H 4.43, N 17.30, S 9.99%.

2-{4-[4-(4-Nitrophenyl)-5-thioxo-4,5-dihydro-1*H*-1,2,4-triazol-3-yl]phenyl}acetonitrile (**5g**)

Yield: 85%; Color: brown powder; Melting Point: 357.8–358.9 °C; FT-IR (cm^−1^): 3279 (N-H), 3053 (=C-H), 2893 and 2821 (C-H), 2312 (C≡N), 1681 (C=N), 1282 (C=S). ^1^H NMR (DMSO-*d*_6_, 300 MHz, *δ* ppm): 14.22 and 12.90 (2s, 1H, NH), 8.05 (s, 4H, Ar-H), 7.94–7.87 (m, 2H, Ar-H), 7.43–7.35 (m, 2H, Ar-H), 3.69 (s, 2H, CH_2_CN). ^13^C NMR (DMSO-*d*_6_, 150 MHz, *δ* ppm): 172.16 (C=N), 167.17, 166.64, 159.02 (C=N), 144.77, 140.11, 134.40, 129.62, 129.42, 129.22, 129.09, 125.48, 40.48. Anal. calcd for C_16_H_11_N_5_O_2_S: C 56.97, H 3.29, N 20.76, S 9.50%. Found: C 56.30, H 3.33, N 20.66, S 9.40%.

2-[4-(4-Methyl-5-thioxo-4,5-dihydro-1*H*-1,2,4-triazol-3-yl)phenyl]acetonitrile (**5h**)

Yield: 85%; Color: white powder; Melting Point: 224.5–225.3 °C; FT-IR (cm^−1^): 3294 (N-H), 3051 (=C-H), 2895 and 2870 (C-H), 2351 (C≡N), 1712 (C=N), 1213 (C=S). ^1^H NMR (DMSO-*d*_6_, 300 MHz, *δ* ppm): 13.93 and 12.53 (2s, 1H, NH), 7.75–7.63 (m, 2H, Ar-H), 7.51–7.41 (m, 2H, Ar-H), 3.69 (s, 2H, CH_2_CN), 3.54 (s, 3H, NHCH_3_). ^13^C NMR (DMSO-*d*_6_, 150 MHz, *δ* ppm): 172.29 (C=N), 167.41, 151.31 (C=N), 137.86, 130.02, 128.35, 124.36, 40.37, 31.60. Anal. calcd for C_11_H_10_N_4_S: C 57.37, H 4.38, N 24.33, S 13.92%. Found: C 56.76, H 4.45, N 24.20, S 14.05%.

2-[4-(4-Ethyl-5-thioxo-4,5-dihydro-1*H*-1,2,4-triazol-3-yl)phenyl]acetonitrile (**5i**)

Yield: 77%; Color: white powder; Melting Point: 199.5–200.5 °C; FT-IR (cm^−1^): 3225 (N-H), 3047 (=C-H), 2995 and 2858 (C-H), 2351 (C≡N), 1662 (C=N), 1255 (C=S). ^1^H NMR (DMSO-*d*_6_, 300 MHz, *δ* ppm): 13.92 and 12.49 (2s, 1H, NH), 7.68–7.58 (m, 2H, Ar-H), 7.51–7.43 (m, 2H, Ar-H), 4.04 (q, *J* = 7.1 Hz, 2H, NHCH_2_CH_3_), 3.70 (s, 2H, CH_2_CN), 1.16 (t, *J* = 7.1 Hz, 3H, NHCH_2_CH_3_). ^13^C NMR (DMSO-*d*_6_, 150 MHz, *δ* ppm): 172.28 (C=N), 166.73, 150.99 (C=N), 137.90, 130.16, 128.40, 124.40, 40.28, 38.29, 13.41. Anal. calcd for C_12_H_12_N_4_S: C 58.99, H 4.95, N 22.93, S 13.12%. Found: C 59.87, H 4.87, N 22.79, S 13.01%.

2-[4-(4-Cyclohexyl-5-thioxo-4,5-dihydro-1*H*-1,2,4-triazol-3-yl)phenyl]acetonitrile (**5j**)

Yield: 80%; Color: white powder; Melting Point: 273.1–274.3 °C; FT-IR (cm^−1^): 3200 (N-H), 3053 (=C-H), 2929 and 2854 (C-H), 2312 (C≡N), 1714 (C=N), 1294 (C=S). ^1^H NMR (DMSO-*d*_6_, 300 MHz, *δ* ppm): 13.87 and 12.50 (2s, 1H, NH), 7.47 (q, *J* = 8.3 Hz, 4H, Ar-H), 4.25 (t, *J* = 12.3 Hz, 1H, cyclohexyl proton), 3.71 (s, 2H, CH_2_CN), 2.20 (s, 2H, cyclohexyl protons), 1.72 (d, *J* = 11.9 Hz, 4H, cyclohexyl protons), 1.54 (d, *J* = 12.6 Hz, 1H, cyclohexyl proton), 1.28–1.07 (m, 2H, cyclohexyl protons), 0.94 (q, *J* = 12.9 Hz, 1H, cyclohexyl proton). ^13^C-APT NMR (DMSO-*d*_6_, 150 MHz, *δ* ppm): 172.74 (C=N), 166.65, 151.97 (C=N), 138.31, 130.29, 130.08, 125.54, 57.46, 40.84, 29.77, 25.87, 25.08. Anal. calcd for C_16_H_18_N_4_S: C 64.40, H 6.08, N 18.78, S 10.74%. Found: C 64.89, H 6.12, N 18.65, S 10.80%.

### 3.2. Biological Activity

#### 3.2.1. Cytotoxicity Assay

To evaluate the cytotoxic effects on NIH3T3 and MCF-7 cell lines, the MTT assay was employed following established protocols [23,24]. This method quantifies cell viability based on the enzymatic conversion of MTT into formazan crystals, a process indicative of metabolic activity in living cells [25].

#### 3.2.2. Aromatase Inhibition Assay

The in vitro aromatase inhibitory potential of the synthesized compounds was evaluated using a fluorometric screening kit (BioVision, CYP19A), following the methodology established in our previous studies [26,27,28]. Test compounds were prepared in 2% DMSO across a broad concentration range (10^−3^–10^−9^ M), with Letrozole serving as the reference inhibitor. Each analysis, including blanks and controls, was performed in quadruplicate, and data were expressed as mean ± standard deviation (SD). For the determination of IC_50_ values, dose–response curves were generated by plotting inhibition percentages against logarithmic concentrations using GraphPad Prism software (v. 5.0).

### 3.3. In Silico Studies

#### 3.3.1. Molecular Docking Studies

To elucidate the binding orientations of compounds **5c**, **5e**, and **5f** within the aromatase enzyme’s active site, in silico molecular docking simulations were conducted. The human aromatase X-ray crystal structure (PDB ID: 3EQM) [29] was obtained from the Protein Data Bank (http://www.pdb.org), chosen specifically for its high-resolution data, human origin, and established reliability in the literature. Protein refinement was executed via the Protein Preparation Wizard within the Schrödinger Suite 2020, utilizing the Maestro [30] interface. Concurrently, the LigPrep module [31] was employed for ligand preparation, ensuring accurate atom typing and protonation states. Following the addition of hydrogen atoms and the assignment of bond orders, the Glide module [32] was used for grid generation. Finally, docking simulations were completed employing the Standard Precision (SP) mode.

#### 3.3.2. Molecular Dynamics Simulation

To evaluate the structural stability and temporal behavior of compound **5c** within the aromatase active site, molecular dynamics (MD) simulations were conducted [33]. These computational analyses were executed through the same software interfaces as described in our previous methodologies [28,34], ensuring a consistent approach to modeling the ligand–receptor complex.

#### 3.3.3. ADME Studies

The physicochemical, pharmacokinetic, and drug-likeness properties of the compounds, as well as the BOILED-Egg model, were calculated using the SwissAdme program (https://www.swissadme.ch/). Table 5 and Figure 5 were obtained using the SwissAdme program.

## 4. Conclusions

The aromatase enzyme is one of the most important targets in the treatment of breast cancer. This is because the aromatase enzyme is one of the enzymes involved in estrogen biosynthesis, which leads to the growth of tumor tissue. Resistance to existing aromatase inhibitors limits their clinical use, increasing the need for new and effective aromatase inhibitors. In this study, some new thiadiazole (**4a**–**4j**) and triazole structures (**5a**–**5j**) were designed and synthesized based on the structures of anastrazole and letrozole. Their antiproliferative activities against MCF7 and NIH3T3 cells were investigated. The aromatase enzyme inhibitory activity of the compounds was tested. Among the triazole compounds, those bearing fluorine, chlorine, methyl, methoxy, and nitro substituents on the phenyl ring (**5b**, **5c**, **5e**, **5f** and **5g**) were found to exhibit the most promising antiproliferative and anti-aromatase activities within the series. In silico studies showed that compound **5c** formed stable interactions with the active site of the aromatase enzyme. Therefore, the chemical structure of compound **5c** was considered to be a guide for further research in the design of new anti-aromatase enzyme inhibitors for breast cancer treatment.

## Figures and Tables

**Table 1:** The IC_50_ values (μM) of compounds against MCF7 and NIH3T3 cell lines and aromatase enzyme *.

Compounds	MCF-7	NIH3T3	Selectivity Index	Aromatase Enzyme Inhibition
**4a**	66.325 ± 1.859	83.057 ± 3.025	1.252	-
**4b**	47.854 ± 2.012	>100	>2.090	-
**4c**	20.759 ± 1.021	>100	>4.817	-
**4d**	>100	>100	-	-
**4e**	30.250 ± 1.045	69.410 ± 2.108	2.295	-
**4f**	24.007 ± 1.006	>100	>4.165	-
**4g**	52.459 ± 1.367	84.306 ± 3.148	1.607	-
**4h**	>100	>100	-	-
**4i**	>100	>100	-	-
**4j**	>100	>100	-	-
**5a**	70.145 ± 2.859	>100	>1.426	-
**5b**	**9.623 ± 0.357**	>100	**>10.392**	2.224 ± 0.110
**5c**	**3.142 ± 0.139**	>100	**>31.827**	0.064 ± 0.003
**5d**	47.122 ± 1.856	84.325 ± 3.067	1.790	-
**5e**	**7.569 ± 0.248**	68.332 ± 2.956	**9.028**	0.097 ± 0.003
**5f**	**4.758 ± 0.196**	>100	**>21.017**	0.092 ± 0.004
**5g**	**10.415 ± 0.337**	84.126 ± 3.455	**8.077**	1.023 ± 0.050
**5h**	>100	>100	-	-
**5i**	>100	>100	-	-
**5j**	>100	>100	-	-
**Doxorubicin**	1.940 ± 0.084	>100	-	-
**Letrozole**	0.875 ± 0.038	>100	-	0.031 ± 0.001

* The test results were expressed as means of quartet assays.

**Table 2:** Interaction types for compounds **5c**, **5e** and **5f** on aromatase enzyme.

Comp.	Moiety	Pi-pi Interactions	Cation-pi Interactions	Salt Bridge	Hydrogen Bonds	Halogen Bonds
**5c**	**Phenyl**	HEM	-	-	-	-
		Phenyl ring of Phe134				
	**Triazole**	HEM	HEM	HEM	-	-
	**Triazole-N**	-	-	-	HEM	-
	**Sulphur**	-	-	HEM	-	-
	**4-CI-phenyl**	Indole ring of Trp224	-	-	-	Hydroxyl of Ser478
**5e**	**Phenyl**	HEM	-	-	-	-
		Phenyl ring of Phe134				
	**Triazole**	HEM	HEM	HEM	-	-
	**Triazole-N**	-	-	-	HEM	-
	**Sulphur**	-	-	HEM	-	-
**5f**	**Phenyl**	HEM	-	-	-	-
		Phenyl ring of Phe134				
	**Triazole**	HEM	HEM	HEM	-	-
	**Triazole-N**	-	-	-	HEM	-
	**Sulphur**	-	-	HEM	-	-
	**4-OCH3-phenyl**	Indole ring of Trp224	-	-	-	-

**Table 3:** RMSD, RMSF and Rg parameters (Å) for compound **5c** on aromatase enzyme.

Complex	RMSD	Rg	RMSF
**5c-3EQM**	2.4 Å	3.7–3.9 Å	Arg115 (0.64 Å), Phe116 (0.75 Å), Ile133 (0.55 Å), Phe134 (0.61 Å), Phe221 (0.89 Å), Trp224 (0.98 Å), Gln225 (1.09 Å), Leu228 (0.90 Å), Ile305 (0.93 Å), Ala306 (0.86 Å), Asp309 (0.92 Å), Thr310 (0.62 Å), Val369 (0.75 Å), Val370 (0.69 Å), Leu372 (1.01 Å), Val373 (0.93 Å), Met374 (0.83 Å), Arg375 (0.61 Å), Cys437 (0.58 Å), Leu477 (1.25 Å), Ser478 (1.09 Å), Leu479 (1.00 Å), His480 (0.89 Å)

**Table 4:** Interacting amino acids and fractions for compound **5c** on the aromatase enzyme.

Complex	Amino Acids Interacting Above 10%	Interaction Fractions
**5c-3EQM**	Arg115 (H-bond: 33%) Phe116 (Water mediated H-bond: 16%) Trp224 (π-π interaction: 37%) Trp224 (Cation-π interaction: 11%) Asp309 (Water mediated H-bond: 41%) Met374 (H-bond: 45%) Leu479 (Water mediated H-bond: 10%) HEM (Metal coordination 13% and 22%)	***Water mediated H-bond (blue)*** Phe116, Gln225, Ala306, Asp309, Thr310, Val369, Leu372, Met374, Leu477, Ser478, Leu479, His480
		***H-bond (green)*** Arg115, Phe116, Gln225, Ile305, Thr310, Met374, Arg375, Ser478
		***Ionic interaction (pink)*** Asp309, Thr310, Val369, Cys437, Ser478, His480
		***Hydrophobic interaction (purple)*** Ile133, Phe134, Phe221, Trp224, Leu228, Ile305, Val370, Leu372, Val373, Met374, Leu477, His480

**Table 5:** The physicochemical, pharmacokinetic and drug-likeness properties of all compounds.

Comp.	MW	*n*-ROTB	HBA	HBD	TPSA	Log *P*	Log *S*	Lipinski	Ghose	Veber	Egan	Muegge
**4a**	292.36	4	3	1	89.84	2.55	−4.23	Yes	Yes	Yes	Yes	Yes
**4b**	310.35	4	4	1	89.84	2.94	−4.38	Yes	Yes	Yes	Yes	Yes
**4c**	326.80	4	3	1	89.84	3.06	−4.81	Yes	Yes	Yes	Yes	Yes
**4d**	371.25	4	3	1	89.84	3.18	−5.13	Yes	Yes	Yes	Yes	Yes
**4e**	306.38	4	3	1	89.84	2.79	−4.52	Yes	Yes	Yes	Yes	Yes
**4f**	322.38	5	4	1	99.07	2.23	−4.28	Yes	Yes	Yes	Yes	Yes
**4g**	337.36	5	5	1	135.66	2.33	−4.26	Yes	Yes	Yes	No	Yes
**4h**	230.29	3	3	1	89.84	1.19	−2.84	Yes	Yes	Yes	Yes	Yes
**4i**	244.32	4	3	1	89.84	1.47	−3.06	Yes	Yes	Yes	Yes	Yes
**4j**	298.41	4	3	1	89.84	2.51	−4.21	Yes	Yes	Yes	Yes	Yes
**5a**	292.36	3	2	1	89.49	2.61	−3.85	Yes	Yes	Yes	Yes	Yes
**5b**	310.35	3	3	1	89.49	2.60	−4.00	Yes	Yes	Yes	Yes	Yes
**5c**	326.80	3	2	1	89.49	2.72	−4.43	Yes	Yes	Yes	Yes	Yes
**5d**	371.25	3	2	1	89.49	2.84	−4.75	Yes	Yes	Yes	Yes	Yes
**5e**	306.38	3	2	1	89.49	2.45	−4.14	Yes	Yes	Yes	Yes	Yes
**5f**	322.38	4	3	1	98.72	1.89	−3.90	Yes	Yes	Yes	Yes	Yes
**5g**	337.36	4	4	1	135.31	1.99	−3.88	Yes	Yes	Yes	No	Yes
**5h**	230.29	2	2	1	89.49	0.84	−2.46	Yes	Yes	Yes	Yes	Yes
**5i**	244.32	3	2	1	89.49	1.12	−2.68	Yes	Yes	Yes	Yes	Yes
**5j**	298.41	3	2	1	89.49	2.57	−3.83	Yes	Yes	Yes	Yes	Yes

MW: Molecular weight; n-ROTB: number of rotatable bonds; HBA: number of hydrogen bond acceptors; HBD: number of hydrogen bond donors; TPSA: topological polar surface area; Log *P*: calculated partition coefficient; log *S*: calculated water solubility.

## Data Availability

Data are contained within the article or Supplementary Materials.

## Supplementary Materials

The following supporting information can be downloaded at https://www.mdpi.com/article/10.3390/ph19020224/s1. Figure S1. The chemical structure of compound **3a**. Figure S2. IR spectrum for **3a**. Figure S3. ^1^H-NMR spectrum for **3a**. Figure S4. ^13^C-NMR spectrum for **3a**. Figure S5. The chemical structure of compound **3b**. Figure S6. IR spectrum for **3b**. Figure S7. ^1^H-NMR spectrum for **3b**. Figure S8. ^13^C-NMR spectrum for **3b**. Figure S9. The chemical structure of compound **3c**. Figure S10. IR spectrum for **3c**. Figure S11. ^1^H-NMR spectrum for **3c**. Figure S12. ^13^C-NMR spectrum for **3c**. Figure S13. The chemical structure of compound **3d**. Figure S14. IR spectrum for **3d**. Figure S15. ^1^H-NMR spectrum for **3d**. Figure S16. ^13^C-NMR spectrum for **3d**. Figure S17. The chemical structure of compound **3e**. Figure S18. IR spectrum for **3e**. Figure S19. ^1^H-NMR spectrum for **3e**. Figure S20. ^13^C-NMR spectrum for **3e**. Figure S21. The chemical structure of compound **3f**. Figure S22. IR spectrum for **3f**. Figure S23. ^1^H-NMR spectrum for **3f**. Figure S24. ^13^C-NMR spectrum for **3f**. Figure S25. The chemical structure of compound **3g**. Figure S26. IR spectrum for **3g**. Figure S27. ^1^H-NMR spectrum for **3g**. Figure S28. ^13^C-NMR spectrum for **3g**. Figure S29. The chemical structure of compound **3h**. Figure S30. IR spectrum for **3h**. Figure S31. ^1^H-NMR spectrum for **3h**. Figure S32. ^13^C-NMR spectrum for **3h**. Figure S33. The chemical structure of compound **3i**. Figure S34. IR spectrum for **3i**. Figure S35. ^1^H-NMR spectrum for **3i**. Figure S36. ^13^C-NMR spectrum for **3i**. Figure S37. The chemical structure of compound **3j**. Figure S38. IR spectrum for **3j**. Figure S39. ^1^H-NMR spectrum for **3j**. Figure S40. ^13^C-NMR spectrum for **3j**. Figure S41. The chemical structure of compound **4a**. Figure S42. IR spectrum for **4a**. Figure S43. ^1^H-NMR spectrum for **4a**. Figure S44. ^13^C-NMR spectrum for **4a**. Figure S45. The chemical structure of compound **4b**. Figure S46. IR spectrum for **4b**. Figure S47. ^1^H-NMR spectrum for **4b**. Figure S48. ^13^C-NMR spectrum for **4b**. Figure S49. The chemical structure of compound **4c**. Figure S50. IR spectrum for **4c**. Figure S51. ^1^H-NMR spectrum for **4c**. Figure S52. ^13^C-NMR spectrum for **4c**. Figure S53. The chemical structure of compound **4d**. Figure S54. IR spectrum for **4d**. Figure S55. ^1^H-NMR spectrum for **4d**. Figure S56. ^13^C-NMR spectrum for **4d**. Figure S57. The chemical structure of compound **4e**. Figure S58. IR spectrum for **4e**. Figure S59. ^1^H-NMR spectrum for **4e**. Figure S60. ^13^C-NMR spectrum for **4e**. Figure S61. The chemical structure of compound **4f**. Figure S62. IR spectrum for **4f**. Figure S63. ^1^H-NMR spectrum for **4f**. Figure S64. ^13^C-NMR spectrum for **4f**. Figure S65. The chemical structure of compound **4g**. Figure S66. IR spectrum for **4g**. Figure S67. ^1^H-NMR spectrum for **4g**. Figure S68. ^13^C-NMR spectrum for **4g**. Figure S69. The chemical structure of compound **4h**. Figure S70. IR spectrum for **4h**. Figure S71. ^1^H-NMR spectrum for **4h**. Figure S72. ^13^C-NMR spectrum for **4h**. Figure S73. The chemical structure of compound **4i**. Figure S74. IR spectrum for **4i**. Figure S75. ^1^H-NMR spectrum for **4i**. Figure S76. ^13^C-NMR spectrum for **4i**. Figure S77. The chemical structure of compound **4j**. Figure S78. IR spectrum for **4j**. Figure S79. ^1^H-NMR spectrum for **4j**. Figure S80. ^13^C-NMR spectrum for **4j**. Figure S81. The chemical structure of compound **5a**. Figure S82. IR spectrum for **5a**. Figure S83. ^1^H-NMR spectrum for **5a**. Figure S84. ^13^C-NMR spectrum for **5a**. Figure S85. The chemical structure of compound **5b**. Figure S86. IR spectrum for **5b**. Figure S87. ^1^H-NMR spectrum for **5b**. Figure S88. ^13^C-NMR spectrum for **5b**. Figure S89. The chemical structure of compound **5c**. Figure S90. IR spectrum for **5c**. Figure S91. ^1^H-NMR spectrum for **5c**. Figure S92. ^13^C-NMR spectrum for **5c**. Figure S93. The chemical structure of compound **5d**. Figure S94. IR spectrum for **5d**. Figure S95. ^1^H-NMR spectrum for **5d**. Figure S96. ^13^C-NMR spectrum for **5d**. Figure S97. The chemical structure of compound **5e**. Figure S98. IR spectrum for **5e**. Figure S99. ^1^H-NMR spectrum for **5e**. Figure S100. ^13^C-NMR spectrum for **5e**. Figure S101. The chemical structure of compound **5f**. Figure S102. IR spectrum for **5f**. Figure S103. ^1^H-NMR spectrum for **5f**. Figure S104. ^13^C-NMR spectrum for **5f**. Figure S105. The chemical structure of compound **5g**. Figure S106. IR spectrum for **5g**. Figure S107. ^1^H-NMR spectrum for **5g**. Figure S108. ^13^C-NMR spectrum for **5g**. Figure S109. The chemical structure of compound **5h**. Figure S110. IR spectrum for **5h**. Figure S111. ^1^H-NMR spectrum for **5h**. Figure S112. ^13^C-NMR spectrum for **5h**. Figure S113. The chemical structure of compound **5i**. Figure S114. IR spectrum for **5i**. Figure S115. ^1^H-NMR spectrum for **5i**. Figure S116. ^13^C-NMR spectrum for **5i**. Figure S117. The chemical structure of compound **5j**. Figure S118. IR spectrum for **5j**. Figure S119. ^1^H-NMR spectrum for **5j**. Figure S120. ^13^C-NMR spectrum for **5j**. Figure S121. (A) Overlay of the binding poses of compounds **5c**, **5e** and **5f** with the aromatase enzyme. Two-dimensional and three-dimensional interaction profiles of derivatives **5c** (A), **5e** (B), and **5f** (C) with the active site of aromatase (PDB ID: 3EQM).

## Abbreviations

The following abbreviations are used in this manuscript:

MCF-7	Human breast adenocarcinoma cell
NIH3T3	Mouse embryonic fibroblast cell
MD	Molecular dynamics
ADME	Absorption, distribution, metabolism and excretion
RMSD	Root mean square deviation
RMSF	Root mean square fluctuation
SASA	Solvent accessible surface area
PSA	Polar surface area
TLC	Thin-layer chromatography
