# Supplementary material for: Design, Synthesis, Biological Evaluation and Molecular Docking Studies of New N-Heterocyclic Compounds as Aromatase Inhibitors
Source: Pharmaceuticals (Basel). 2026 Jan 27;19(2):224. doi: 10.3390/ph19020224 (PMC12943600; doi:10.3390/ph19020224)
Supplement: Supplementary file 1 [file pharmaceuticals-19-00224-s001.zip › pharmaceuticals-4038782-supplementary.pdf]

Design, Synthesis, Biological Evaluation and Molecular Docking Studies of New *N*-Heterocyclic Compounds as Aromatase Inhibitors

Fatih Tok [1], Begüm Nurpelin Sağlık Özkan [2,3,*], Yusuf Özkay [2,3] and Zafer Asım Kaplancıklı [2]

[1] Department of Pharmaceutical Chemistry, Faculty of Pharmacy, Marmara University, 34854 İstanbul, Türkiye; fatih.tok@marmara.edu.tr
[2] Department of Pharmaceutical Chemistry, Faculty of Pharmacy, Anadolu University, 26470 Eskişehir, Türkiye; yozkay@anadolu.edu.tr (Y.Ö.); zakaplan@anadolu.edu.tr (Z.A.K.)
[3] Central Research Laboratory (MERLAB), Faculty of Pharmacy, Anadolu University, 26470 Eskişehir, Türkiye
* Correspondence: bnsaglik@anadolu.edu.tr; Tel.: +90-335-058-0377

LIST OF CONTENTS

Figure S1. The chemical structure of compound **3a**
Figure S2. IR spectrum for **3a**
Figure S3. ^{1}H-NMR spectrum for **3a**
Figure S4. ^{13}C-NMR spectrum for **3a**
Figure S5. The chemical structure of compound **3b**
Figure S6. IR spectrum for **3b**
Figure S7. ^{1}H-NMR spectrum for **3b**
Figure S8. ^{13}C-NMR spectrum for **3b**
Figure S9. The chemical structure of compound **3c**
Figure S10. IR spectrum for **3c**
Figure S11. ^{1}H-NMR spectrum for **3c**
Figure S12. ^{13}C-NMR spectrum for **3c**
Figure S13. The chemical structure of compound **3d**
Figure S14. IR spectrum for **3d**
Figure S15. ^{1}H-NMR spectrum for **3d**
Figure S16. ^{13}C-NMR spectrum for **3d**
Figure S17. The chemical structure of compound **3e**
Figure S18. IR spectrum for **3e**
Figure S19. ^{1}H-NMR spectrum for **3e**
Figure S20. ^{13}C-NMR spectrum for **3e**
Figure S21. The chemical structure of compound **3f**
Figure S22. IR spectrum for **3f**
Figure S23. ^{1}H-NMR spectrum for **3f**
Figure S24. ^{13}C-NMR spectrum for **3f**
Figure S25. The chemical structure of compound **3g**
Figure S26. IR spectrum for **3g**
Figure S27. ^{1}H-NMR spectrum for **3g**
Figure S28. ^{13}C-NMR spectrum for **3g**
Figure S29. The chemical structure of compound **3h**
Figure S30. IR spectrum for **3h**
Figure S31. ^{1}H-NMR spectrum for **3h**
Figure S32. ^{13}C-NMR spectrum for **3h**
Figure S33. The chemical structure of compound **3i**
Figure S34. IR spectrum for **3i**
Figure S35. ^{1}H-NMR spectrum for **3i**
Figure S36. ^{13}C-NMR spectrum for **3i**
Figure S37. The chemical structure of compound **3j**
Figure S38. IR spectrum for **3j**

Figure S39. ^{1}H-NMR spectrum for **3j**
Figure S40. ^{13}C-NMR spectrum for **3j**
Figure S41. The chemical structure of compound **4a**
Figure S42. IR spectrum for **4a**
Figure S43. ^{1}H-NMR spectrum for **4a**
Figure S44. ^{13}C-NMR spectrum for **4a**
Figure S45. The chemical structure of compound **4b**
Figure S46. IR spectrum for **4b**
Figure S47. ^{1}H-NMR spectrum for **4b**
Figure S48. ^{13}C-NMR spectrum for **4b**
Figure S49. The chemical structure of compound **4c**
Figure S50. IR spectrum for **4c**
Figure S51. ^{1}H-NMR spectrum for **4c**
Figure S52. ^{13}C-NMR spectrum for **4c**
Figure S53. The chemical structure of compound **4d**
Figure S54. IR spectrum for **4d**
Figure S55. ^{1}H-NMR spectrum for **4d**
Figure S56. ^{13}C-NMR spectrum for **4d**
Figure S57. The chemical structure of compound **4e**
Figure S58. IR spectrum for **4e**
Figure S59. ^{1}H-NMR spectrum for **4e**
Figure S60. ^{13}C-NMR spectrum for **4e**
Figure S61. The chemical structure of compound **4f**
Figure S62. IR spectrum for **4f**
Figure S63. ^{1}H-NMR spectrum for **4f**
Figure S64. ^{13}C-NMR spectrum for **4f**
Figure S65. The chemical structure of compound **4g**
Figure S66. IR spectrum for **4g**
Figure S67. ^{1}H-NMR spectrum for **4g**
Figure S68. ^{13}C-NMR spectrum for **4g**
Figure S69. The chemical structure of compound **4h**
Figure S70. IR spectrum for **4h**
Figure S71. ^{1}H-NMR spectrum for **4h**
Figure S72. ^{13}C-NMR spectrum for **4h**
Figure S73. The chemical structure of compound **4i**
Figure S74. IR spectrum for **4i**
Figure S75. ^{1}H-NMR spectrum for **4i**
Figure S76. ^{13}C-NMR spectrum for **4i**
Figure S77. The chemical structure of compound **4j**
Figure S78. IR spectrum for **4j**
Figure S79. ^{1}H-NMR spectrum for **4j**
Figure S80. ^{13}C-NMR spectrum for **4j**
Figure S81. The chemical structure of compound **5a**
Figure S82. IR spectrum for **5a**
Figure S83. ^{1}H-NMR spectrum for **5a**
Figure S84. ^{13}C-NMR spectrum for **5a**
Figure S85. The chemical structure of compound **5b**
Figure S86. IR spectrum for **5b**
Figure S87. ^{1}H-NMR spectrum for **5b**
Figure S88. ^{13}C-NMR spectrum for **5b**
Figure S89. The chemical structure of compound **5c**
Figure S90. IR spectrum for **5c**
Figure S91. ^{1}H-NMR spectrum for **5c**
Figure S92. ^{13}C-NMR spectrum for **5c**
Figure S93. The chemical structure of compound **5d**

Figure S94. IR spectrum for **5d**
Figure S95. ^{1}H-NMR spectrum for **5d**
Figure S96. ^{13}C-NMR spectrum for **5d**
Figure S97. The chemical structure of compound **5e**
Figure S98. IR spectrum for **5e**
Figure S99. ^{1}H-NMR spectrum for **5e**
Figure S100. ^{13}C-NMR spectrum for **5e**
Figure S101. The chemical structure of compound **5f**
Figure S102. IR spectrum for **5f**
Figure S103. ^{1}H-NMR spectrum for **5f**
Figure S104. ^{13}C-NMR spectrum for **5f**
Figure S105. The chemical structure of compound **5g**
Figure S106. IR spectrum for **5g**
Figure S107. ^{1}H-NMR spectrum for **5g**
Figure S108. ^{13}C-NMR spectrum for **5g**
Figure S109. The chemical structure of compound **5h**
Figure S110. IR spectrum for **5h**
Figure S111. ^{1}H-NMR spectrum for **5h**
Figure S112. ^{13}C-NMR spectrum for **5h**
Figure S113. The chemical structure of compound **5i**
Figure S114. IR spectrum for **5i**
Figure S115. ^{1}H-NMR spectrum for **5i**
Figure S116. ^{13}C-NMR spectrum for **5i**
Figure S117. The chemical structure of compound **5j**
Figure S118. IR spectrum for **5j**
Figure S119. ^{1}H-NMR spectrum for **5j**
Figure S120. ^{13}C-NMR spectrum for **5j**
Figure S121. (**A**) Overlay of the binding poses of compounds **5c**, **5e** and **5f** with the aromatase enzyme. Two-dimensional and three-dimensional interaction profiles of derivatives **5c** (**A**), **5e** (**B**), and **5f** (**C**) with the active site of aromatase (PDB ID: 3EQM)

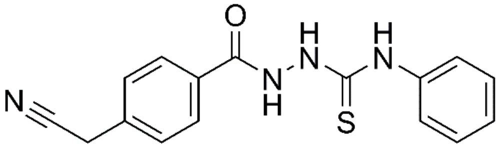

Figure S1. The chemical structure of compound **3a**

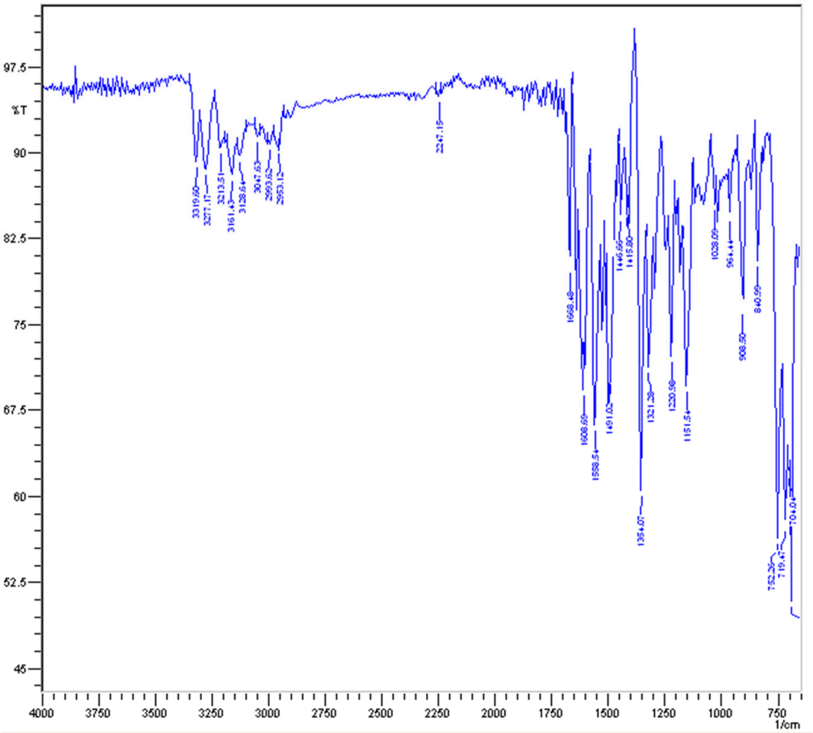

Figure S2. IR spectrum for **3a**

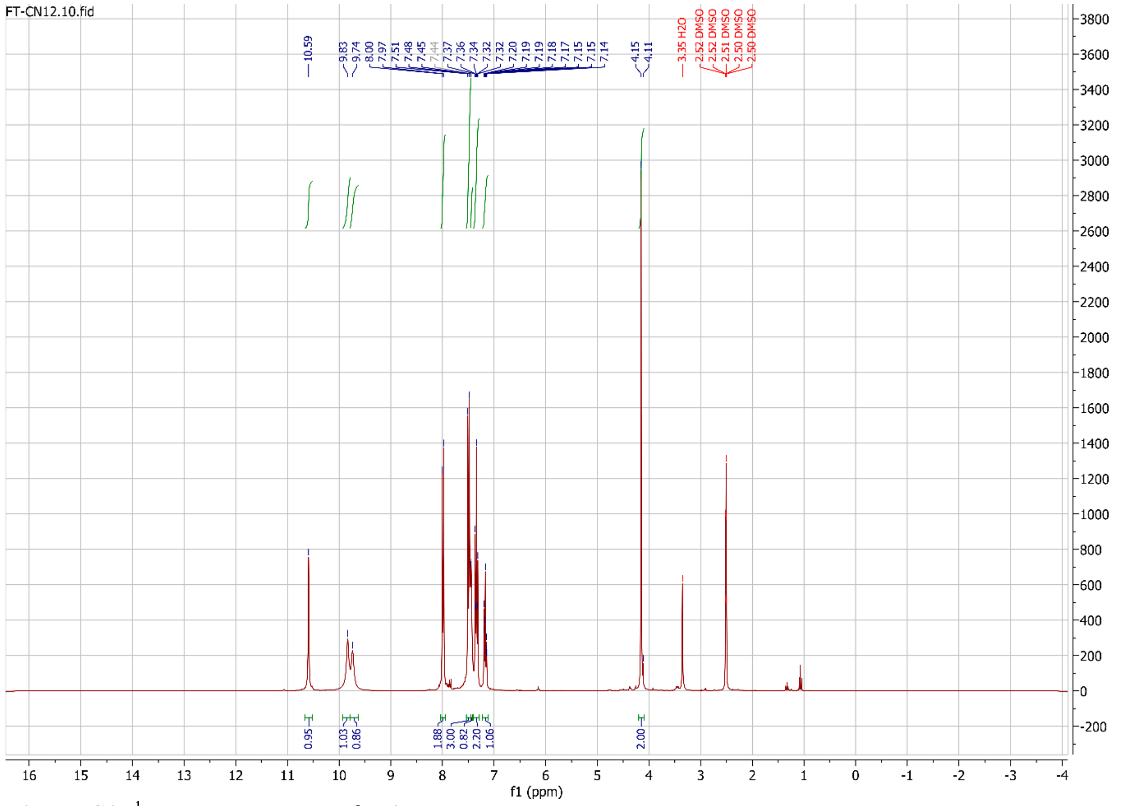

Figure S3. ^{1}H-NMR spectrum for **3a**

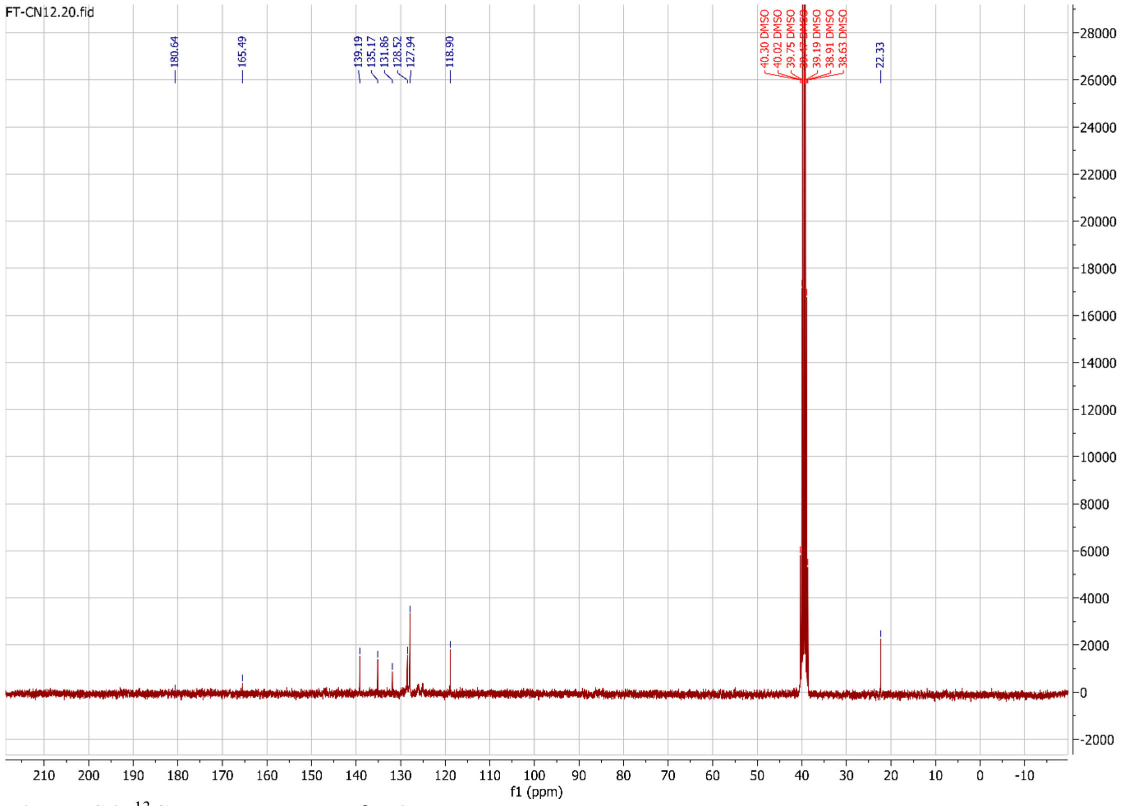

Figure S4. ^{13}C-NMR spectrum for **3a**

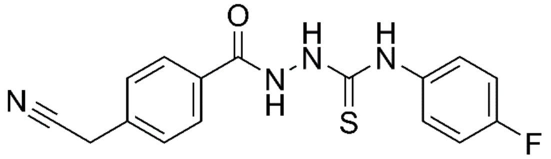

Figure S5. The chemical structure of compound **3b**

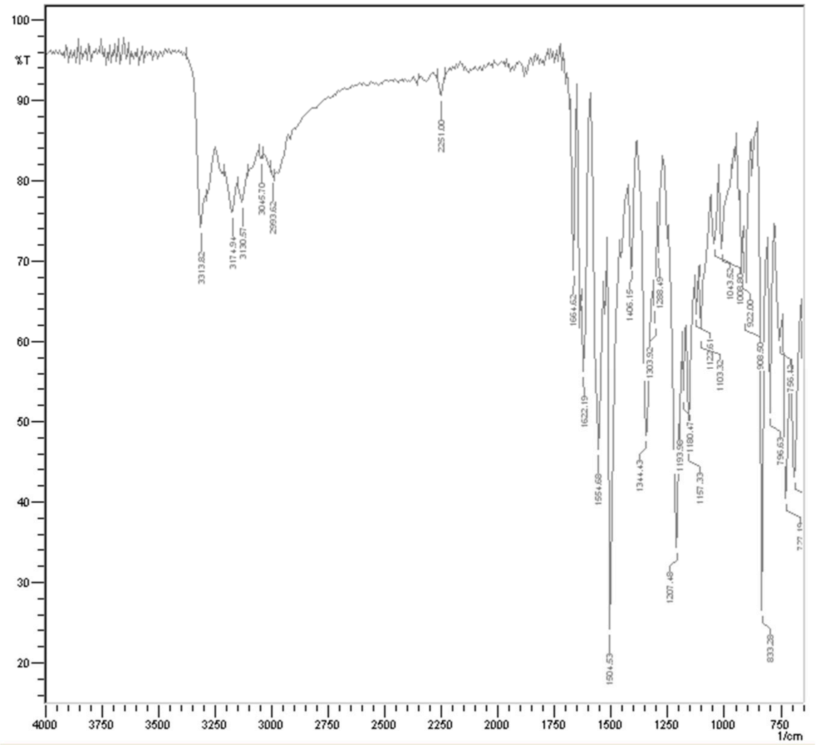

Figure S6. IR spectrum for **3b**

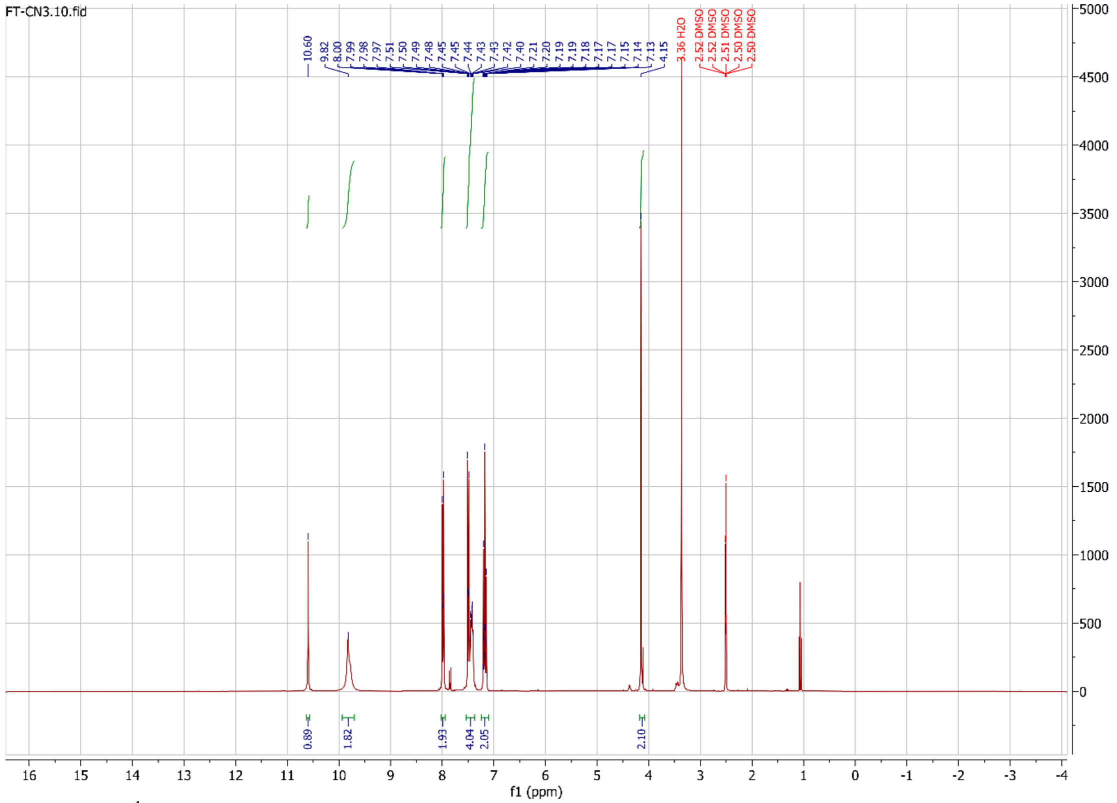

Figure S7. ^{1}H-NMR spectrum for **3b**

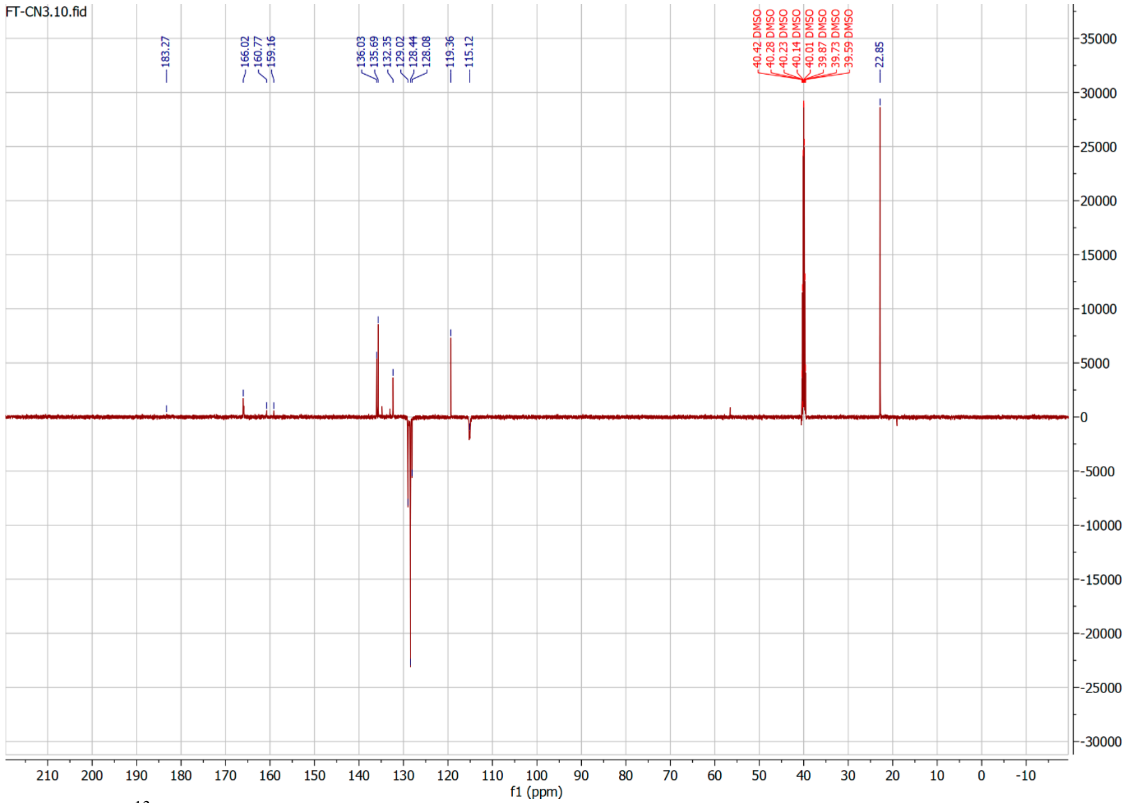

Figure S8. ^{13}C-NMR spectrum for **3b**

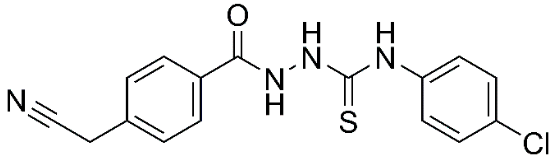

Figure S9. The chemical structure of compound **3c**

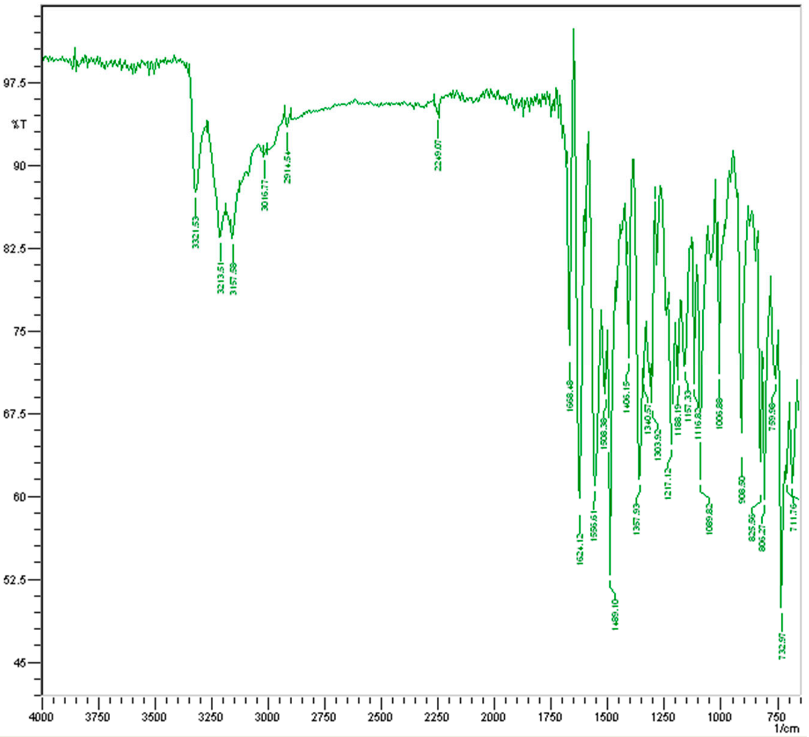

Figure S10. IR spectrum for **3c**

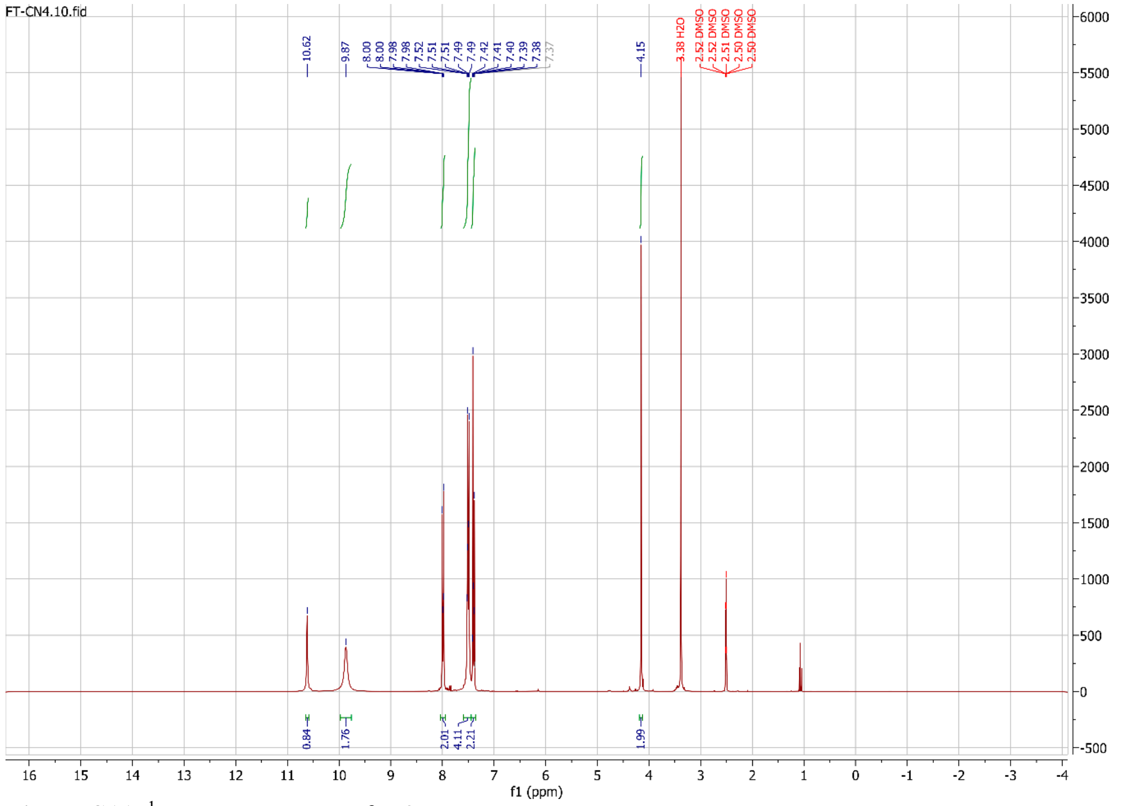

Figure S11. ^{1}H-NMR spectrum for **3c**

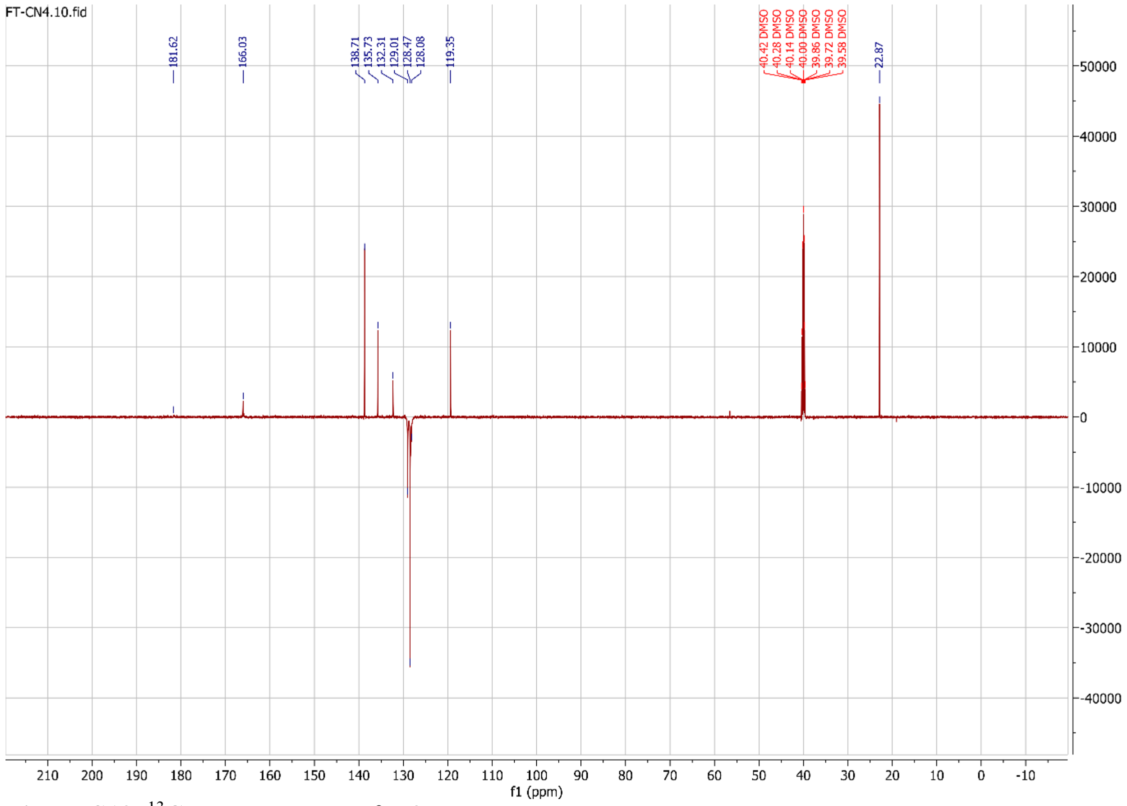

Figure S12. ^{13}C-NMR spectrum for **3c**

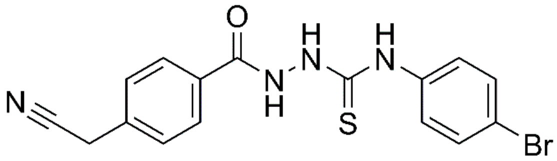

Figure S13. The chemical structure of compound **3d**

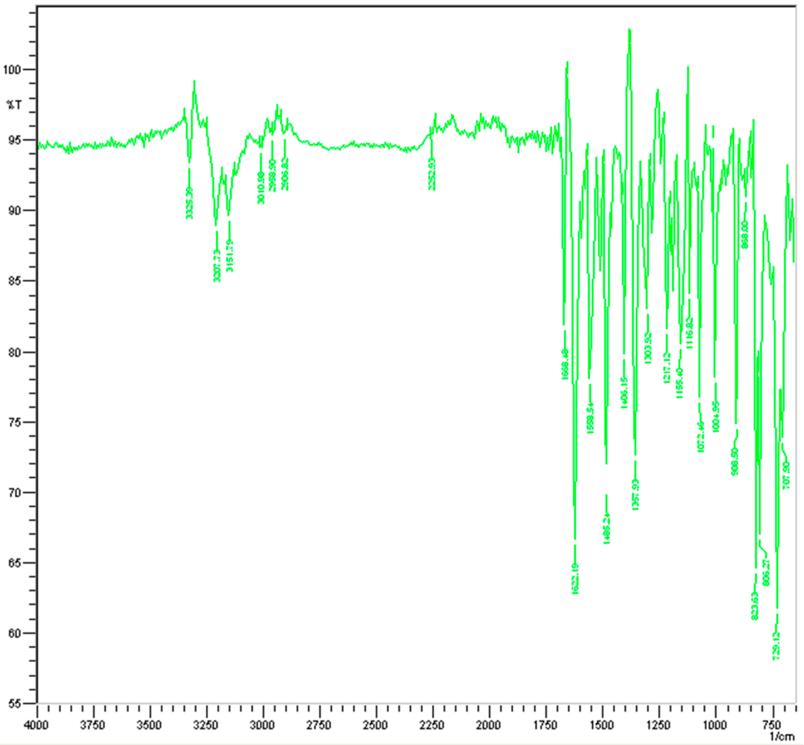

Figure S14. IR spectrum for **3d**

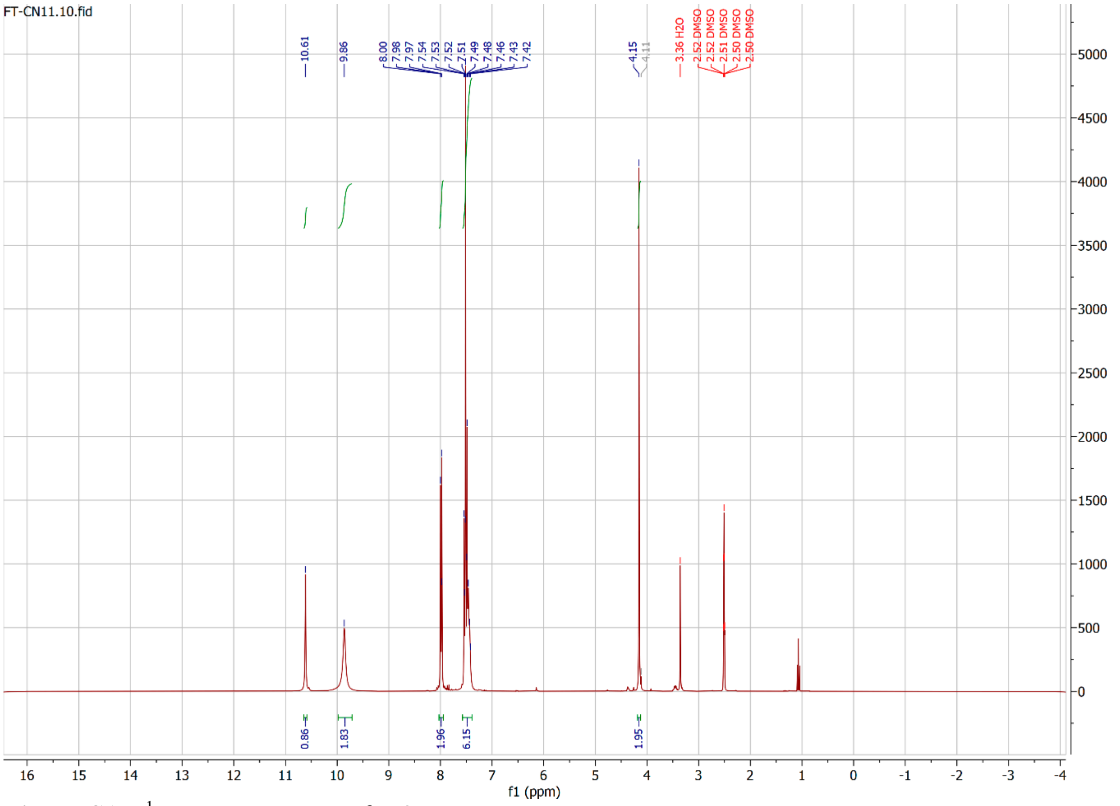

Figure S15. ^{1}H-NMR spectrum for **3d**

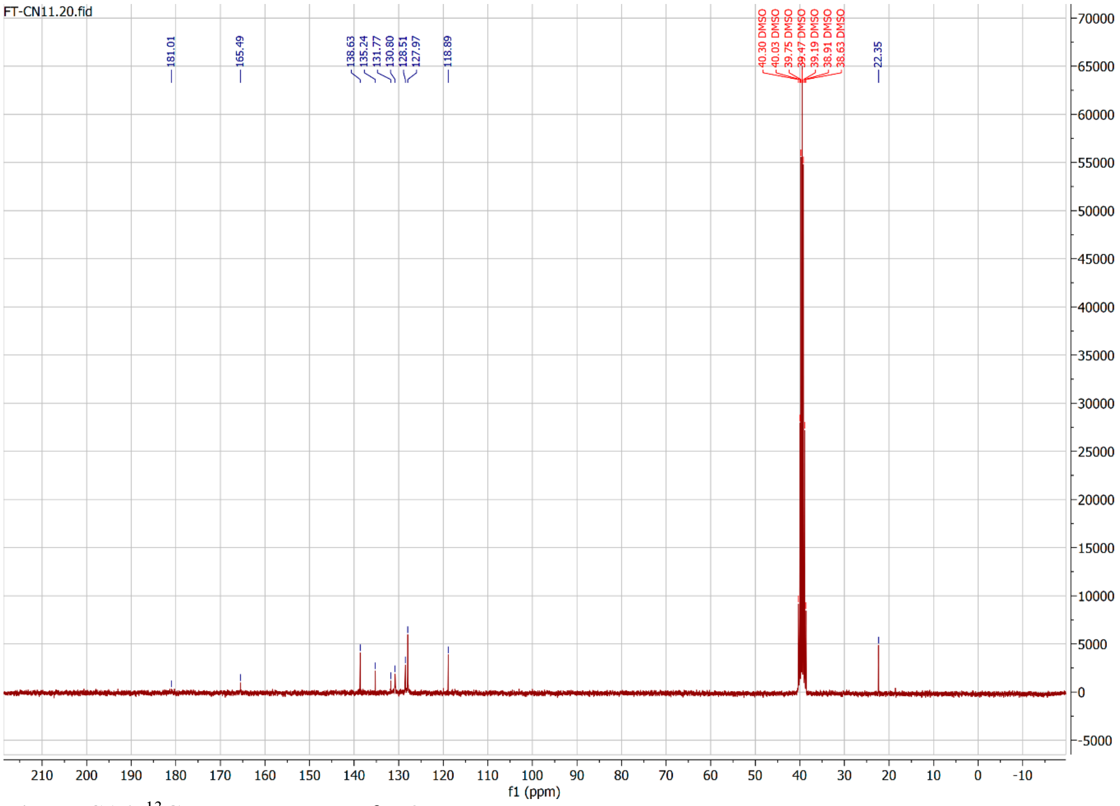

Figure S16. ^{13}C-NMR spectrum for **3d**

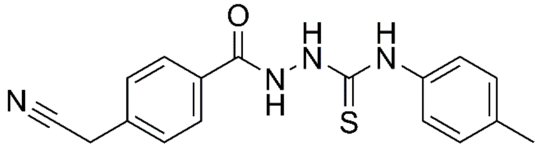

Figure S17. The chemical structure of compound **3e**

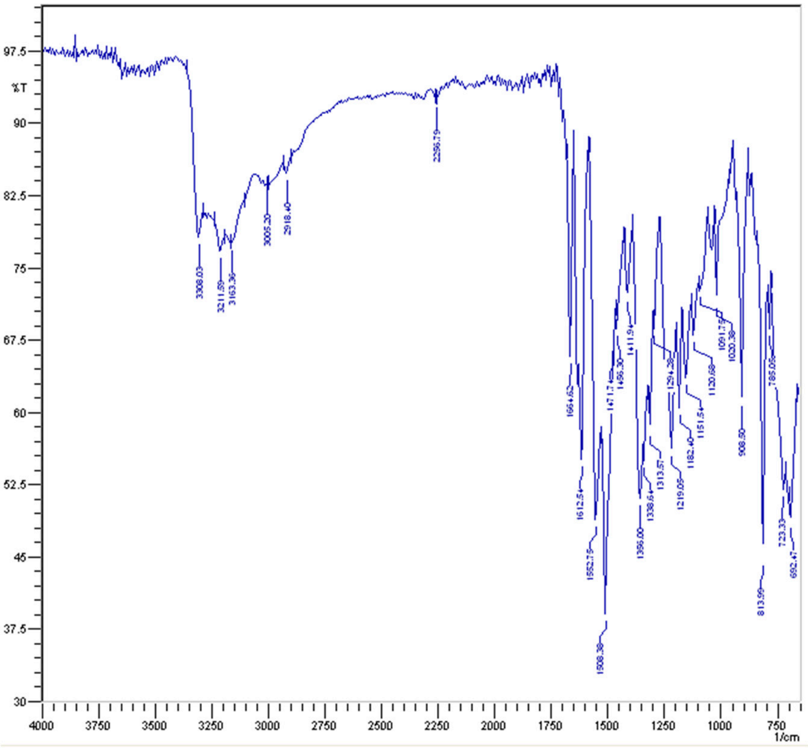

Figure S18. IR spectrum for **3e**

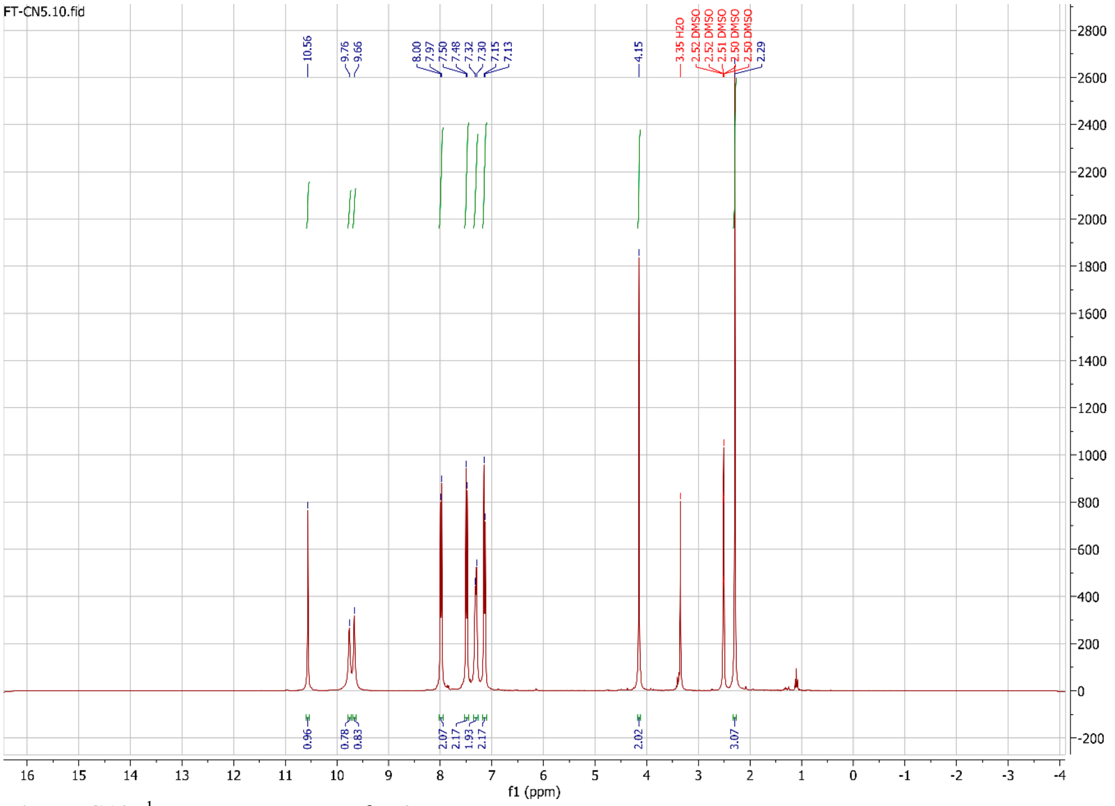

Figure S19. ^{1}H-NMR spectrum for **3e**

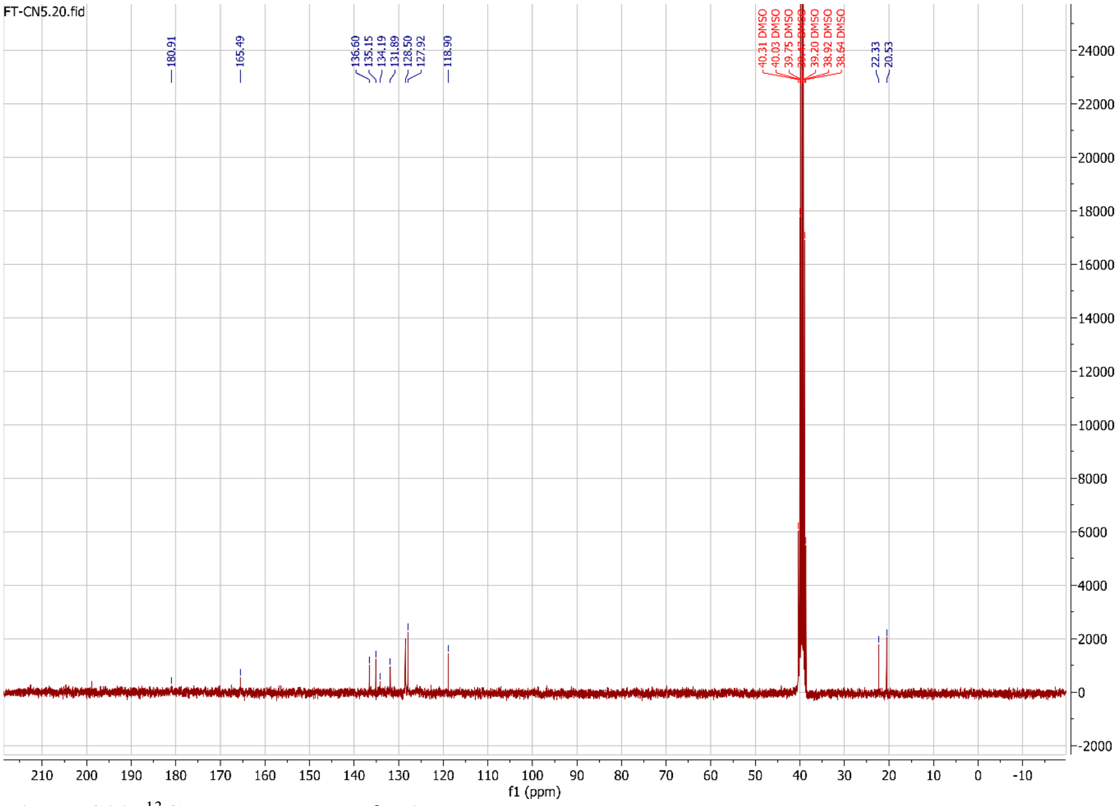

Figure S20. ^{13}C-NMR spectrum for **3e**

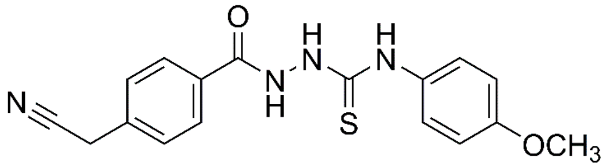

Figure S21. The chemical structure of compound **3f**

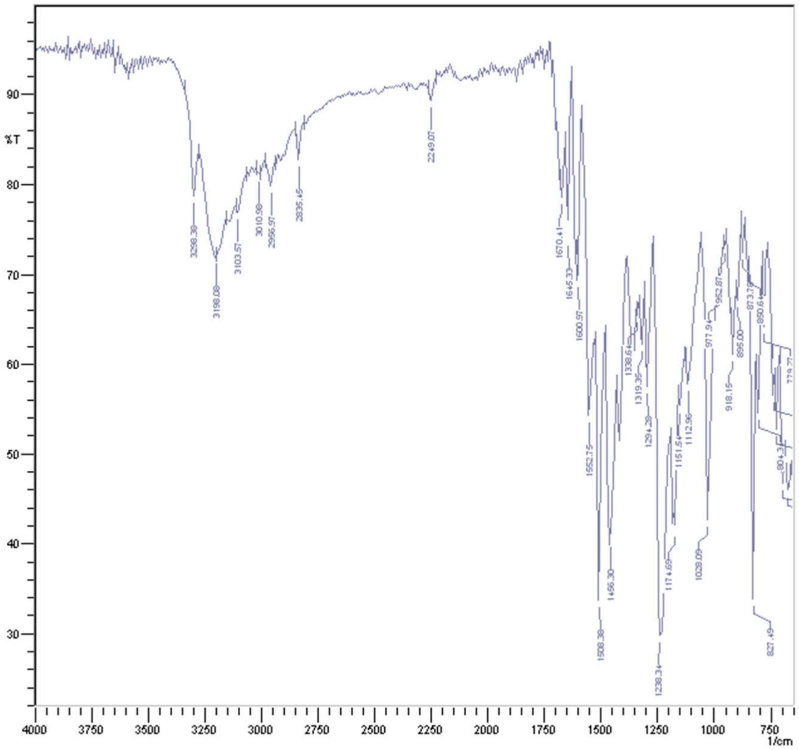

Figure S22. IR spectrum for **3f**

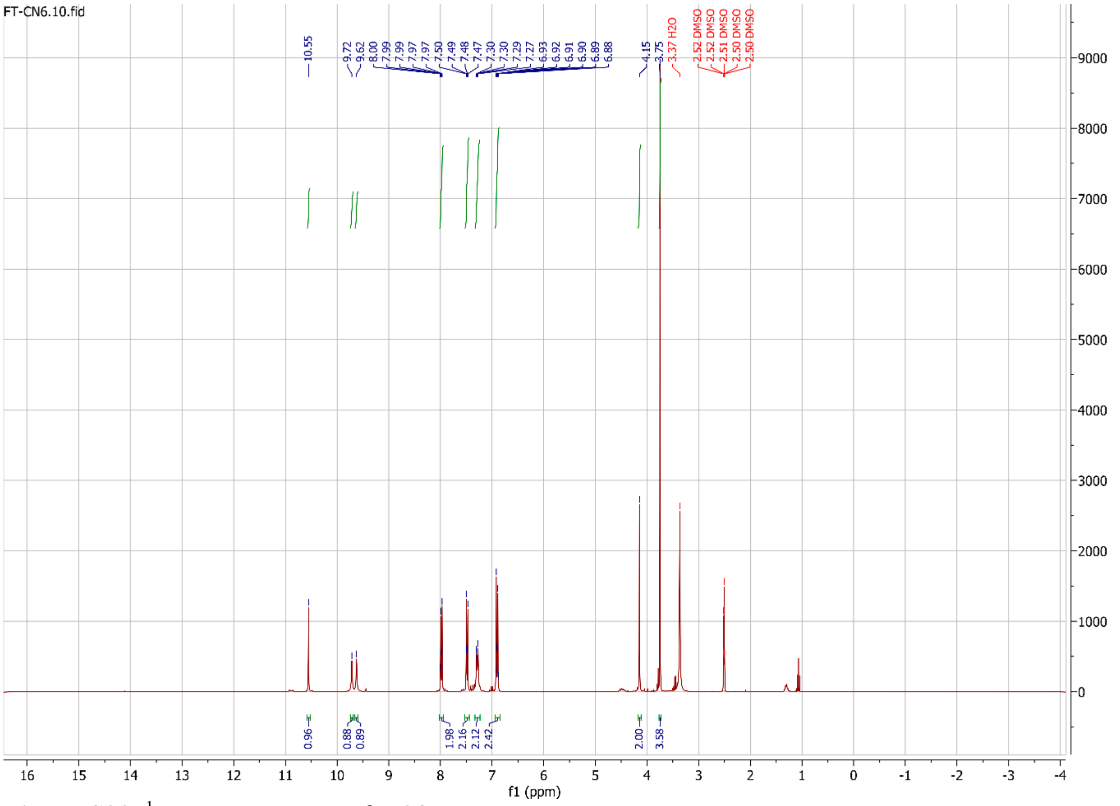

Figure S23. ^{1}H-NMR spectrum for **3f**

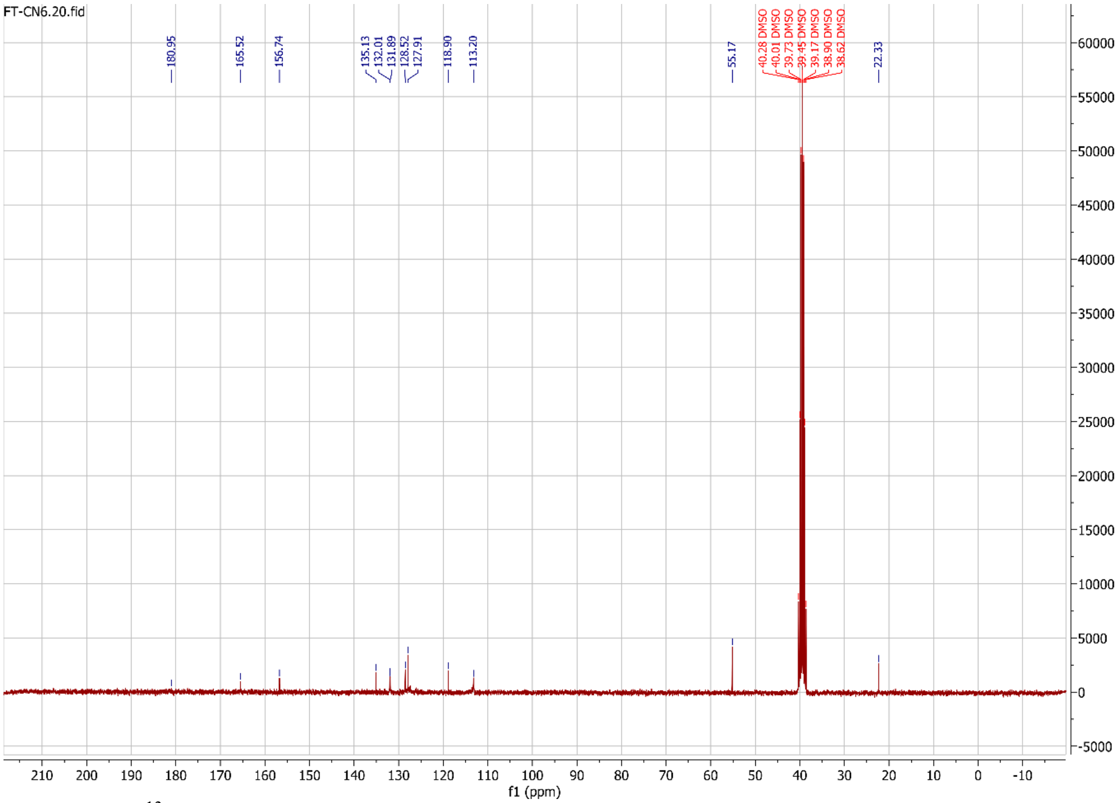

Figure S24. ^{13}C-NMR spectrum for **3f**

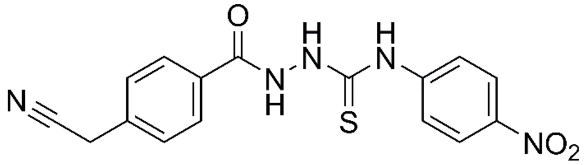

Figure S25. The chemical structure of compound **3g**

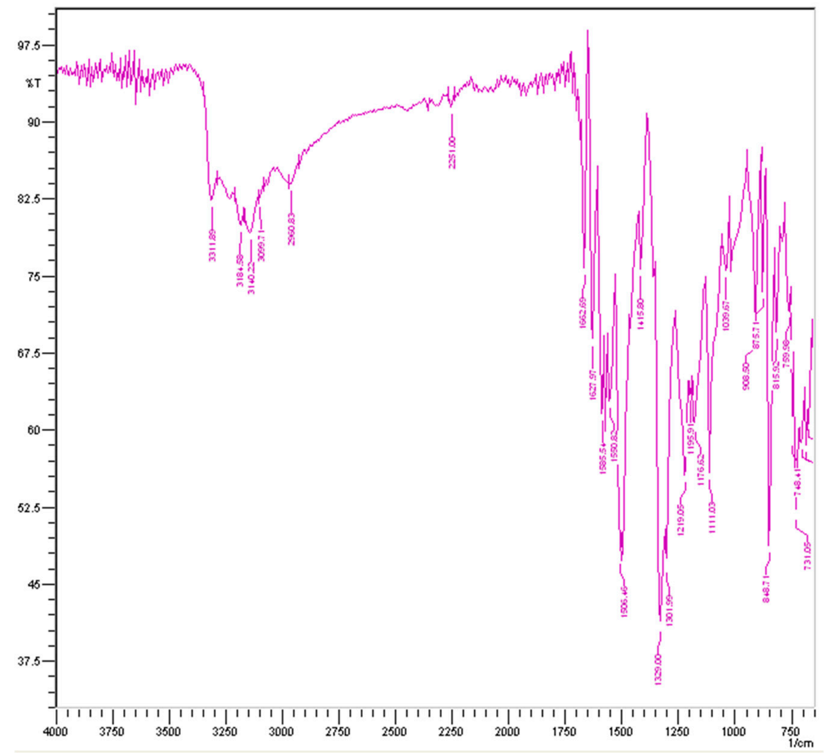

Figure S26. IR spectrum for **3g**

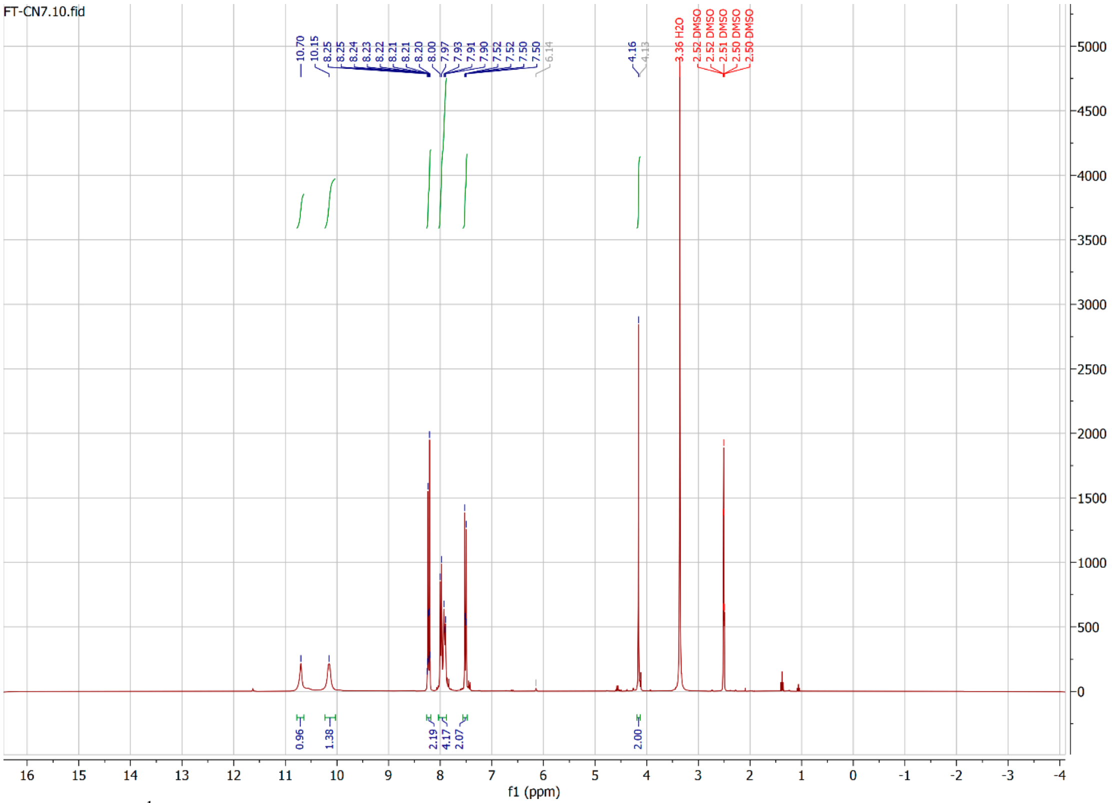

Figure S27. ^{1}H-NMR spectrum for **3g**

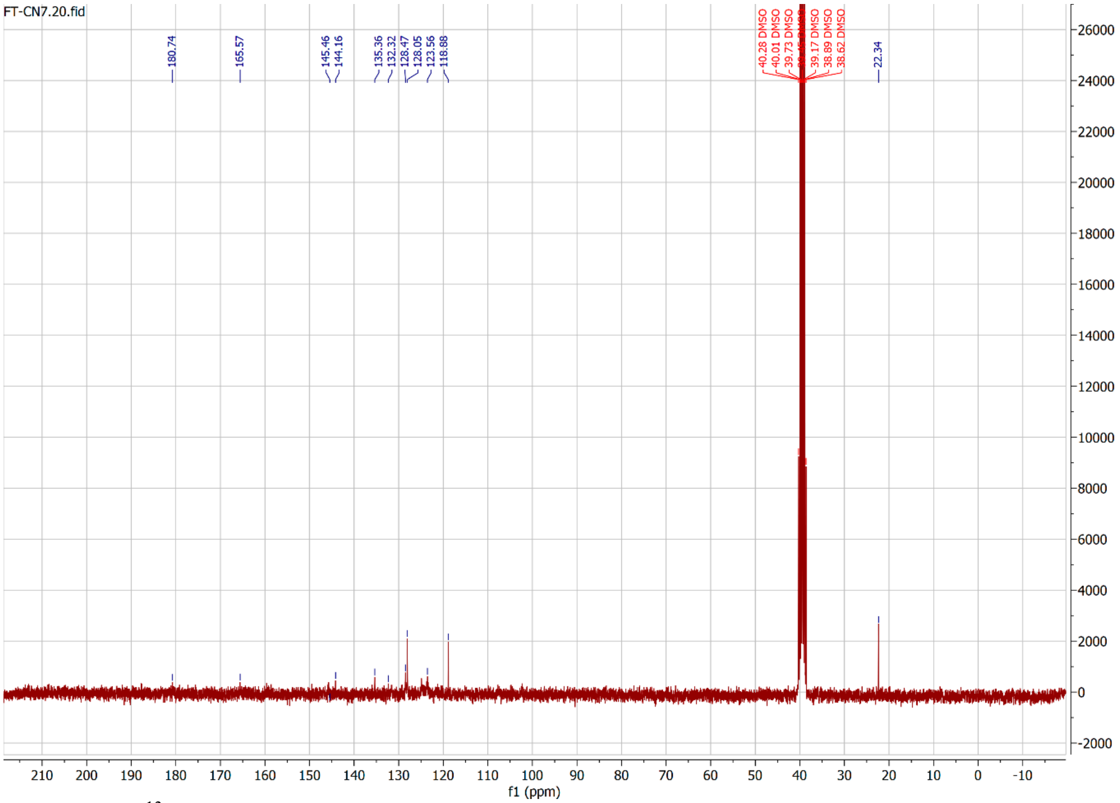

Figure S28. ^{13}C-NMR spectrum for **3g**

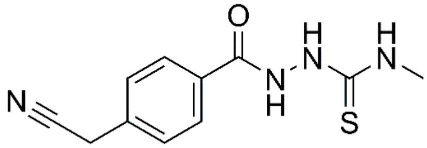

Figure S29. The chemical structure of compound **3h**

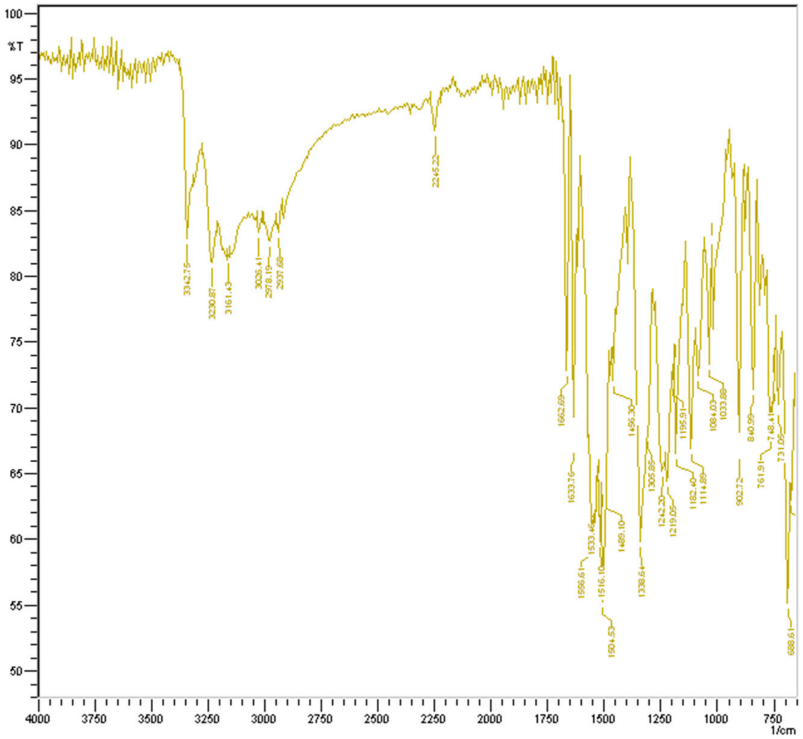

Figure S30. IR spectrum for **3h**

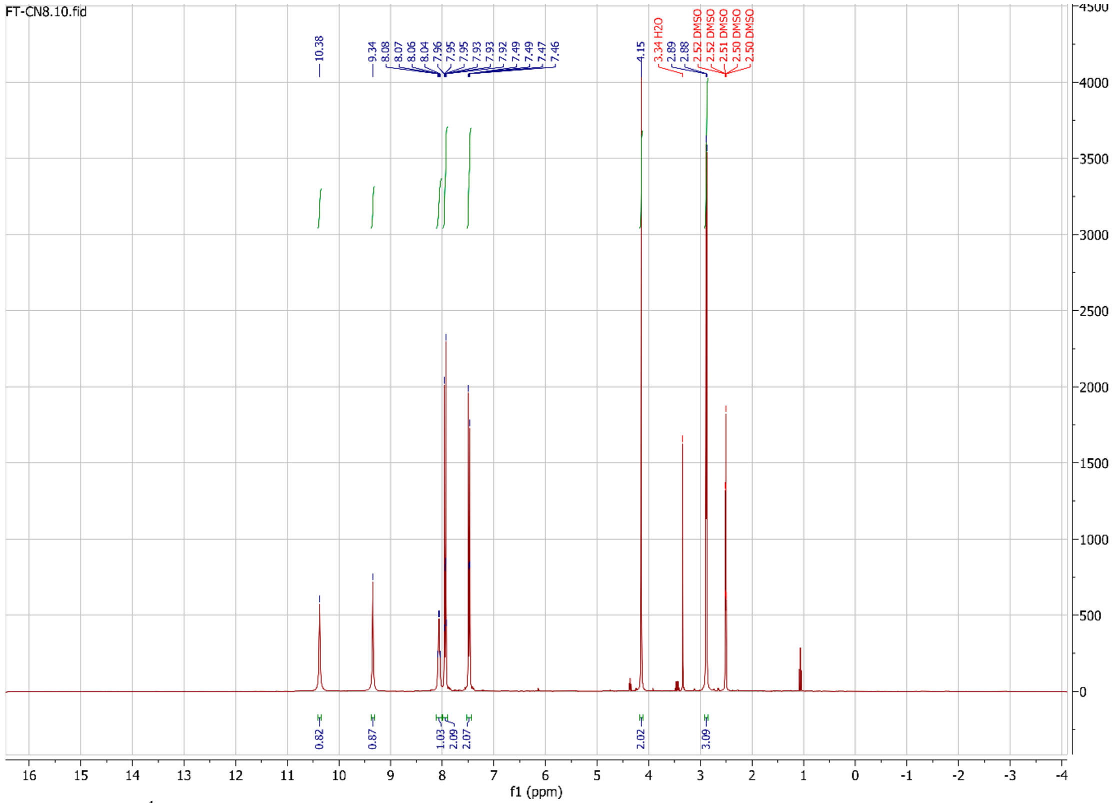

Figure S31. ^{1}H-NMR spectrum for **3h**

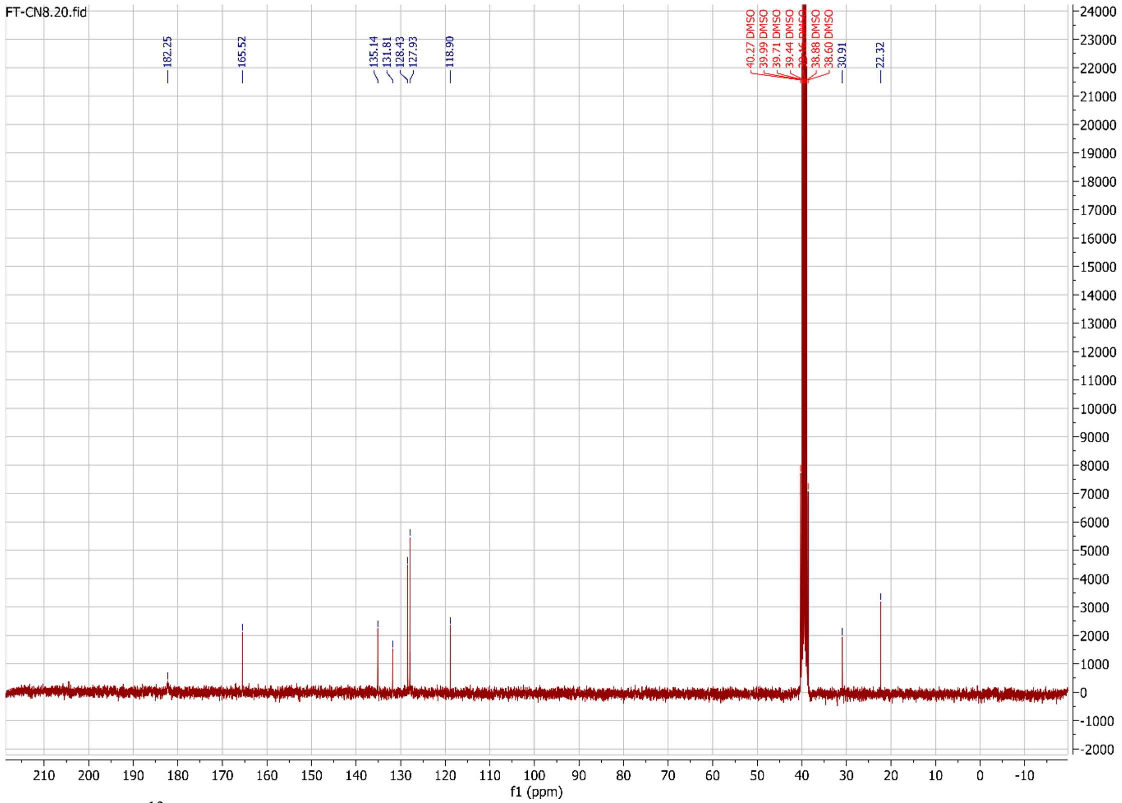

Figure S32. ^{13}C-NMR spectrum for **3h**

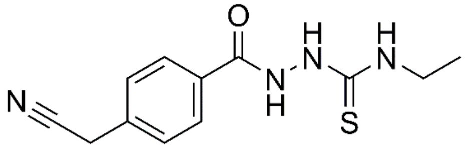

Figure S33. The chemical structure of compound **3i**

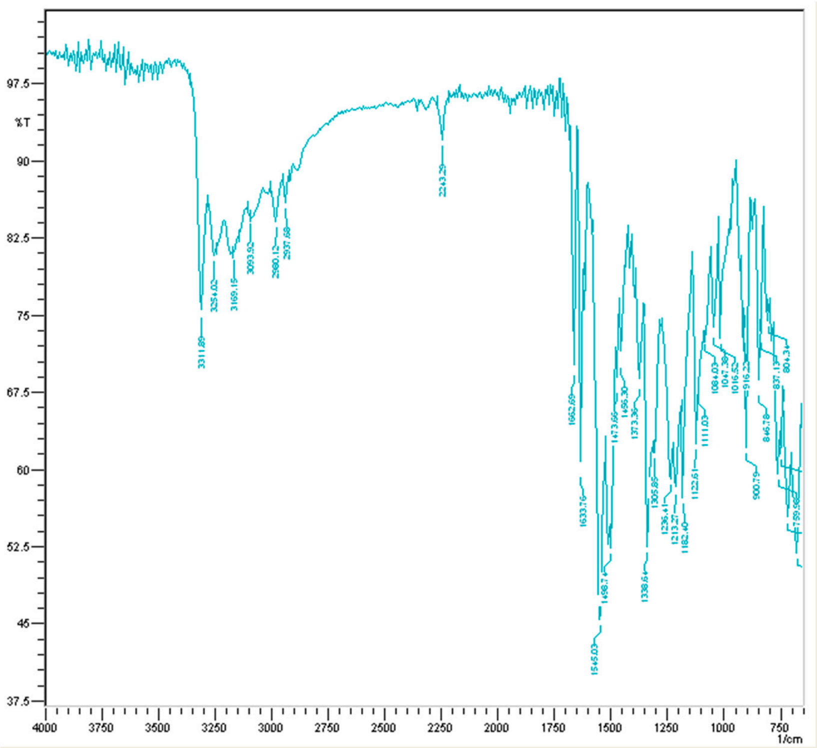

Figure S34. IR spectrum for **3i**

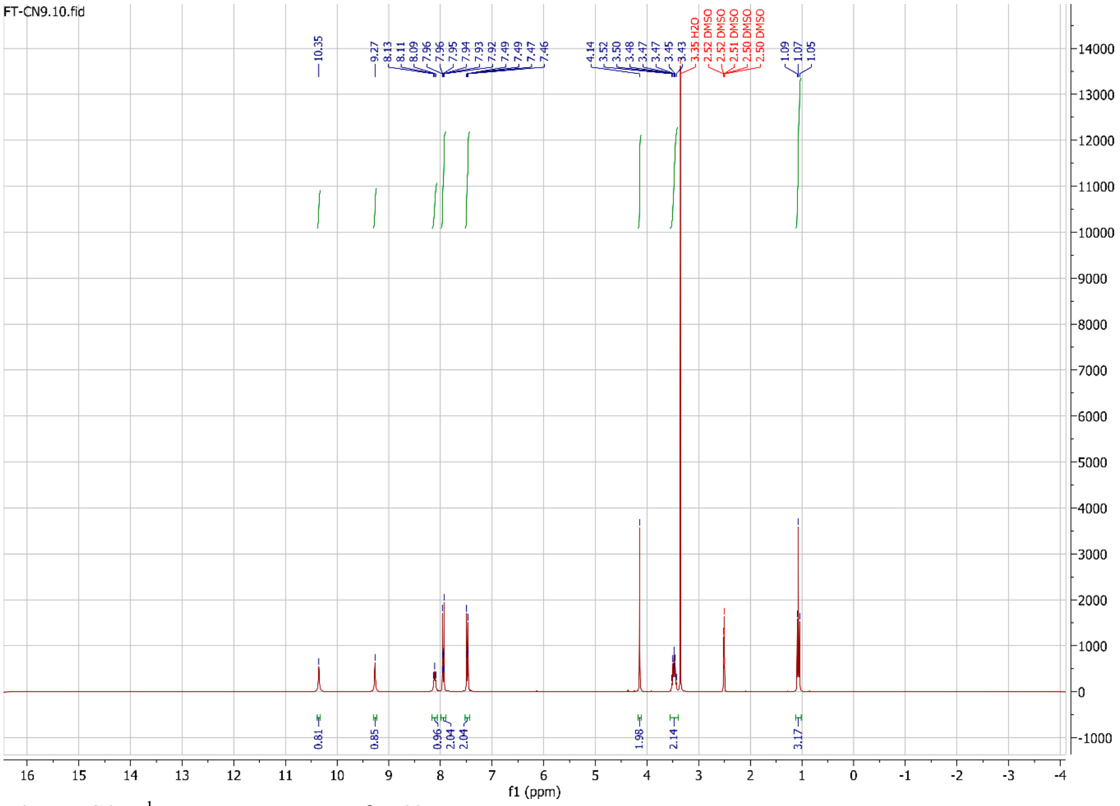

Figure S35. ^{1}H-NMR spectrum for **3i**

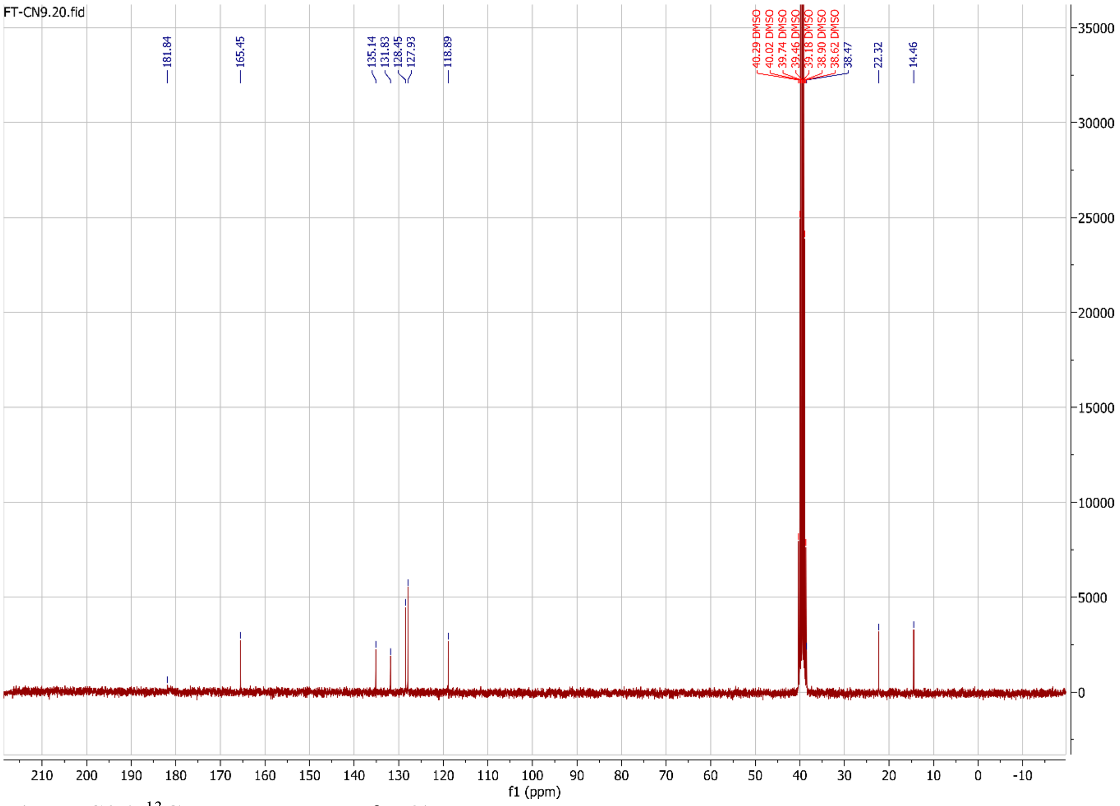

Figure S36. ^{13}C-NMR spectrum for **3i**

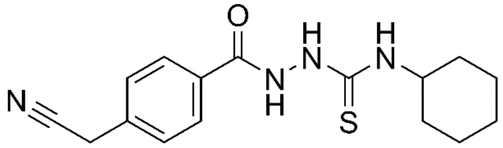

Figure S37. The chemical structure of compound **3j**

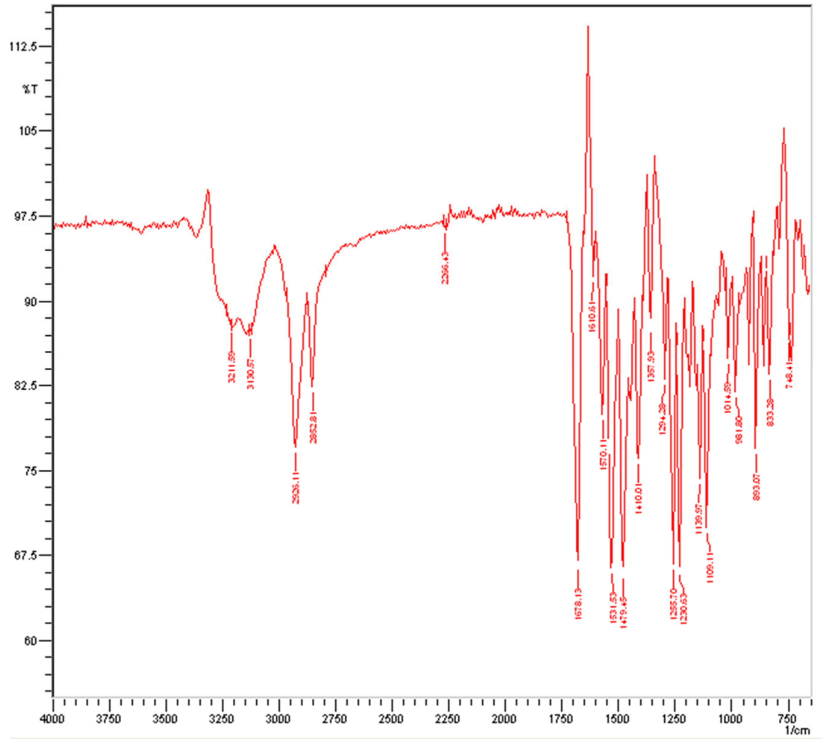

Figure S38. IR spectrum for **3j**

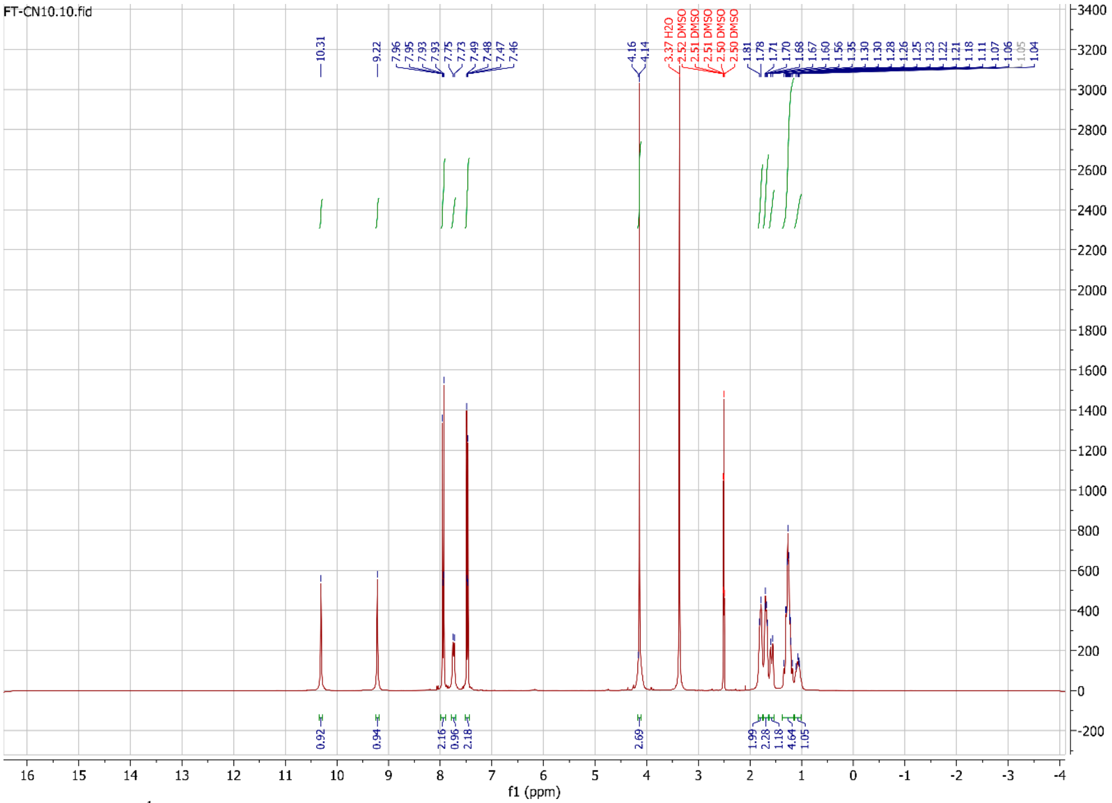

Figure S39. ^{1}H-NMR spectrum for **3j**

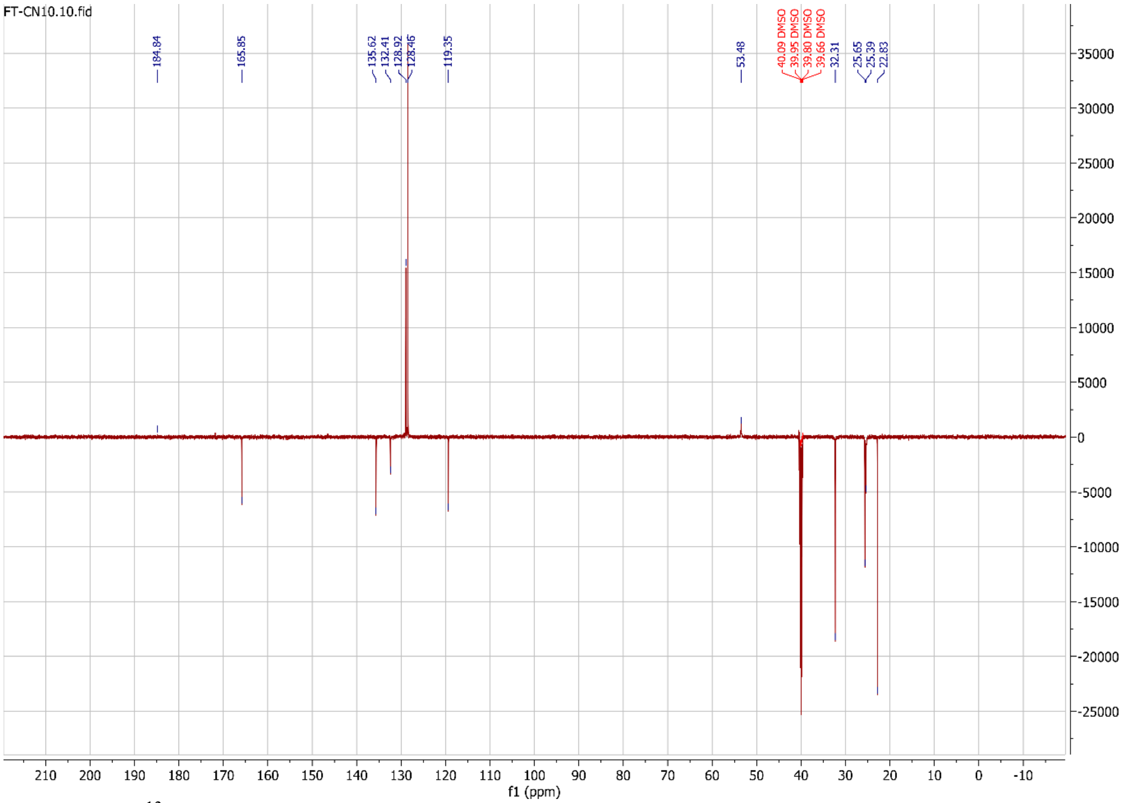

Figure S40. ^{13}C-NMR spectrum for **3j**

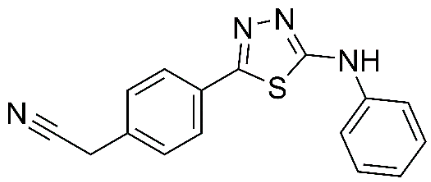

Figure S41. The chemical structure of compound **4a**

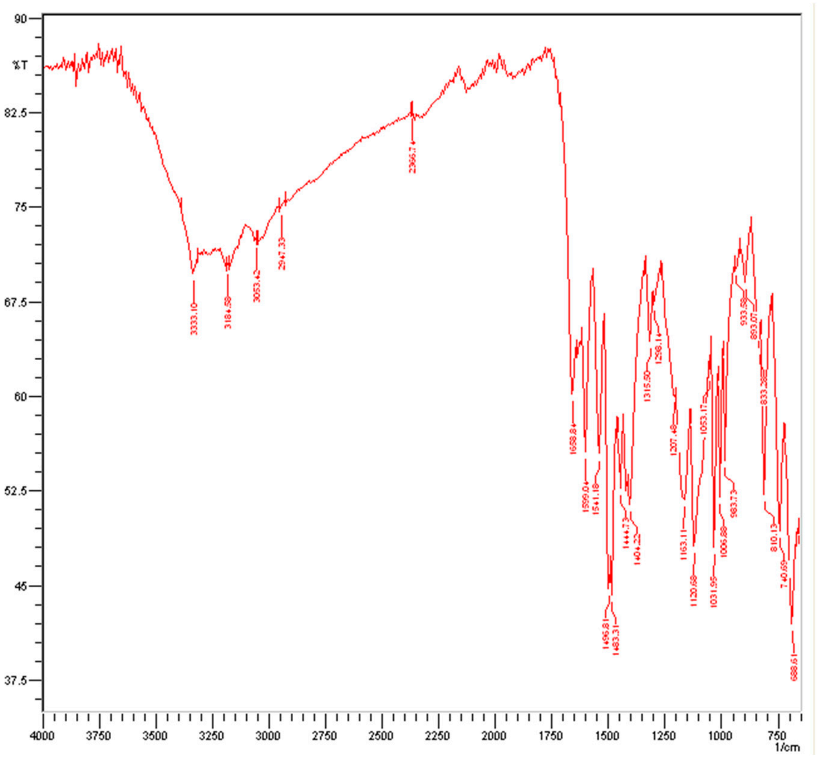

Figure S42. IR spectrum for **4a**

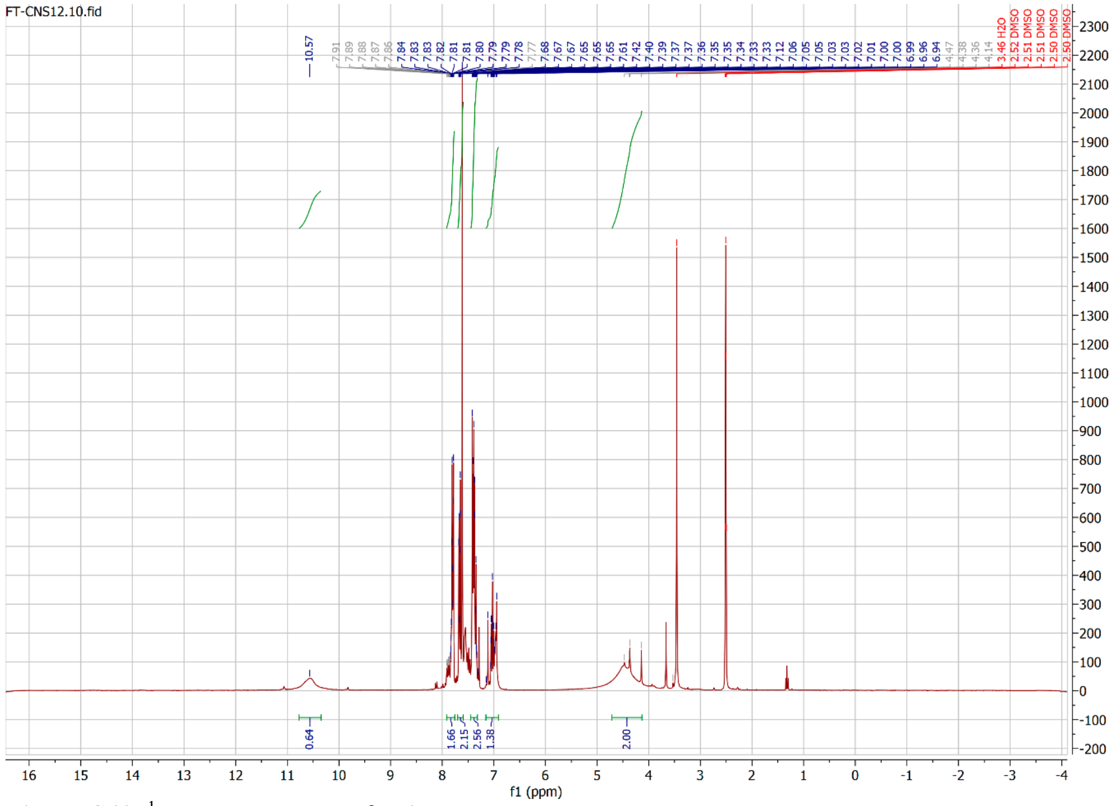

Figure S43. ^{1}H-NMR spectrum for **4a**

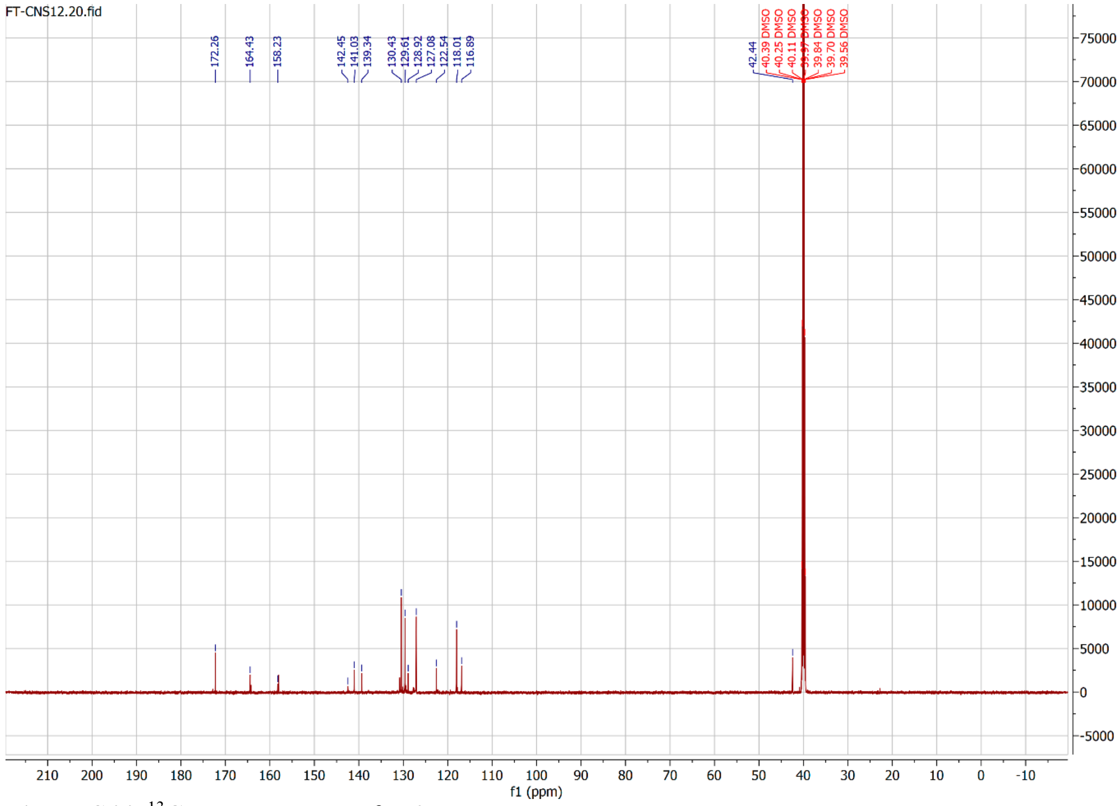

Figure S44. ^{13}C-NMR spectrum for **4a**

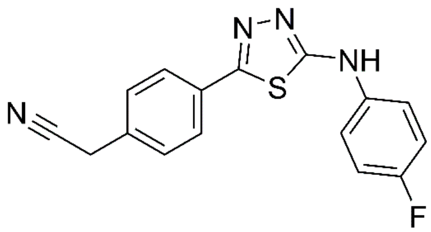

Figure S45. The chemical structure of compound **4b**

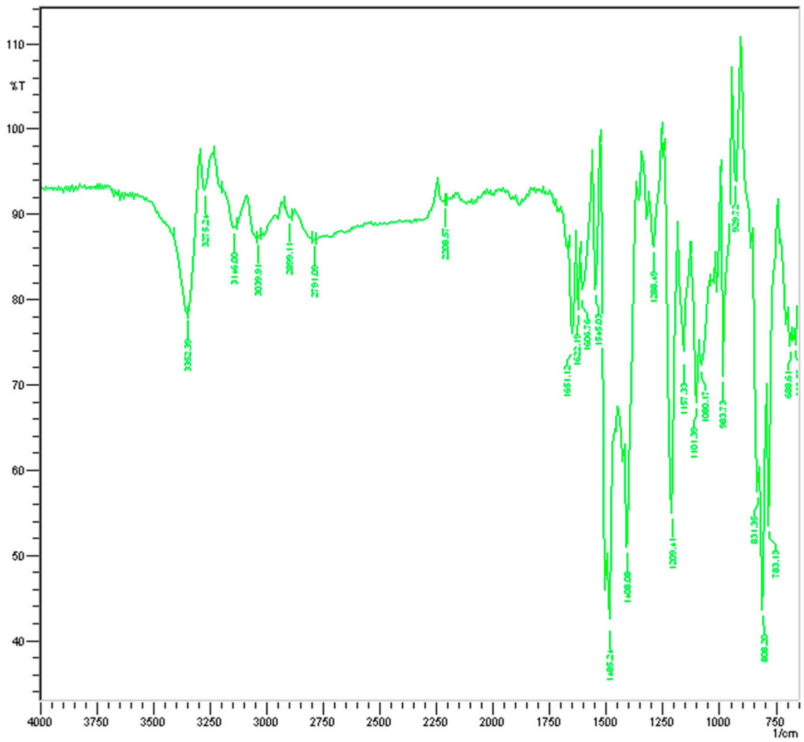

Figure S46. IR spectrum for **4b**

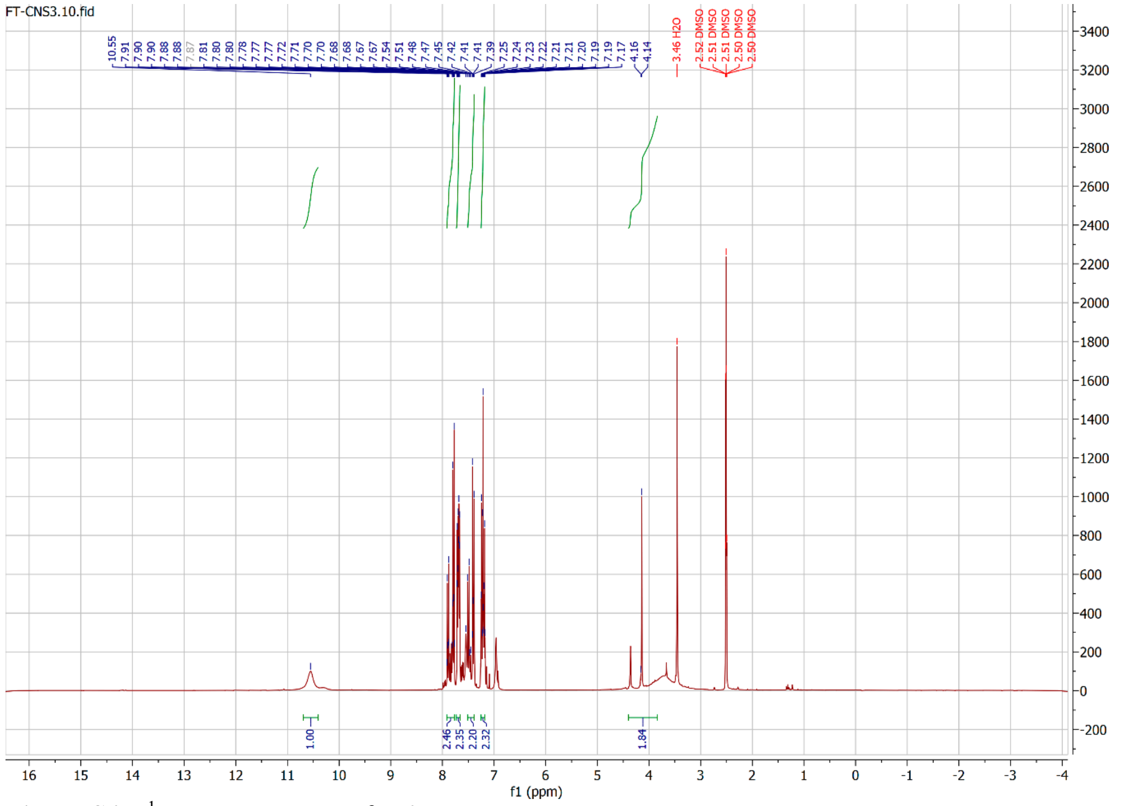

Figure S47. ^{1}H-NMR spectrum for **4b**

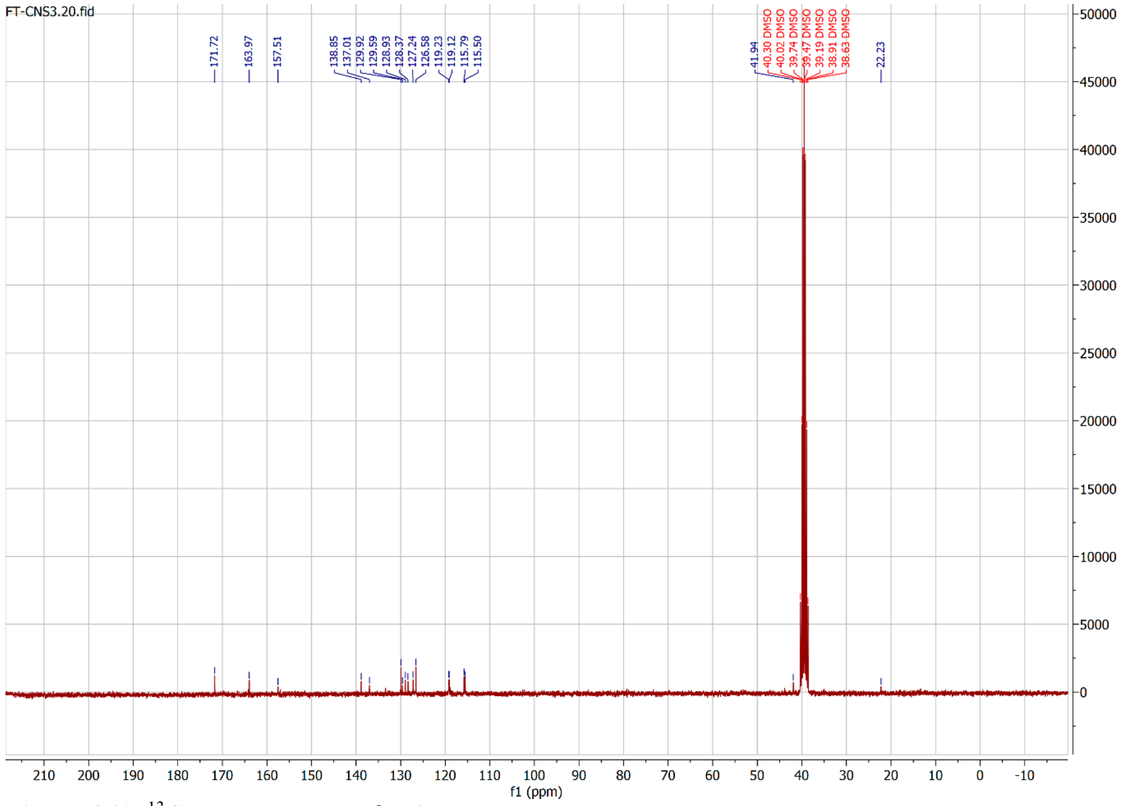

Figure S48. ^{13}C-NMR spectrum for **4b**

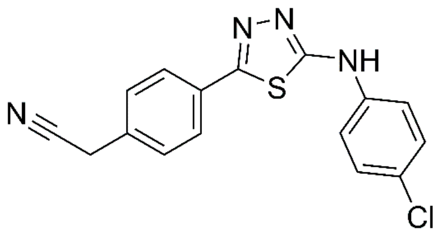

Figure S49. The chemical structure of compound **4c**

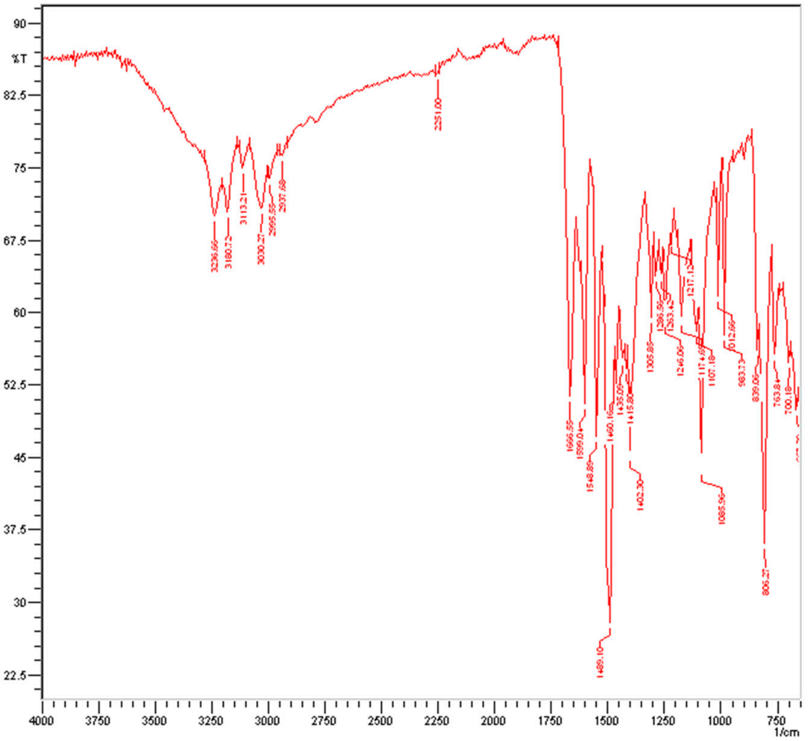

Figure S50. IR spectrum for **4c**

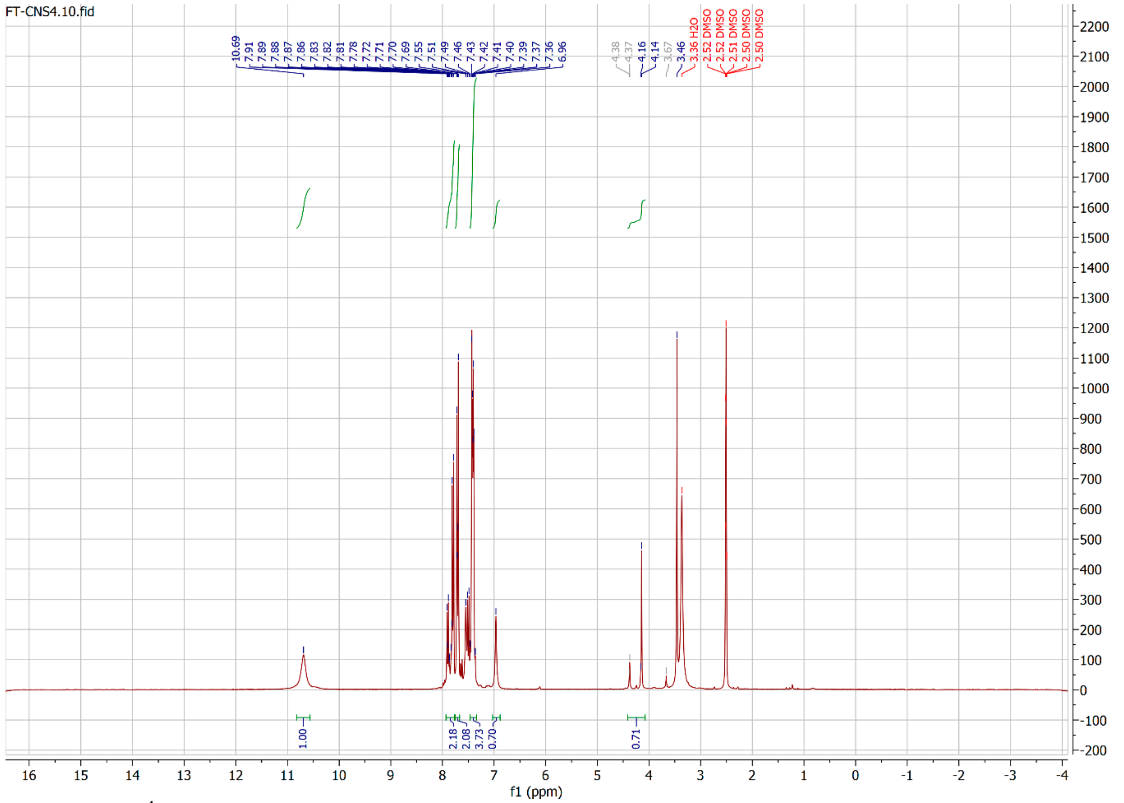

Figure S51. ^{1}H-NMR spectrum for **4c**

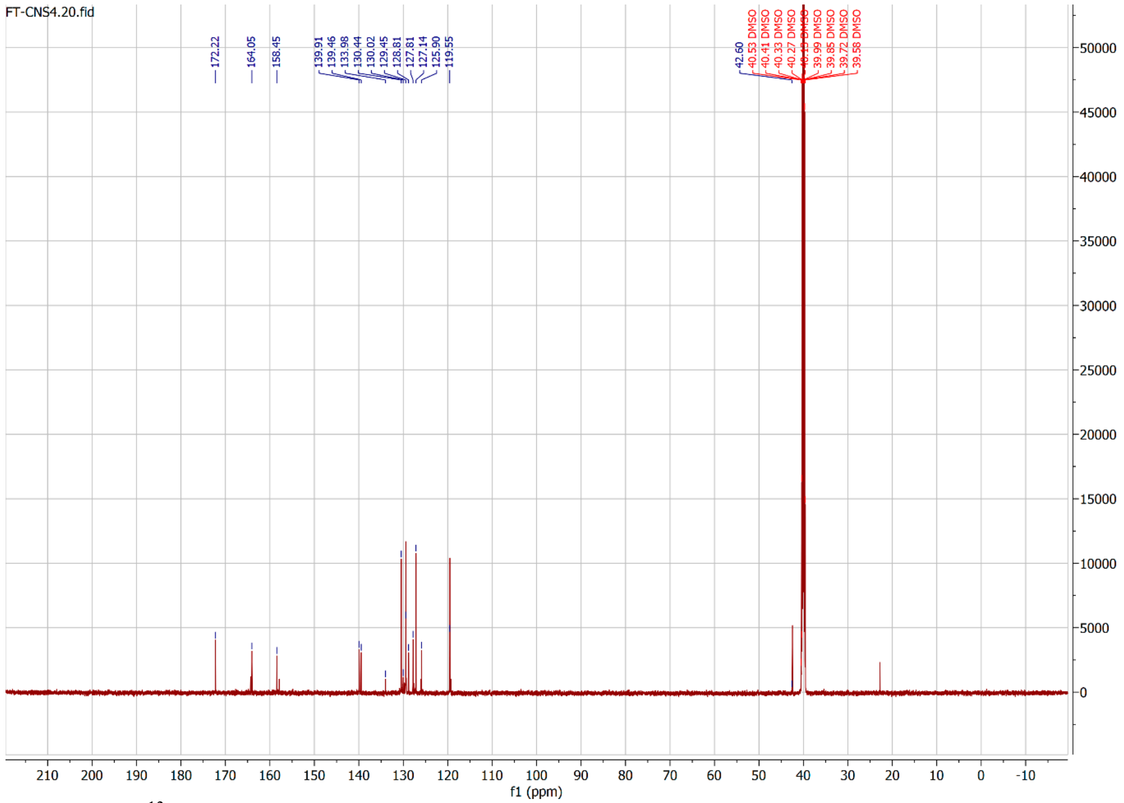

Figure S52. ^{13}C-NMR spectrum for **4c**

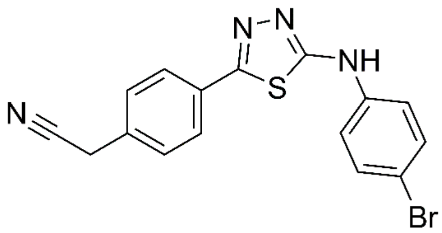

Figure S53. The chemical structure of compound **4d**

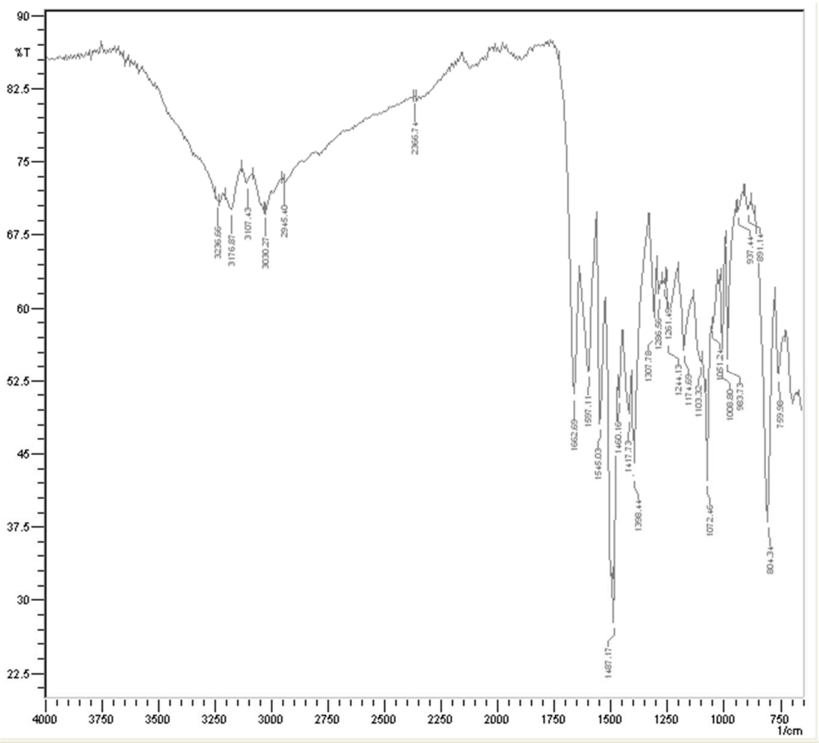

Figure S54. IR spectrum for **4d**

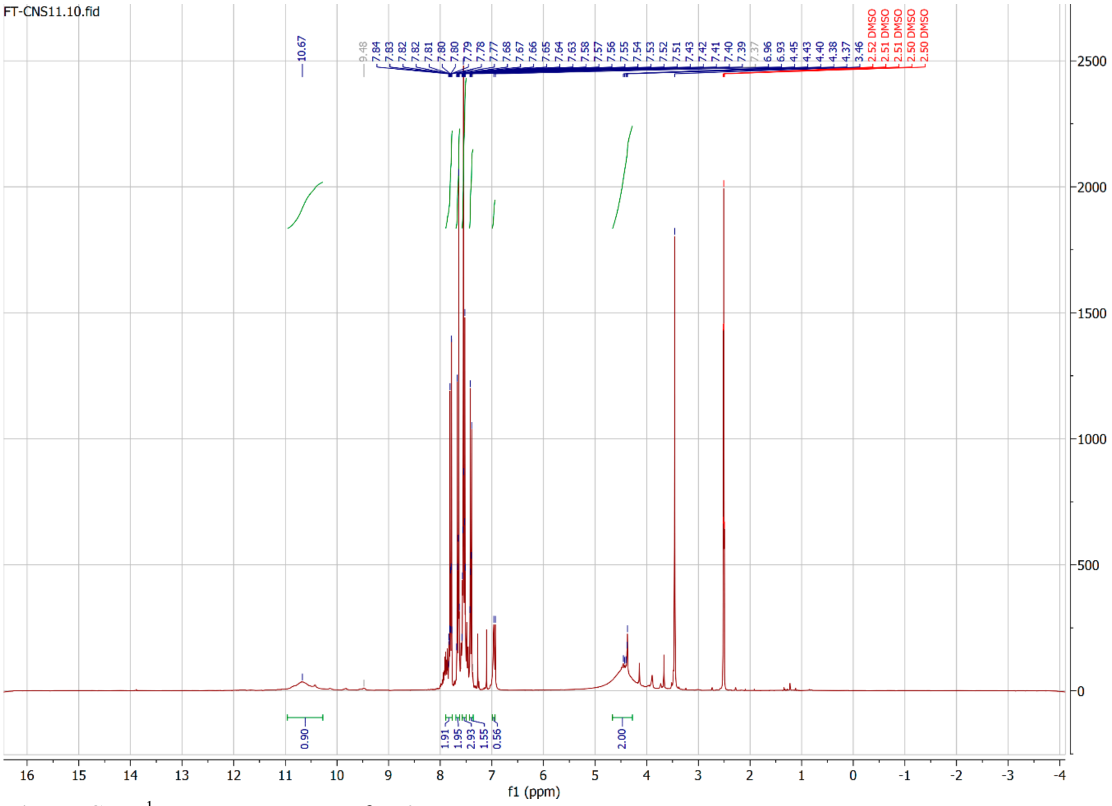

Figure S55. ^{1}H-NMR spectrum for **4d**

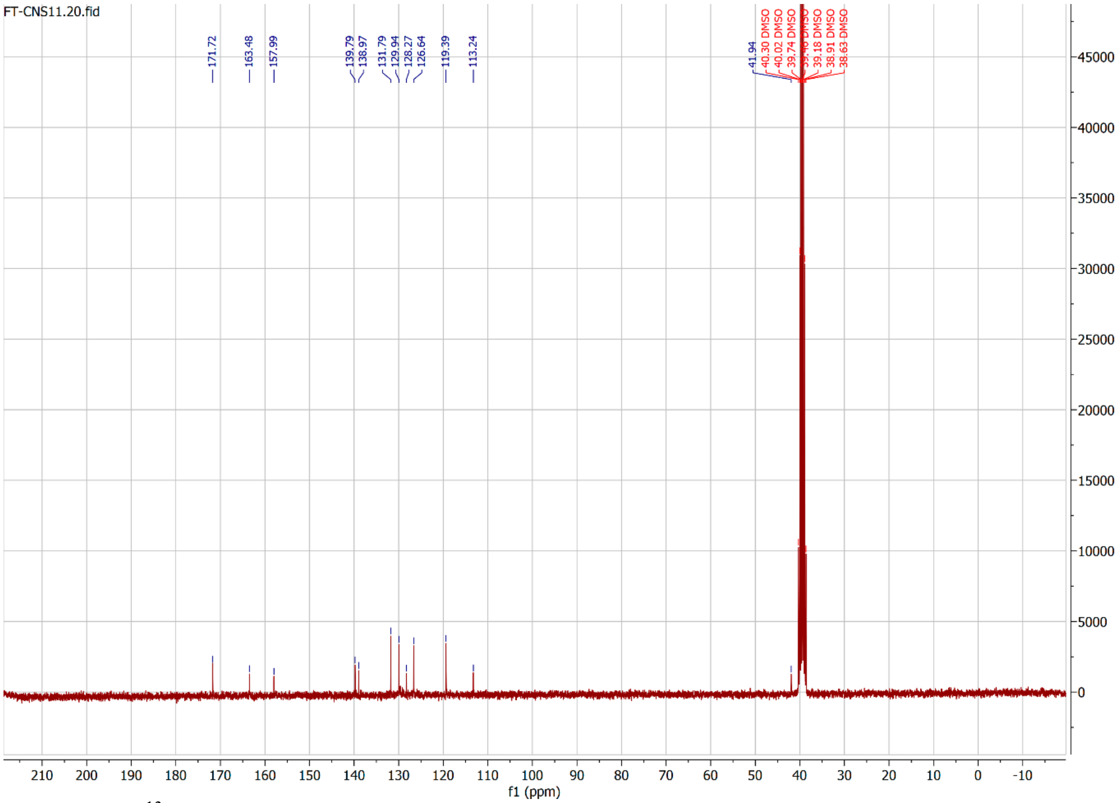

Figure S56. ^{13}C-NMR spectrum for **4d**

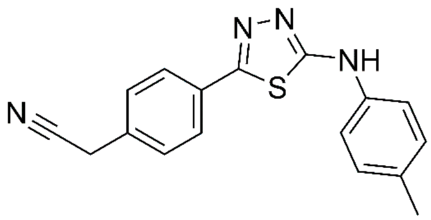

Figure S57. The chemical structure of compound **4e**

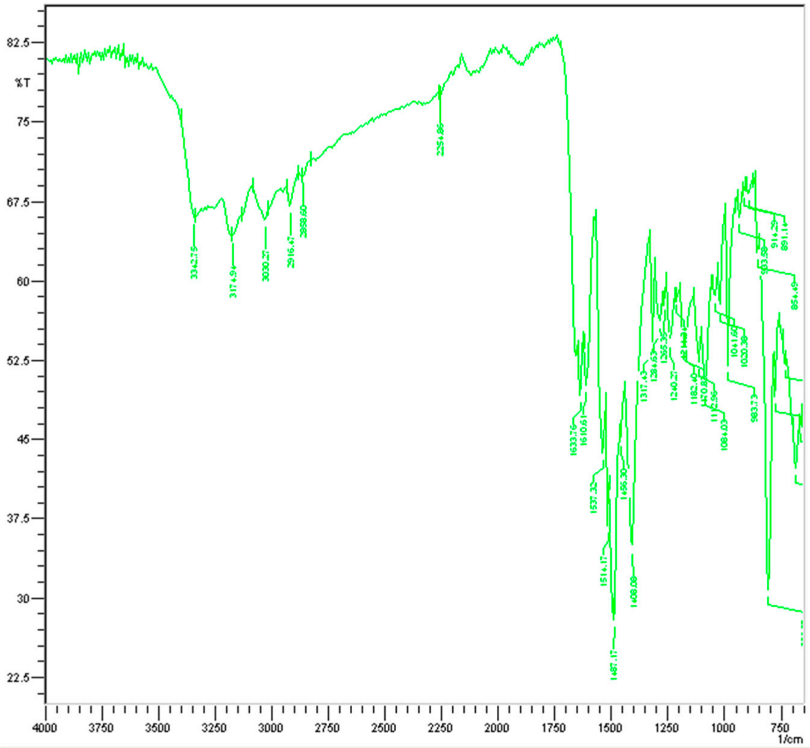

Figure S58. IR spectrum for **4e**

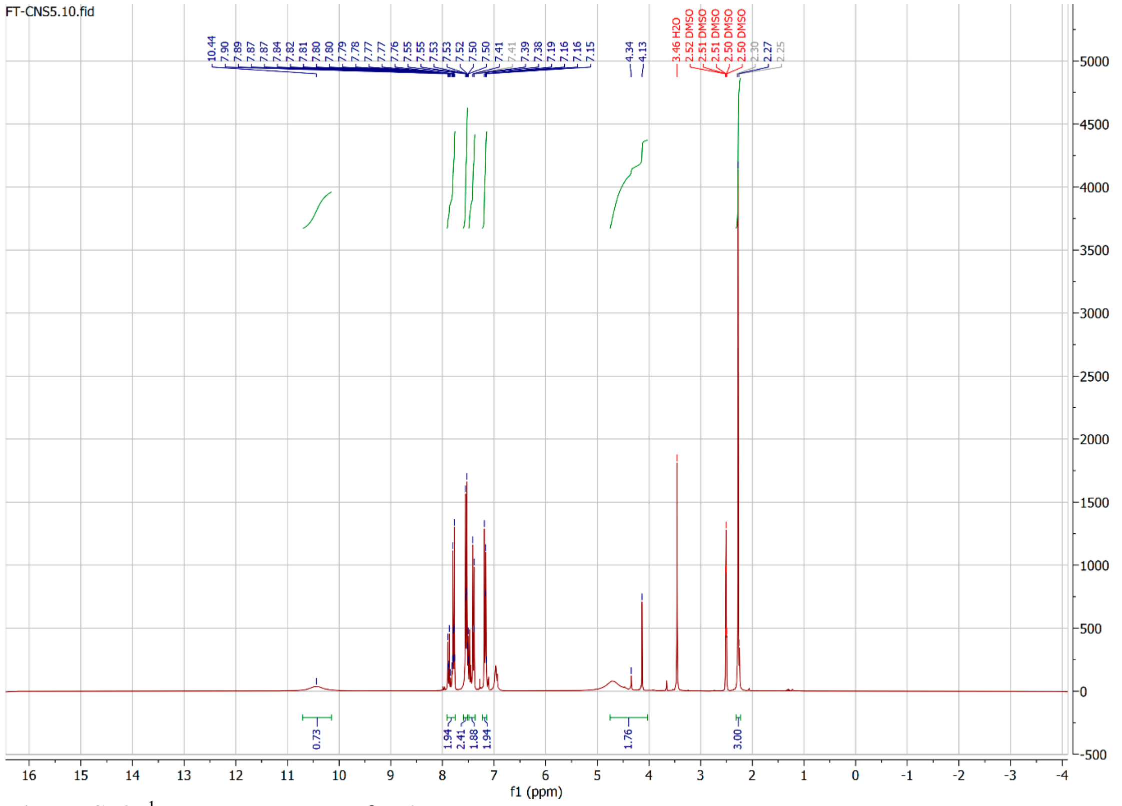

Figure S59. ^{1}H-NMR spectrum for **4e**

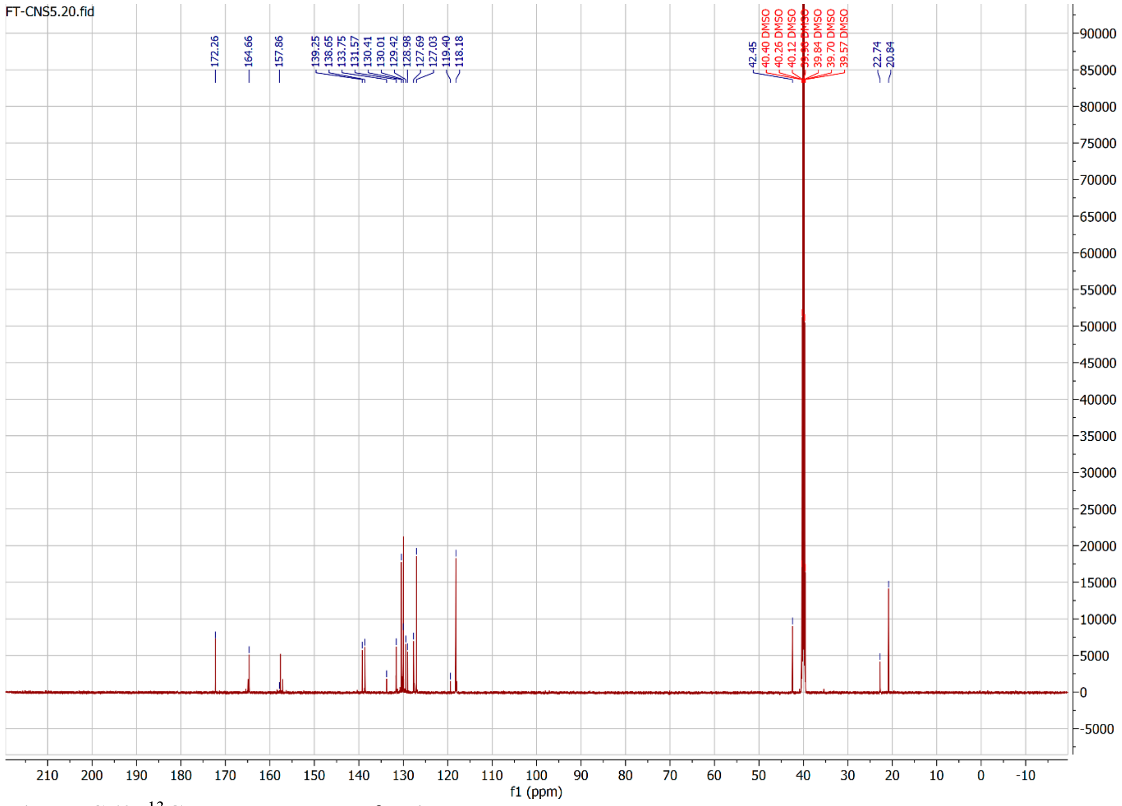

Figure S60. ^{13}C-NMR spectrum for **4e**

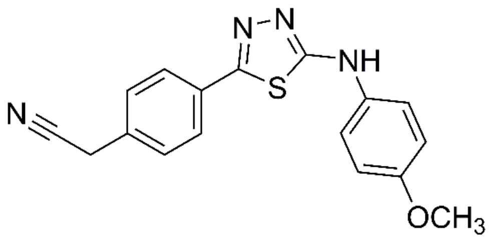

Figure S61. The chemical structure of compound **4f**

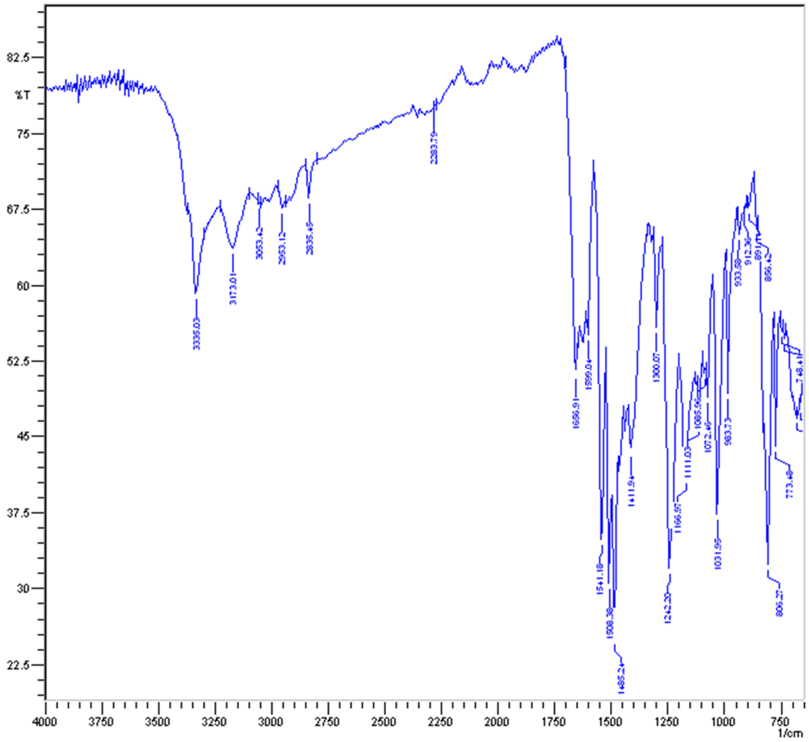

Figure S62. IR spectrum for **4f**

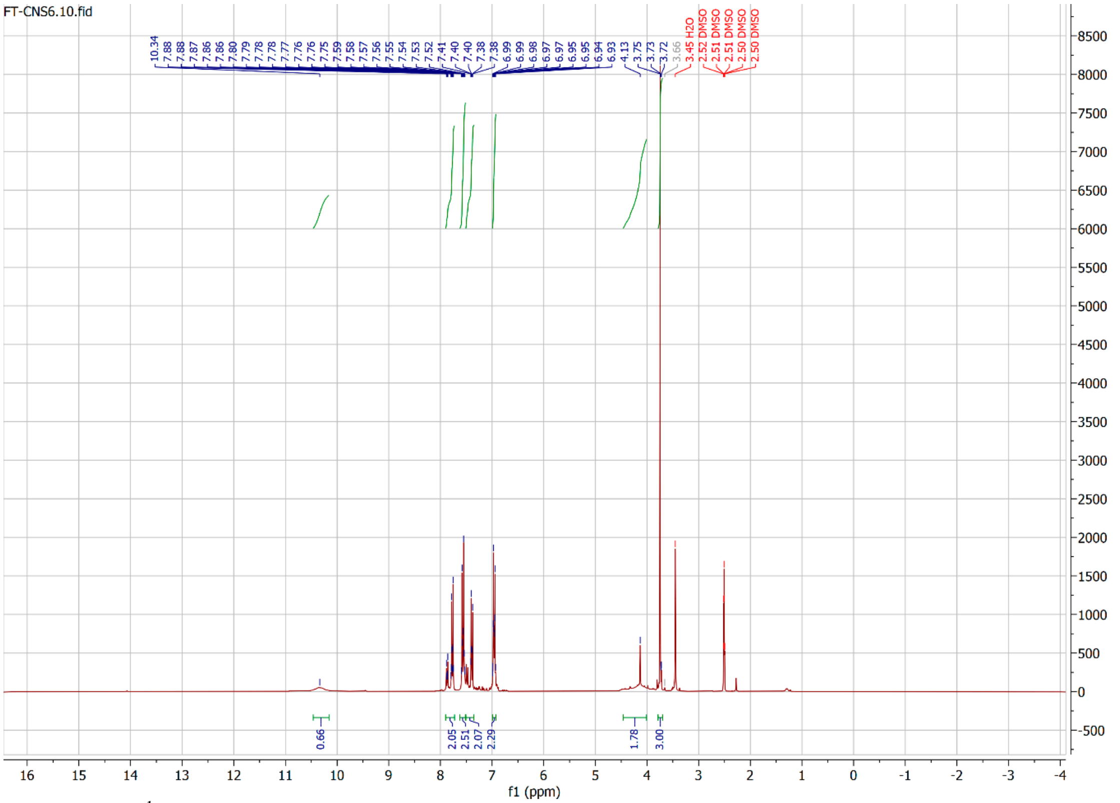

Figure S63. ^{1}H-NMR spectrum for **4f**

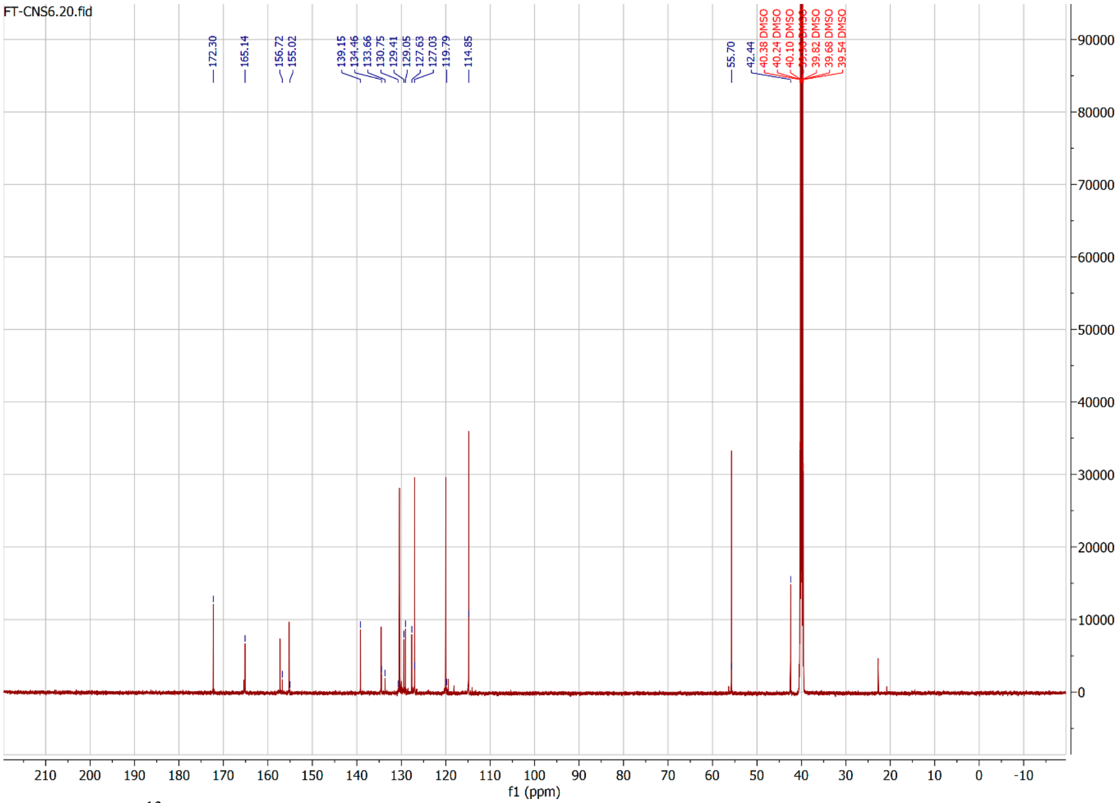

Figure S64. ^{13}C-NMR spectrum for **4f**

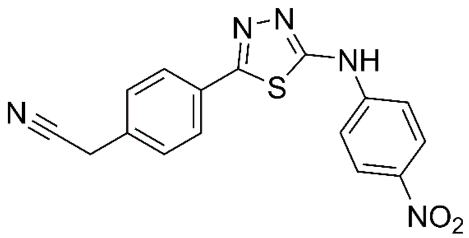

Figure S65. The chemical structure of compound **4g**

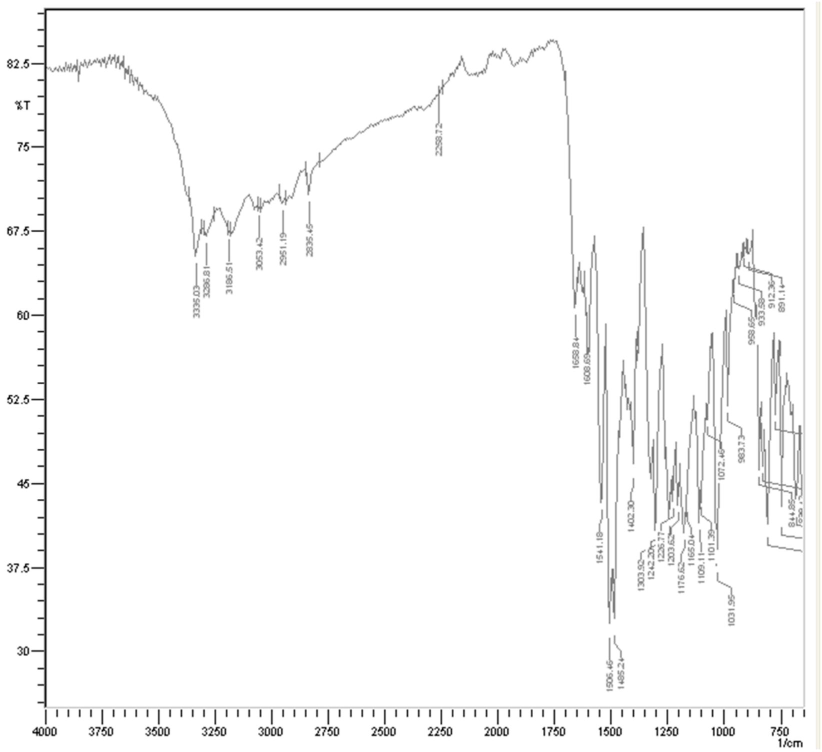

Figure S66. IR spectrum for **4g**

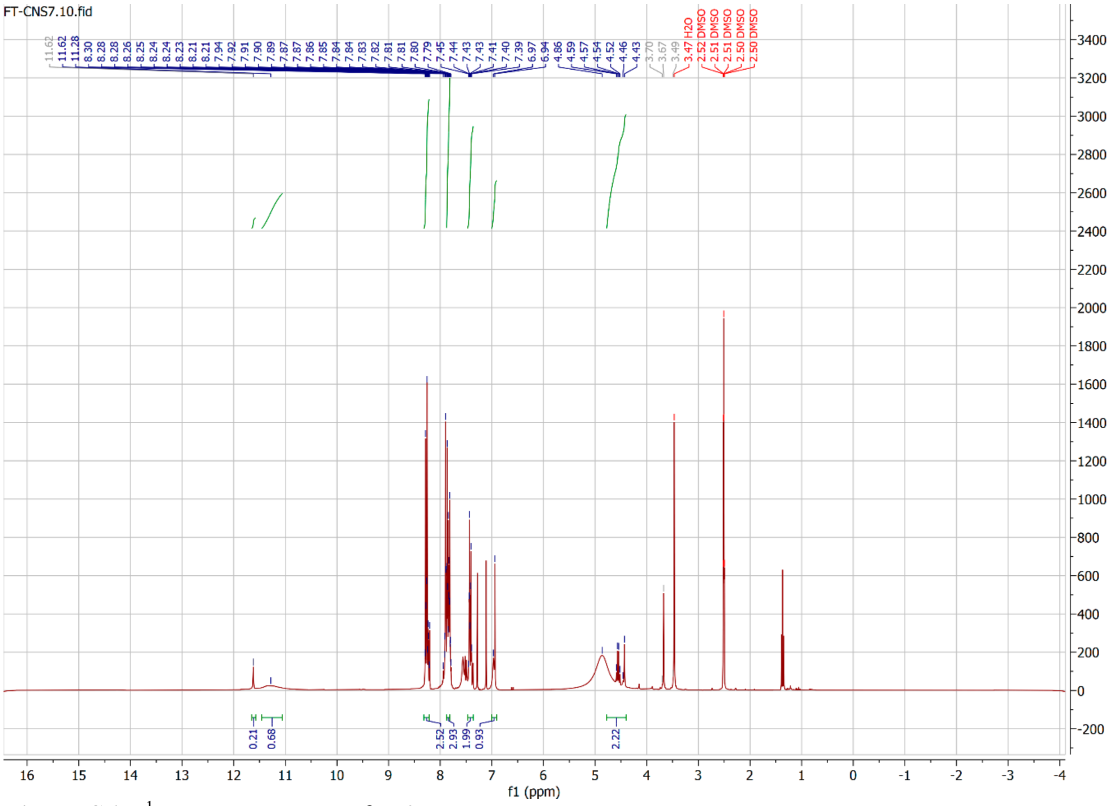

Figure S67. ^{1}H-NMR spectrum for **4g**

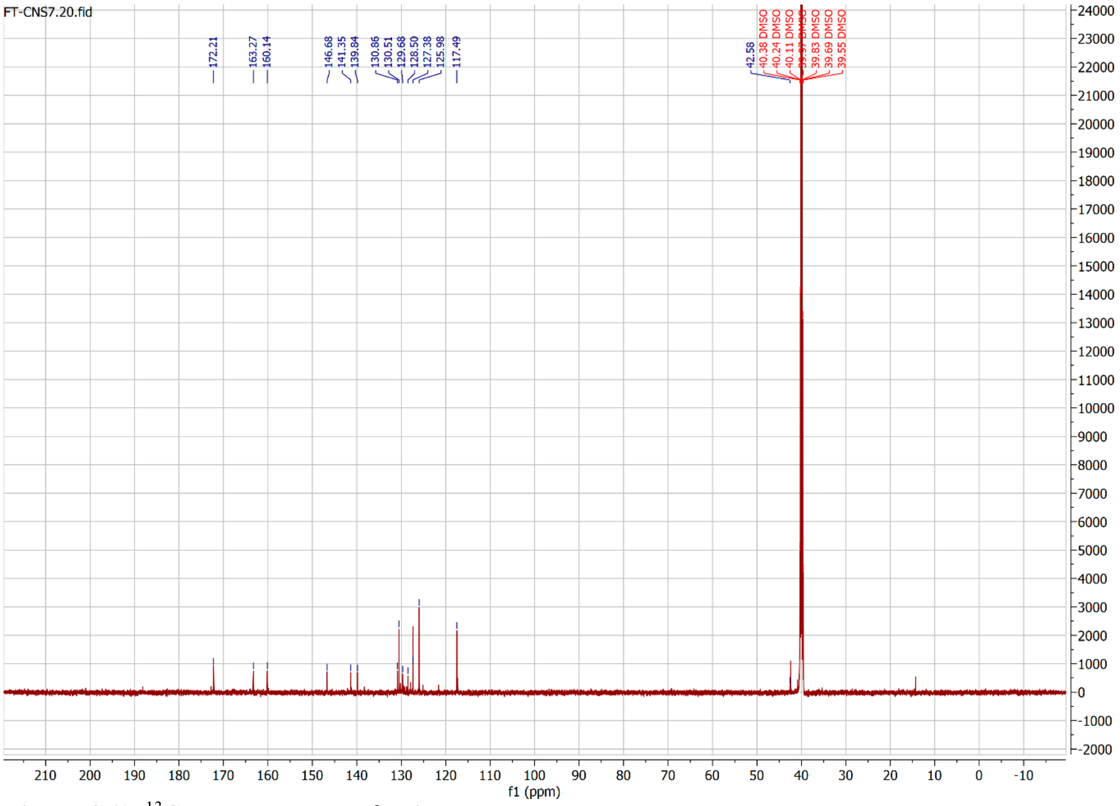

Figure S68. ^{13}C-NMR spectrum for **4g**

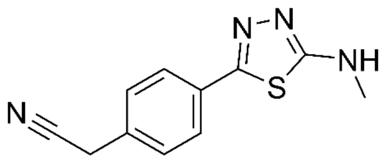

Figure S69. The chemical structure of compound **4h**

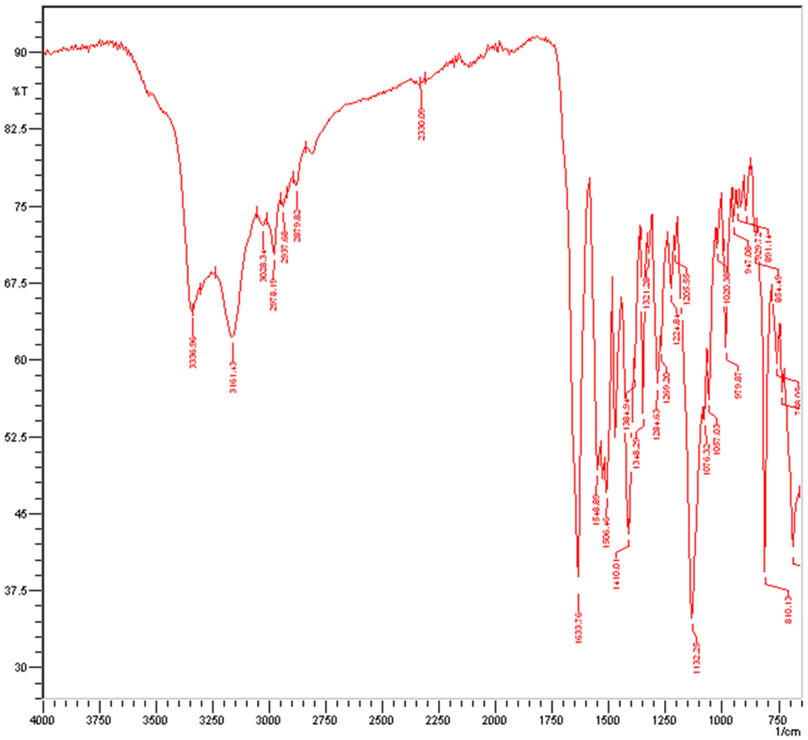

Figure S70. IR spectrum for **4h**

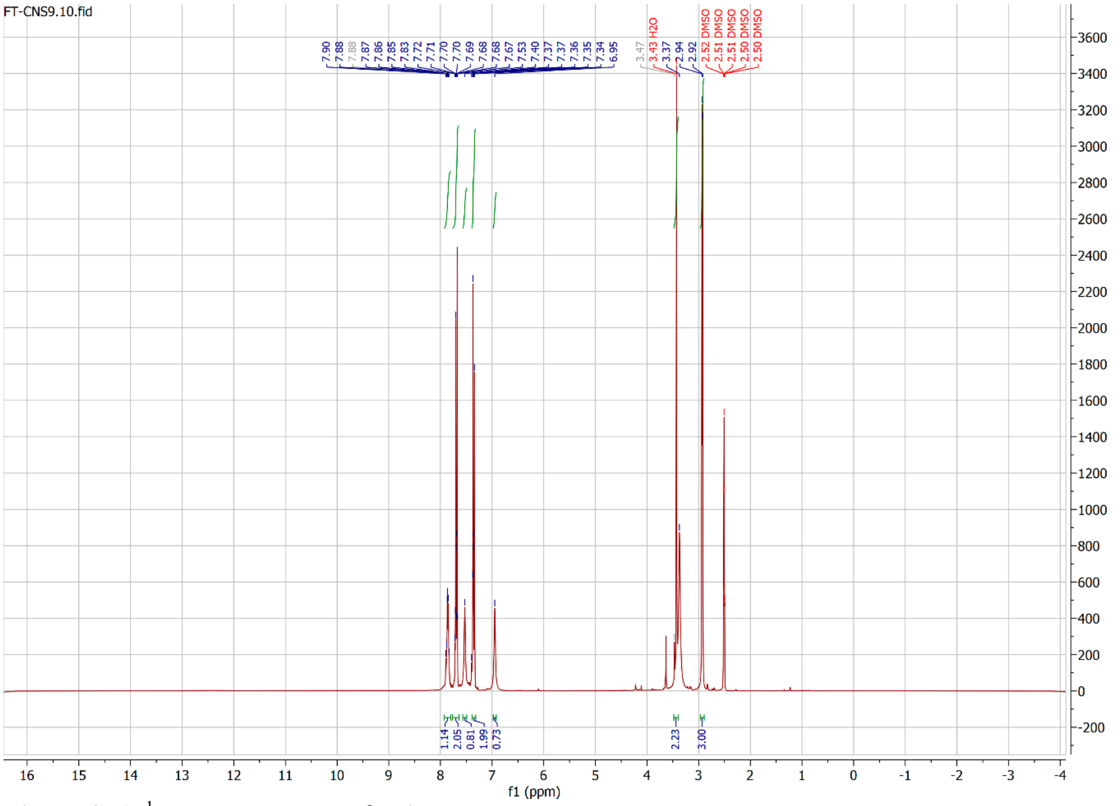

Figure S71. ^{1}H-NMR spectrum for **4h**

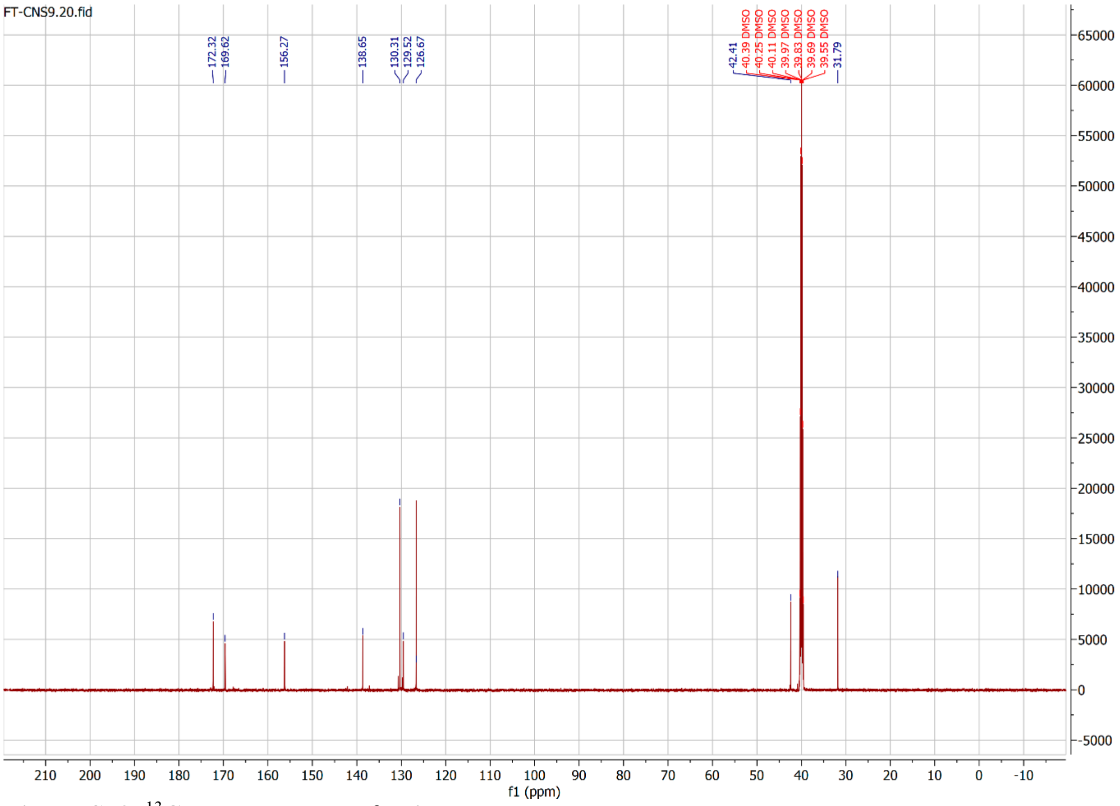

Figure S72. ^{13}C-NMR spectrum for **4h**

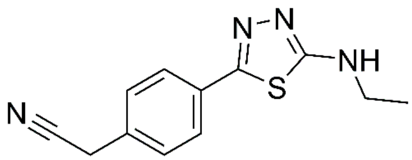

Figure S73. The chemical structure of compound **4i**

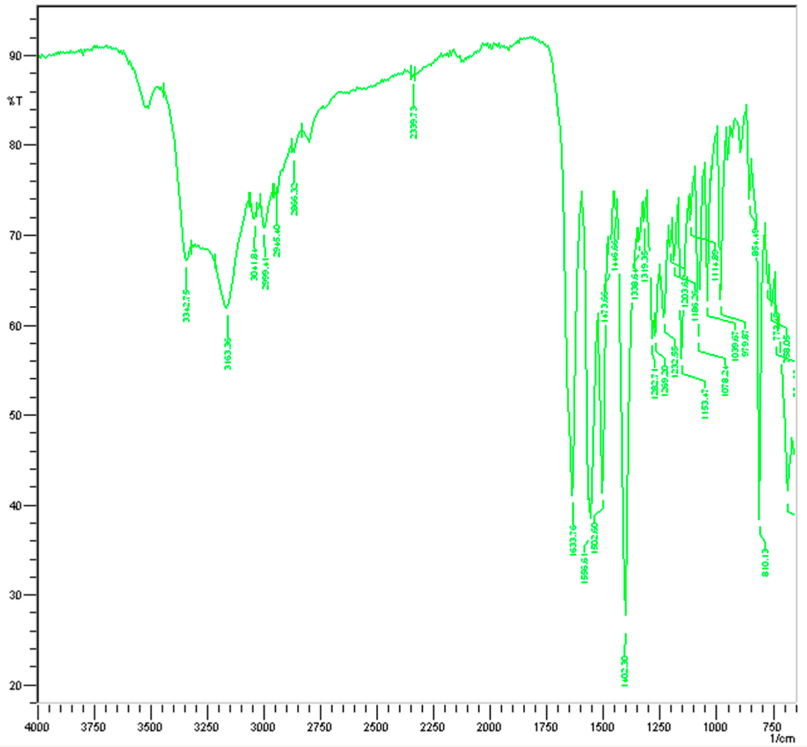

Figure S74. IR spectrum for **4i**

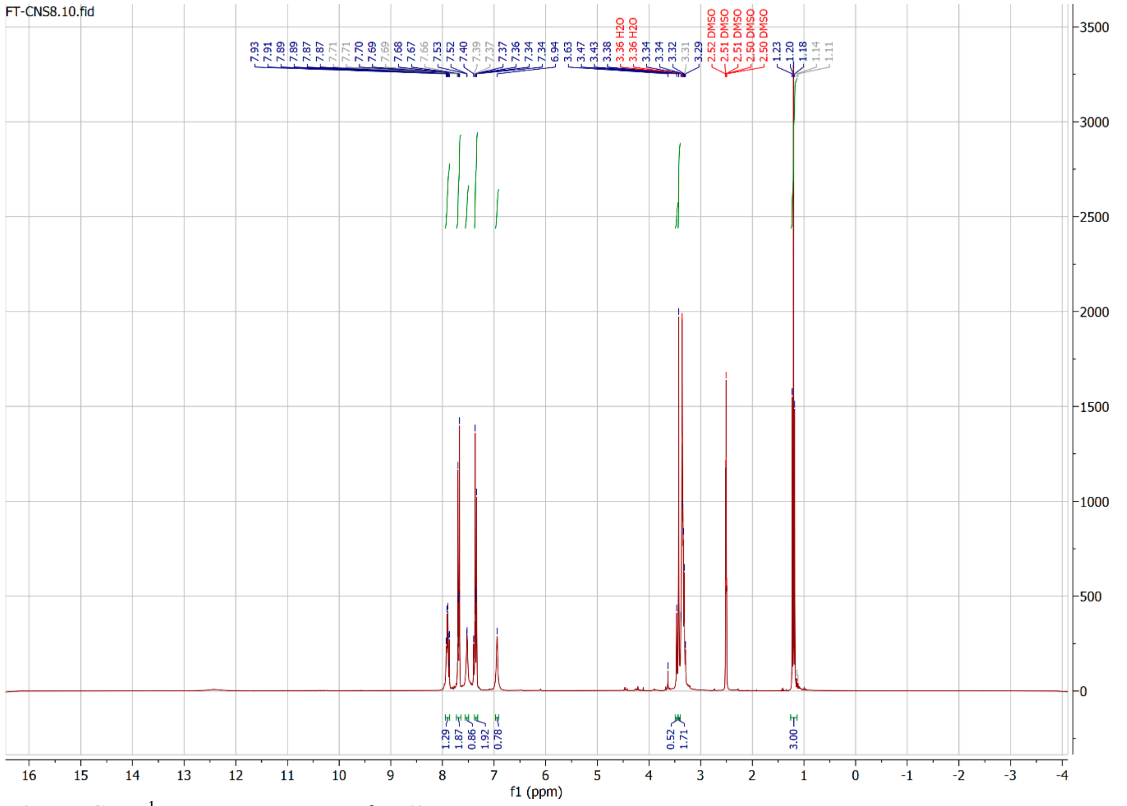

Figure S75. ^{1}H-NMR spectrum for **4i**

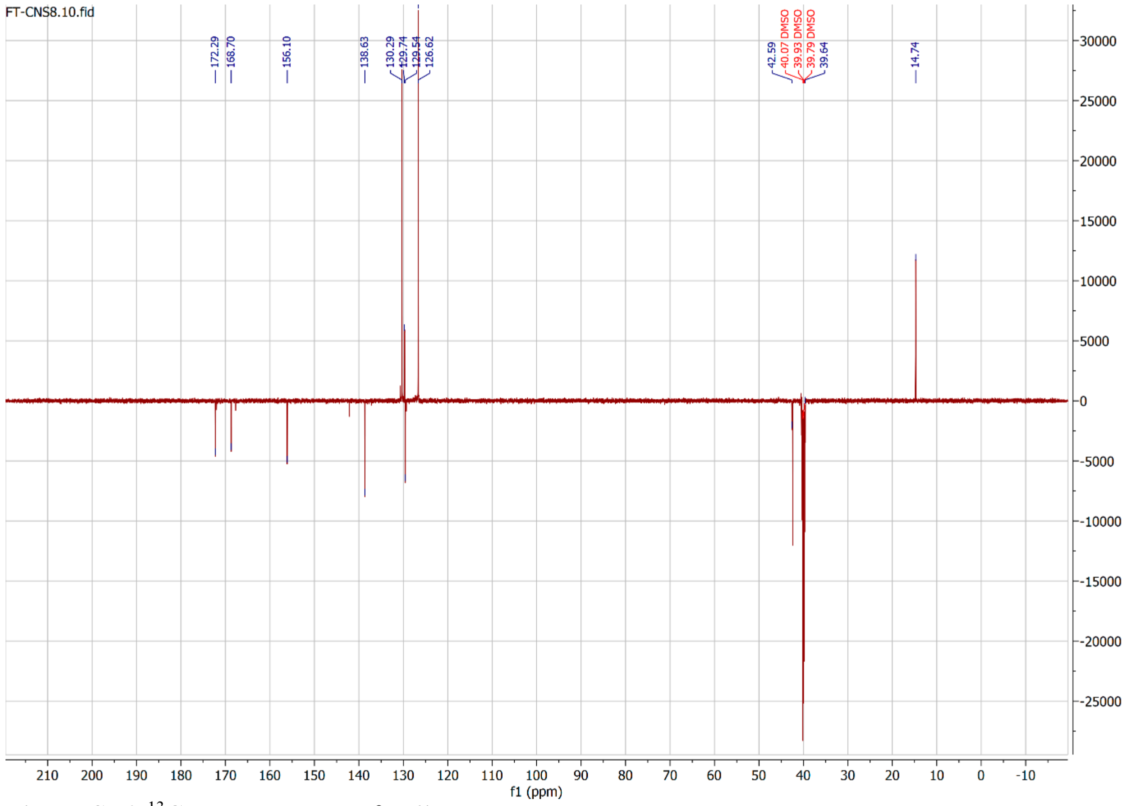

Figure S76. ^{13}C-NMR spectrum for **4i**

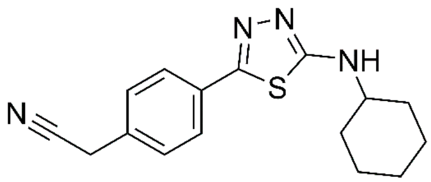

Figure S77. The chemical structure of compound **4j**

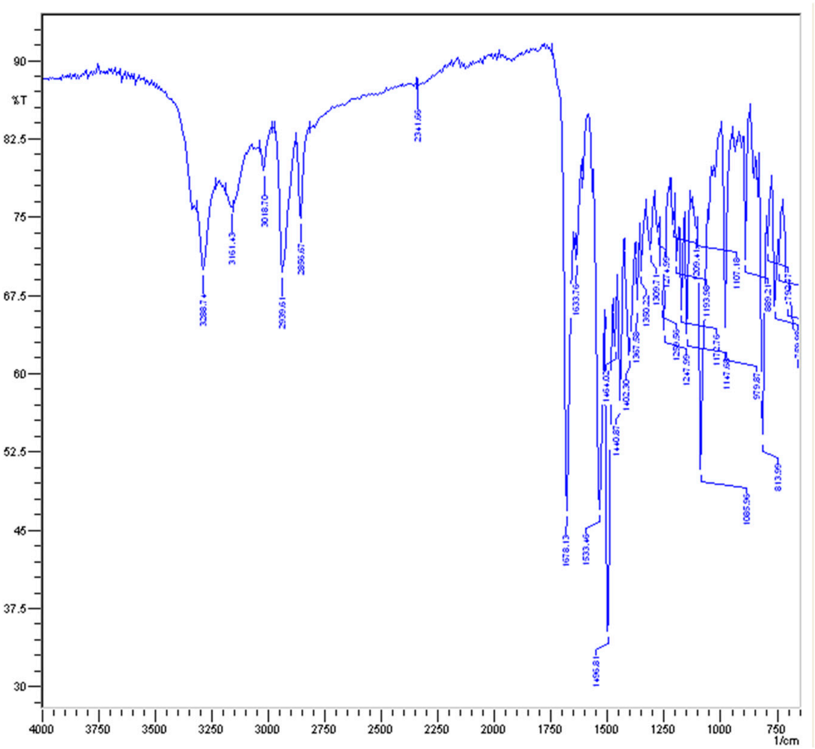

Figure S78. IR spectrum for **4j**

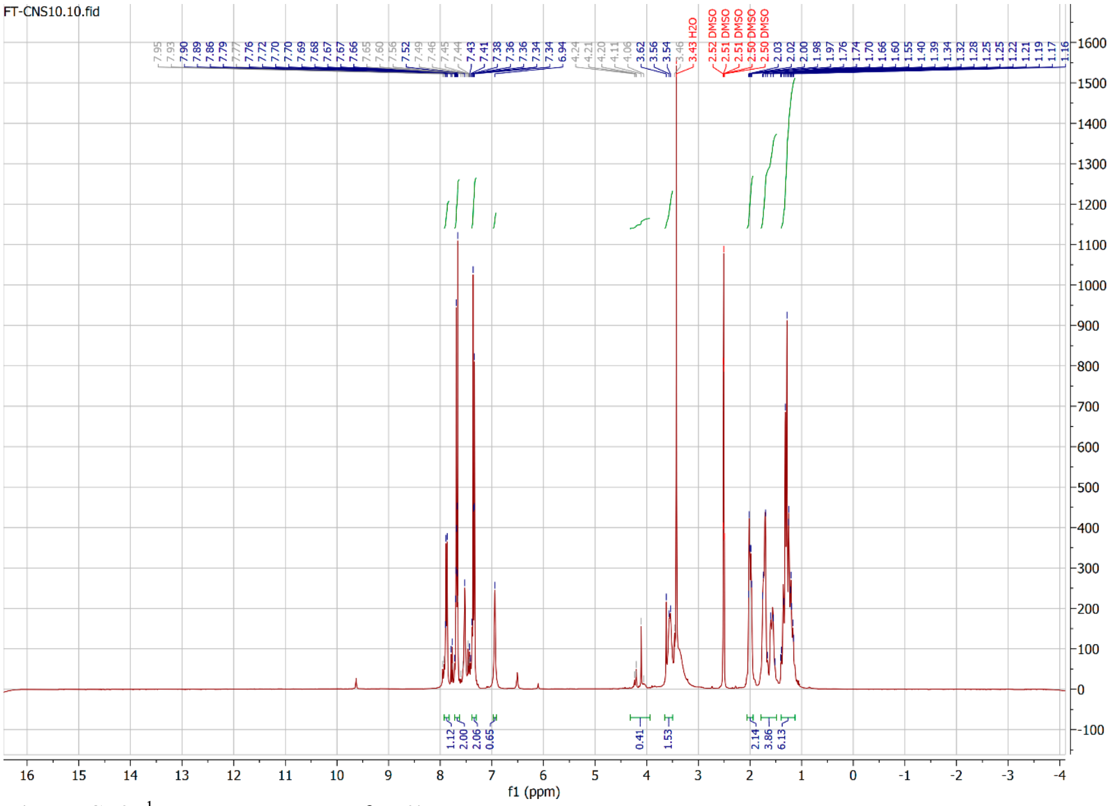

Figure S79. ^{1}H-NMR spectrum for **4j**

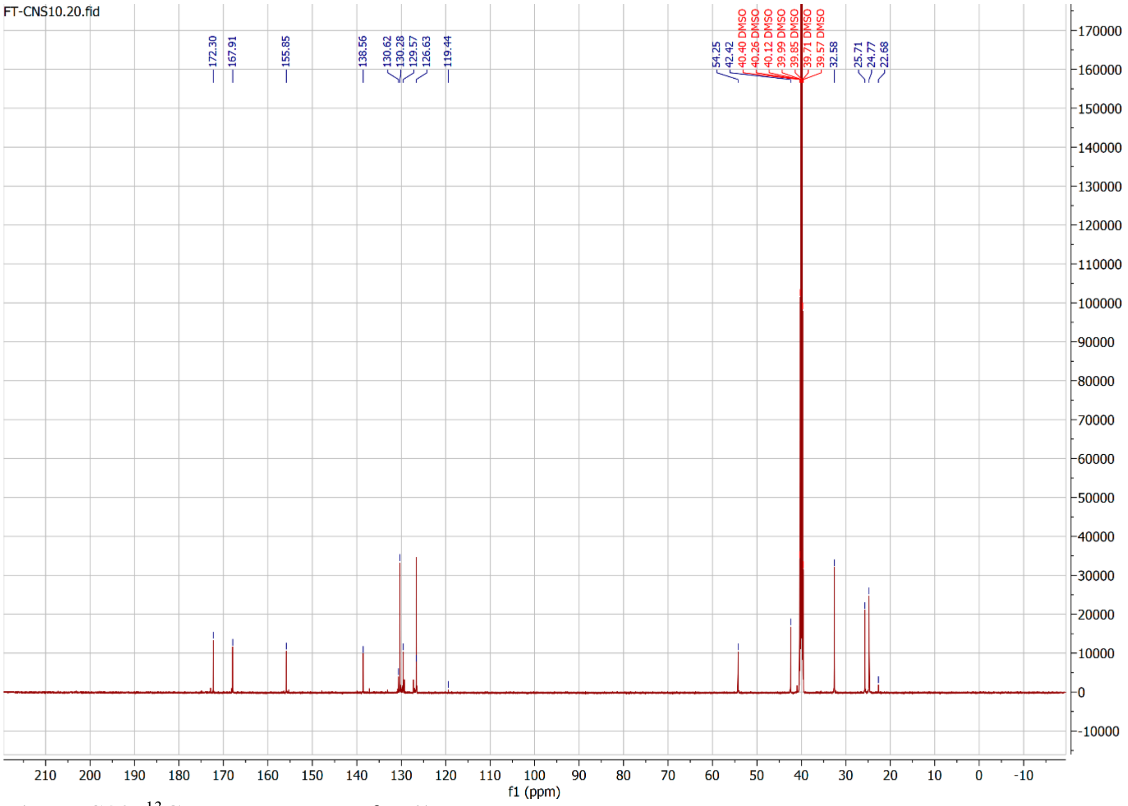

Figure S80. ^{13}C-NMR spectrum for **4j**

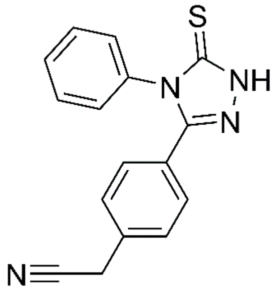

Figure S81. The chemical structure of compound **5a**

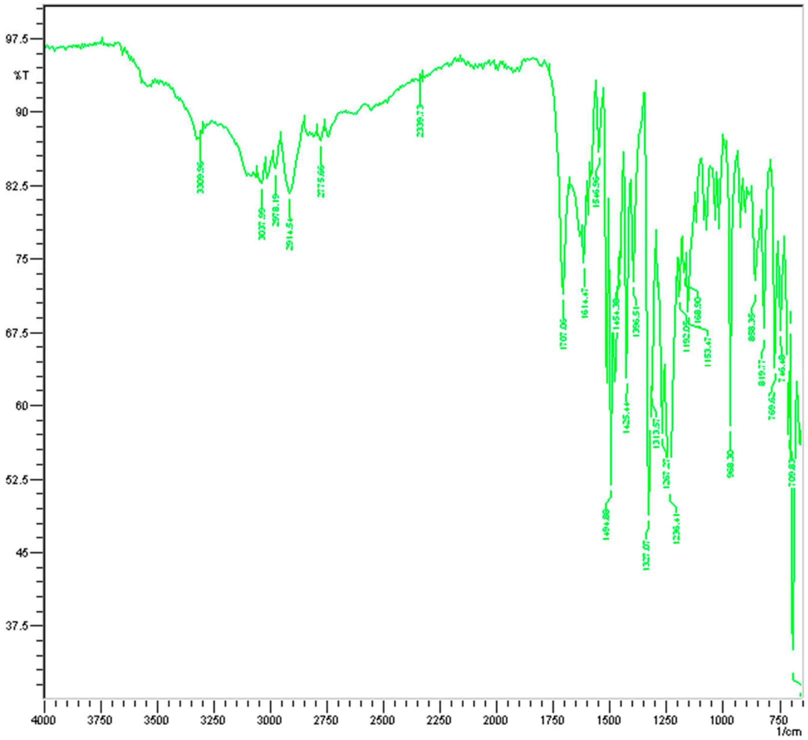

Figure S82. IR spectrum for **5a**

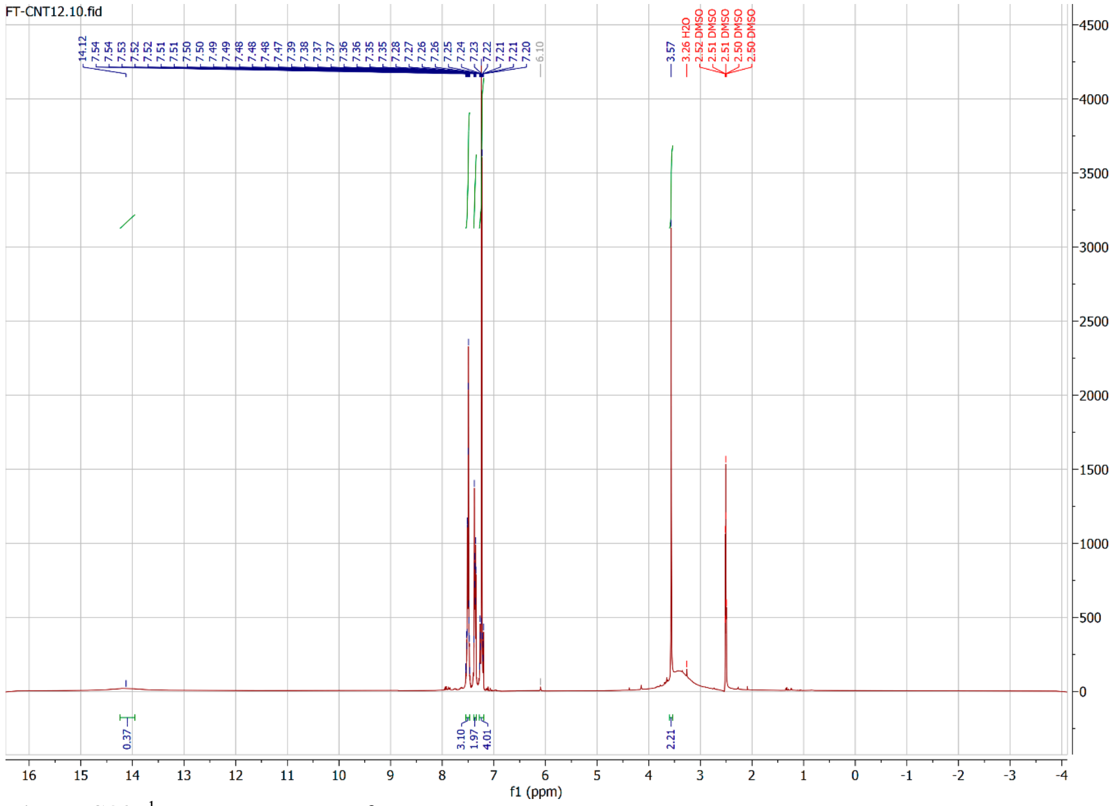

Figure S83. ^{1}H-NMR spectrum for **5a**

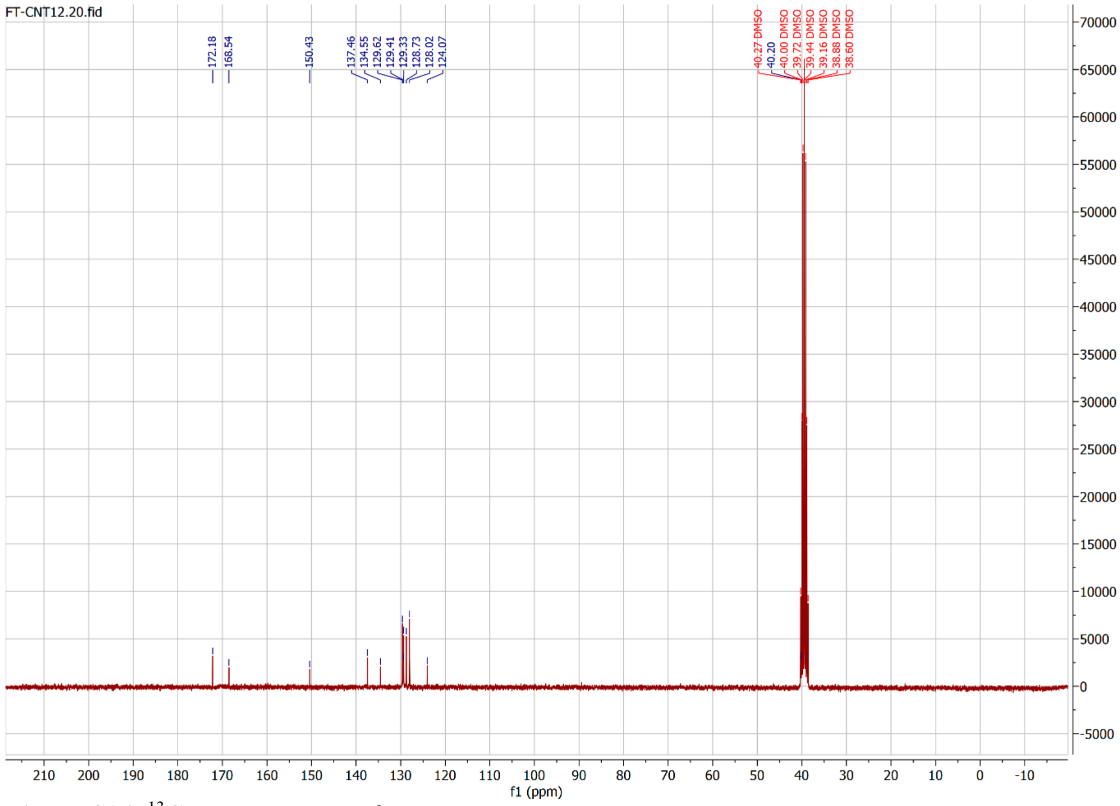

Figure S84. ^{13}C-NMR spectrum for **5a**

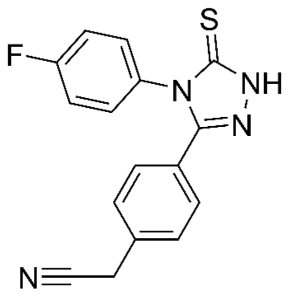

Figure S85. The chemical structure of compound **5b**

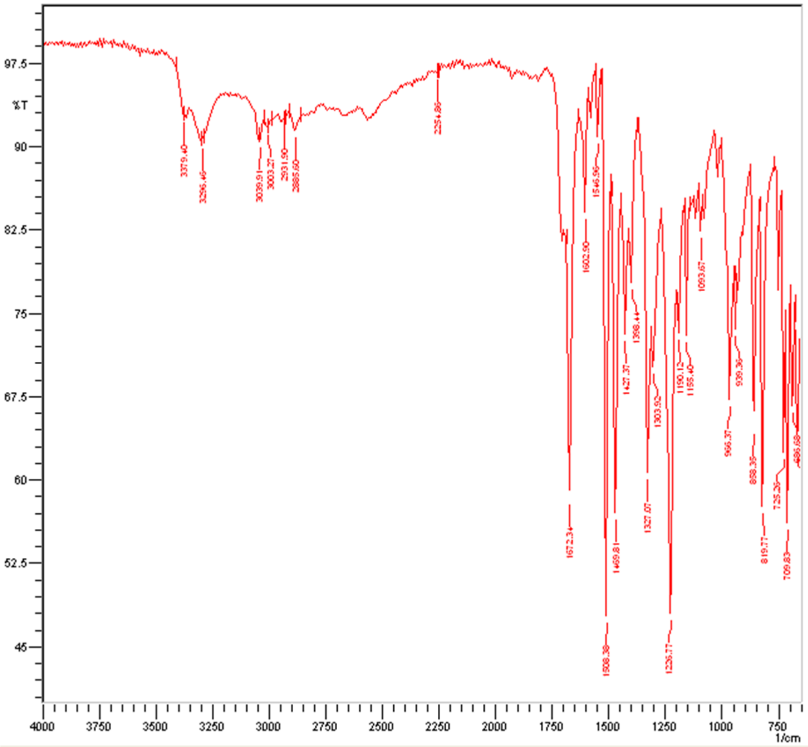

Figure S86. IR spectrum for **5b**

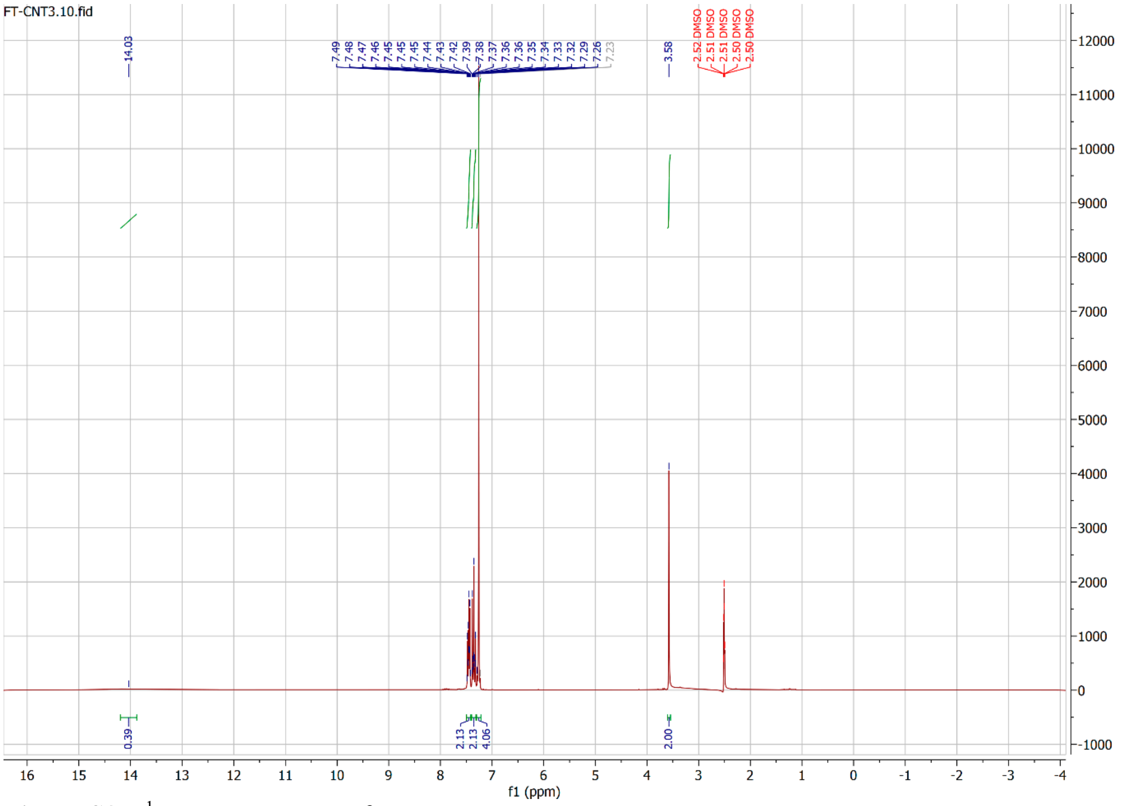

Figure S87. ^{1}H-NMR spectrum for **5b**

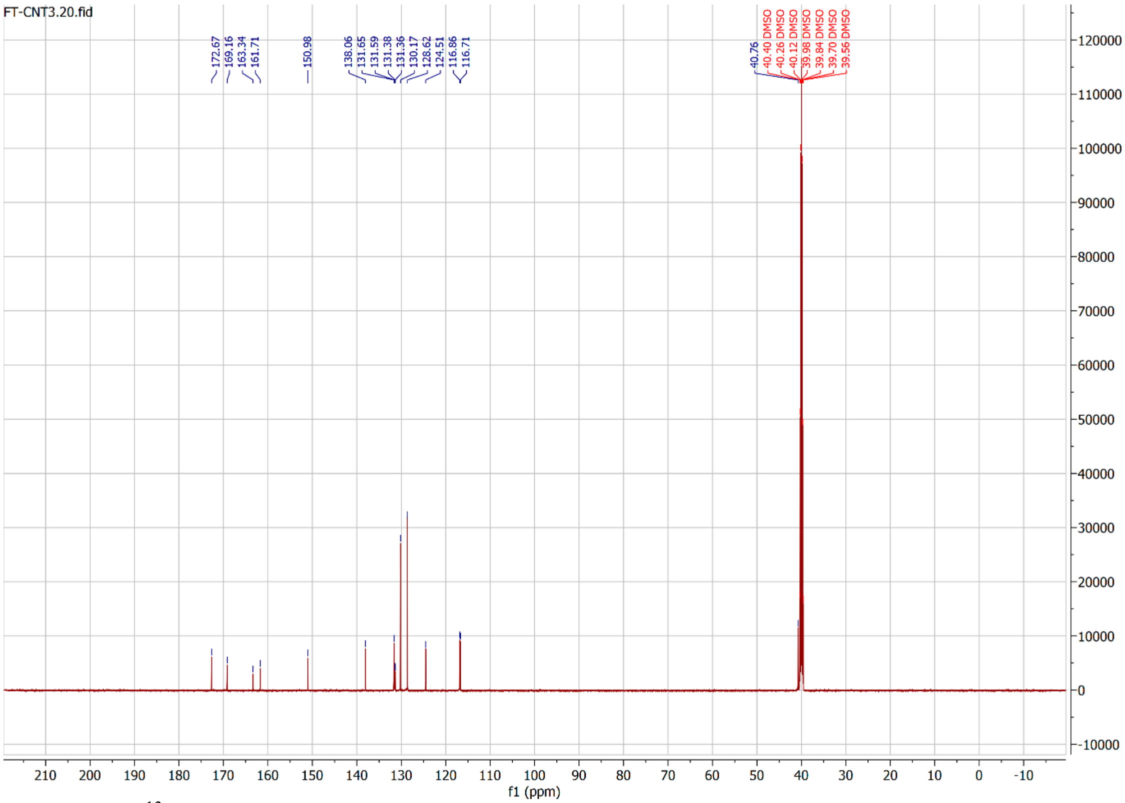

Figure S88. ^{13}C-NMR spectrum for **5b**

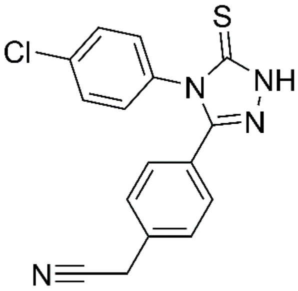

Figure S89. The chemical structure of compound **5c**

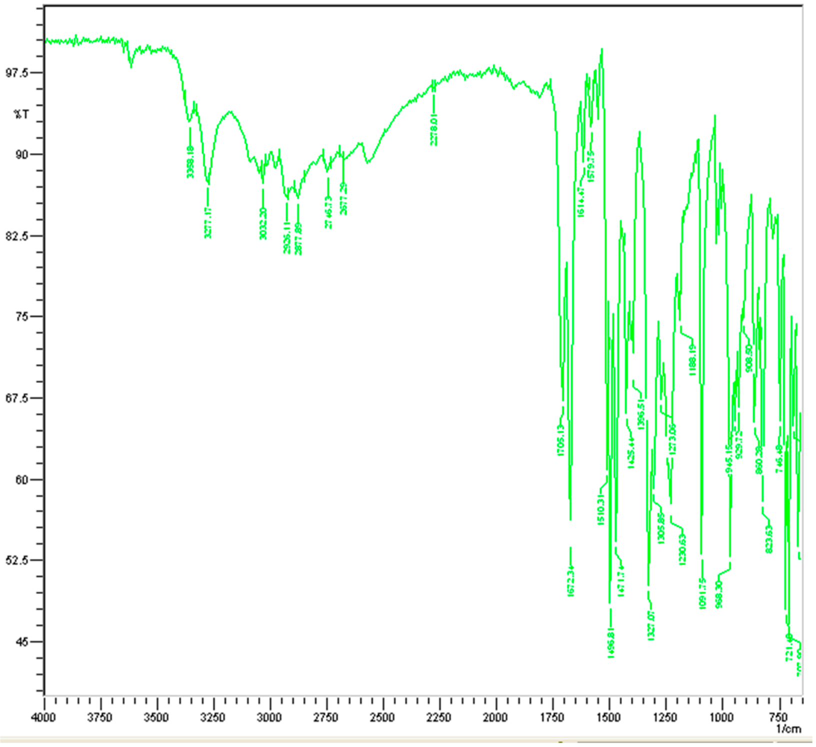

Figure S90. IR spectrum for **5c**

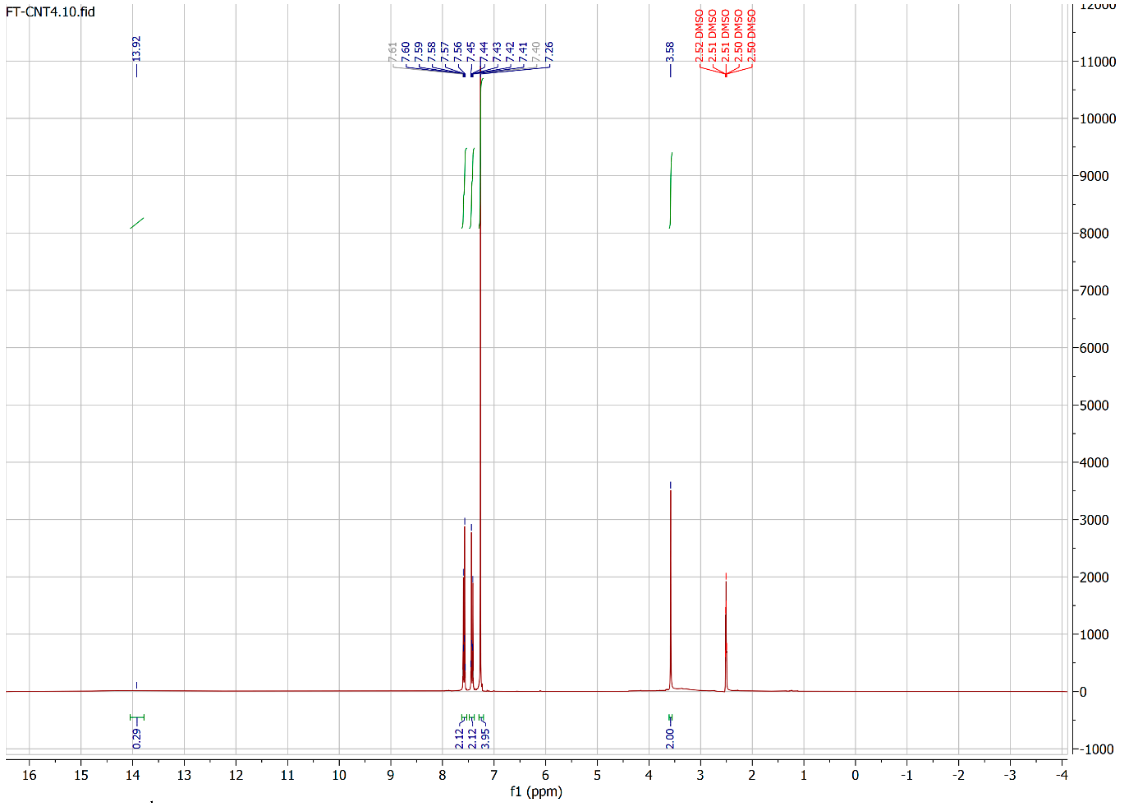

Figure S91. ^{1}H-NMR spectrum for **5c**

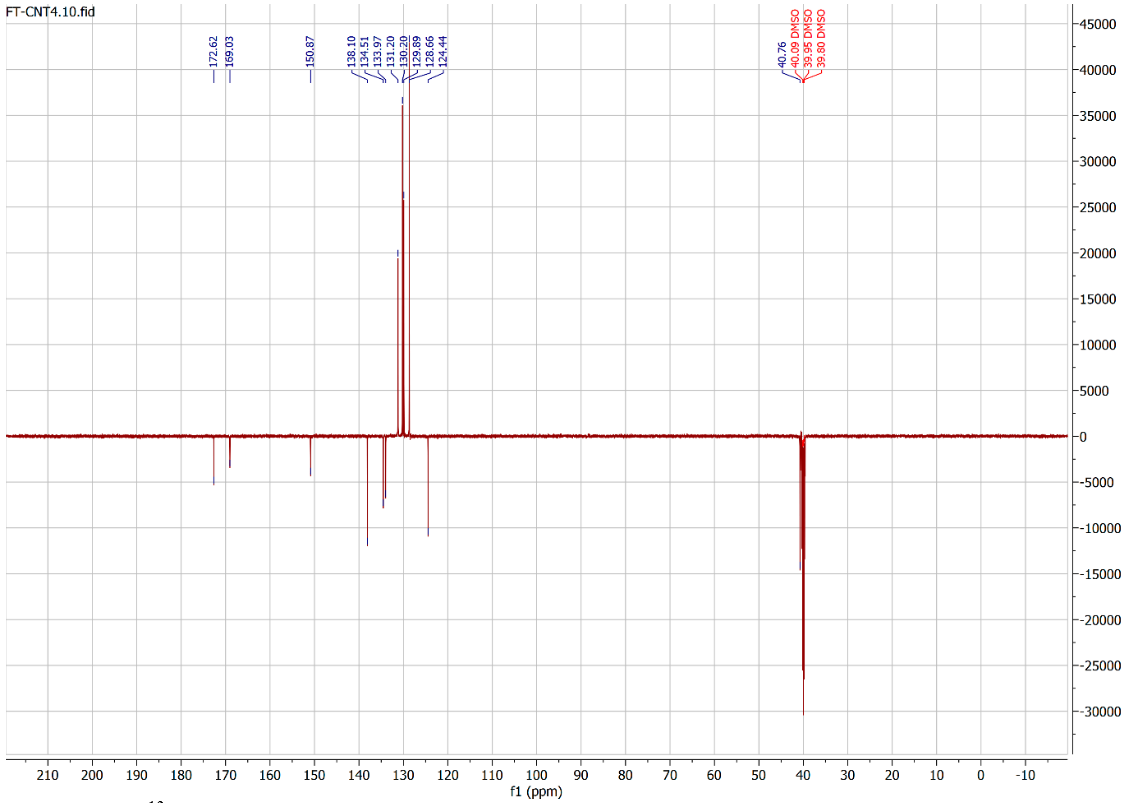

Figure S92. ^{13}C-NMR spectrum for **5c**

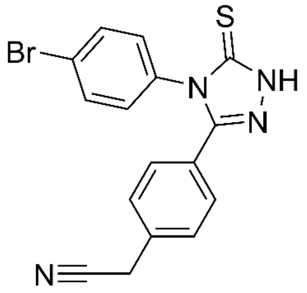

Figure S93. The chemical structure of compound **5d**

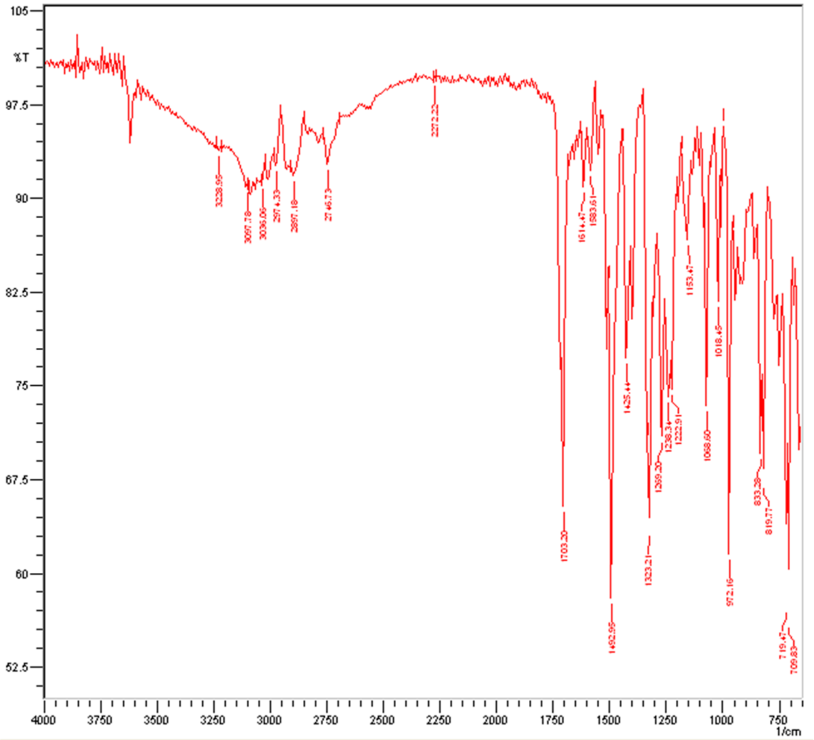

Figure S94. IR spectrum for **5d**

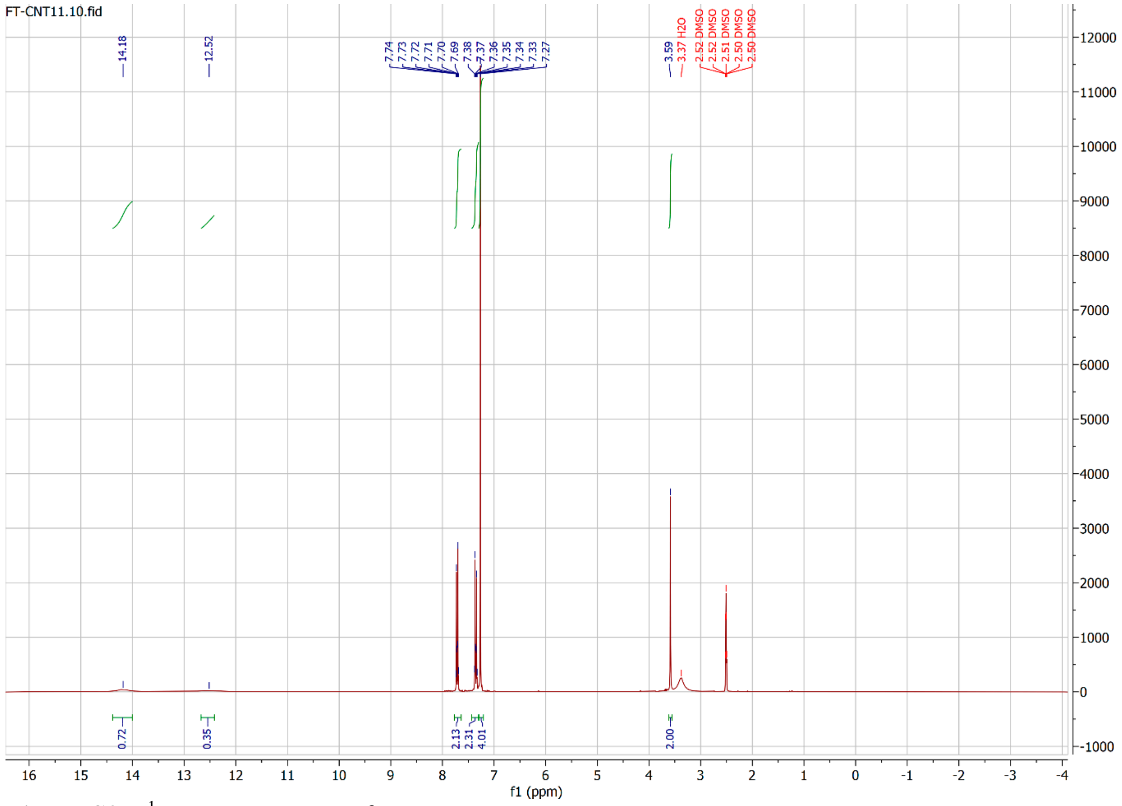

Figure S95. ^{1}H-NMR spectrum for **5d**

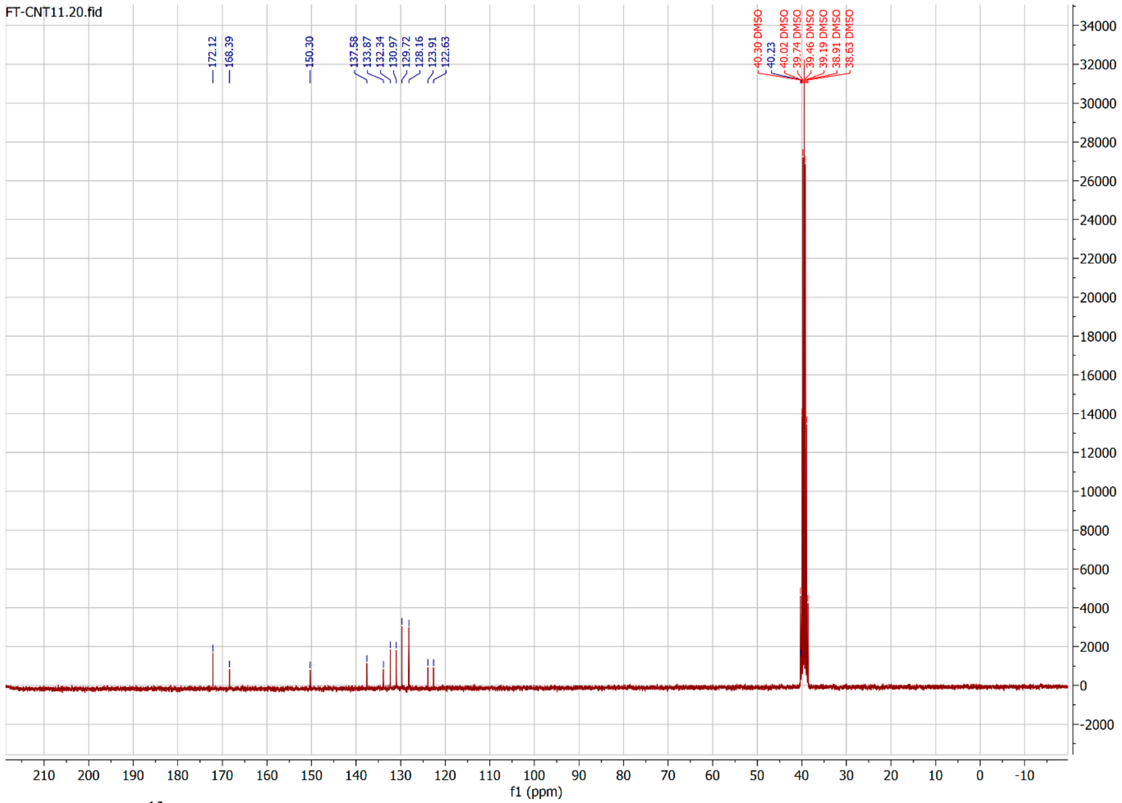

Figure S96. ^{13}C-NMR spectrum for **5d**

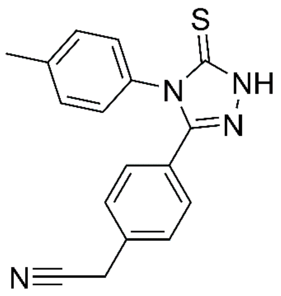

Figure S97. The chemical structure of compound **5e**

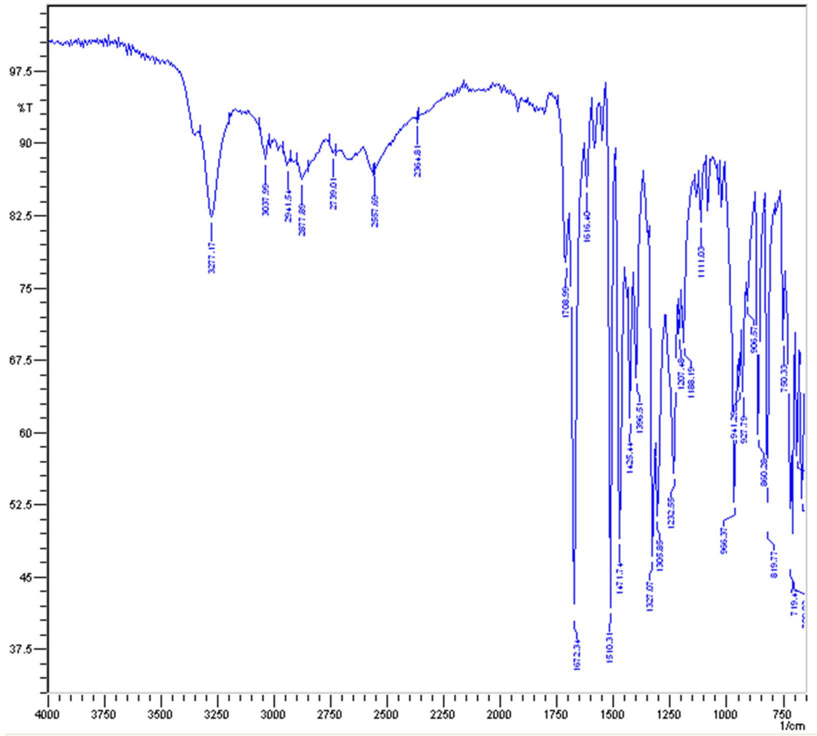

Figure S98. IR spectrum for **5e**

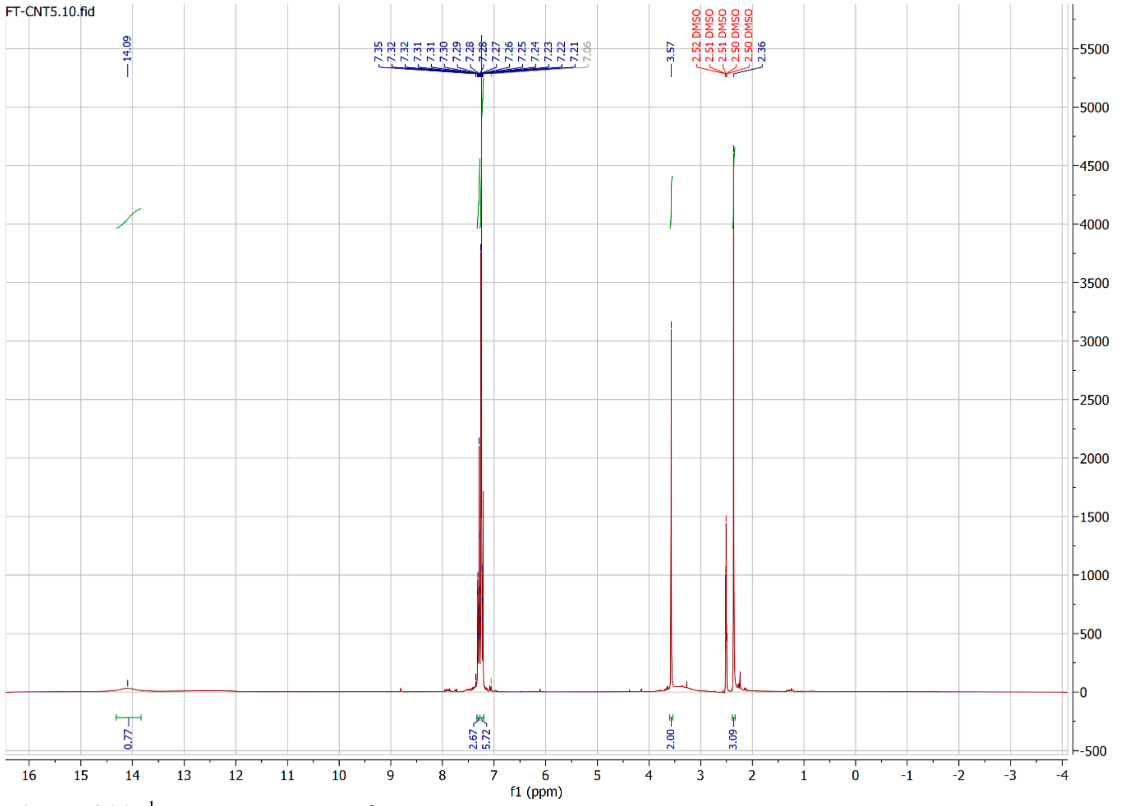

Figure S99. ^{1}H-NMR spectrum for **5e**

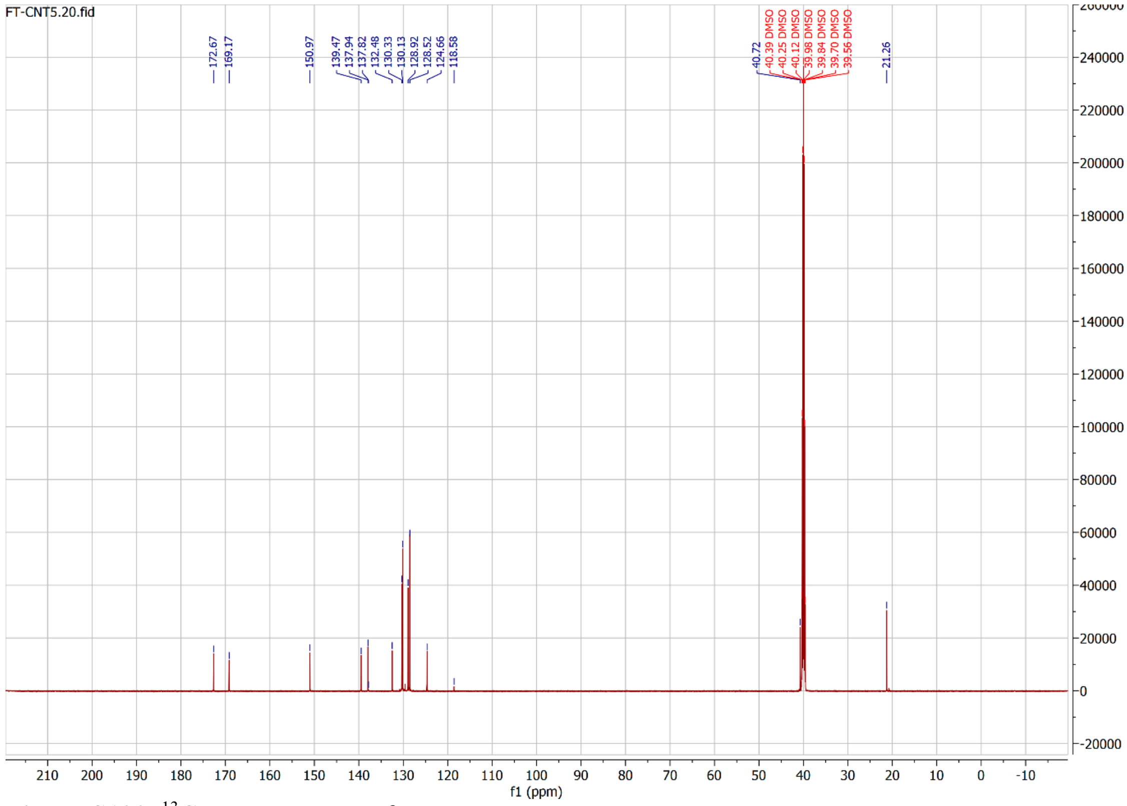

Figure S100. ^{13}C-NMR spectrum for **5e**

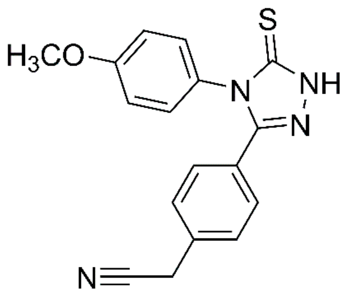

Figure S101. The chemical structure of compound **5f**

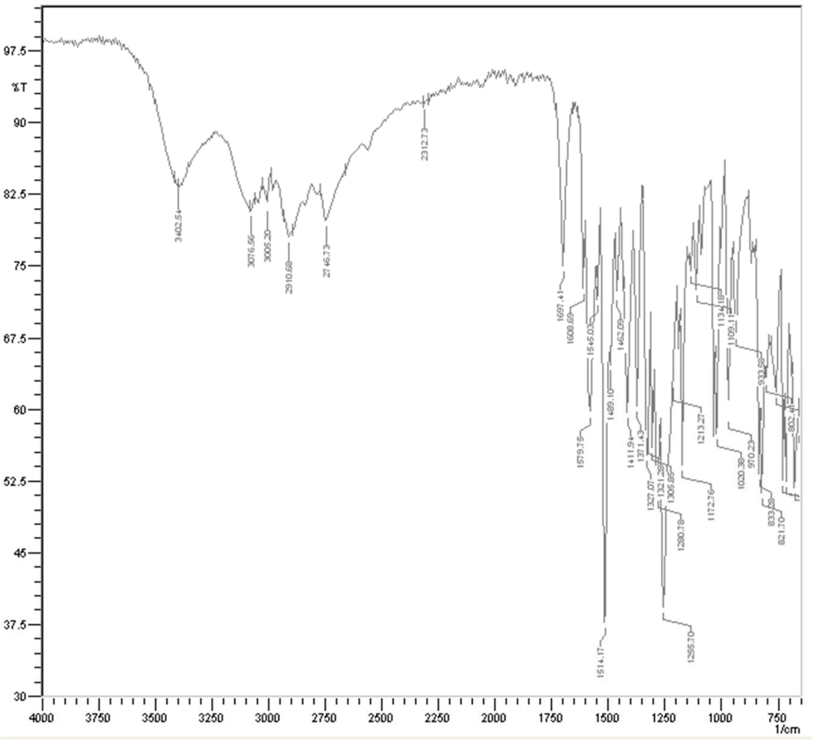

Figure S102. IR spectrum for **5f**

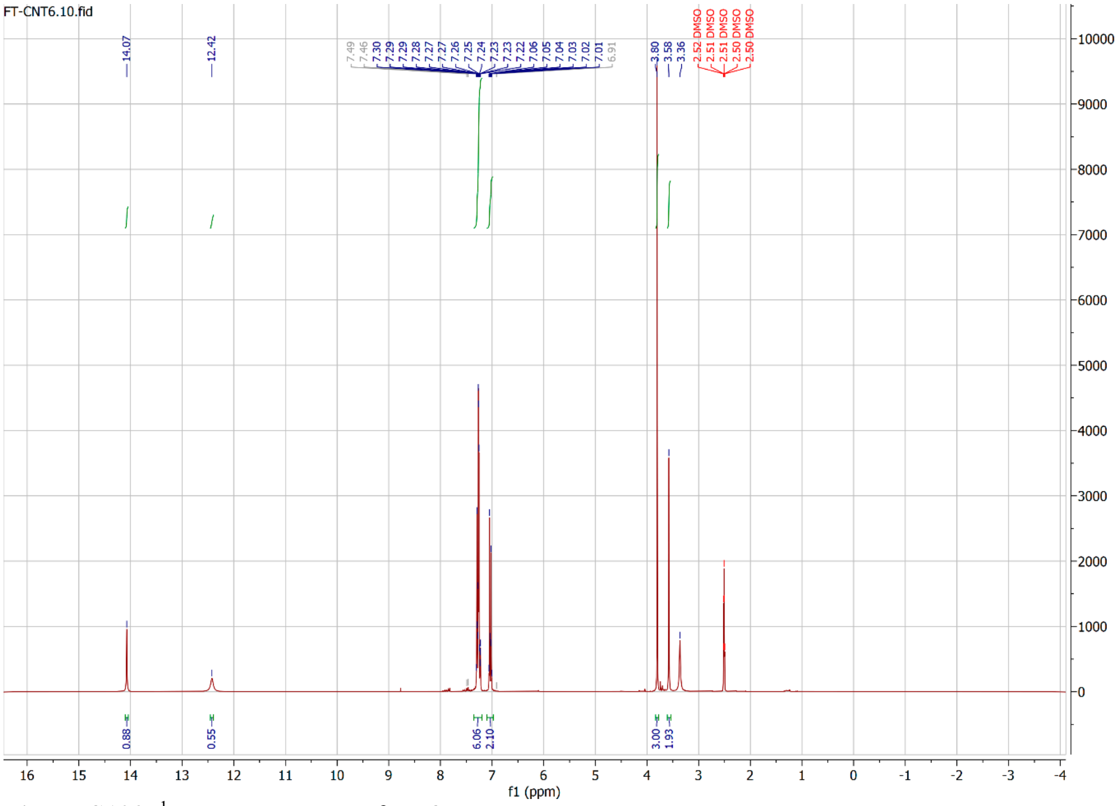

Figure S103. ^{1}H-NMR spectrum for **5f**

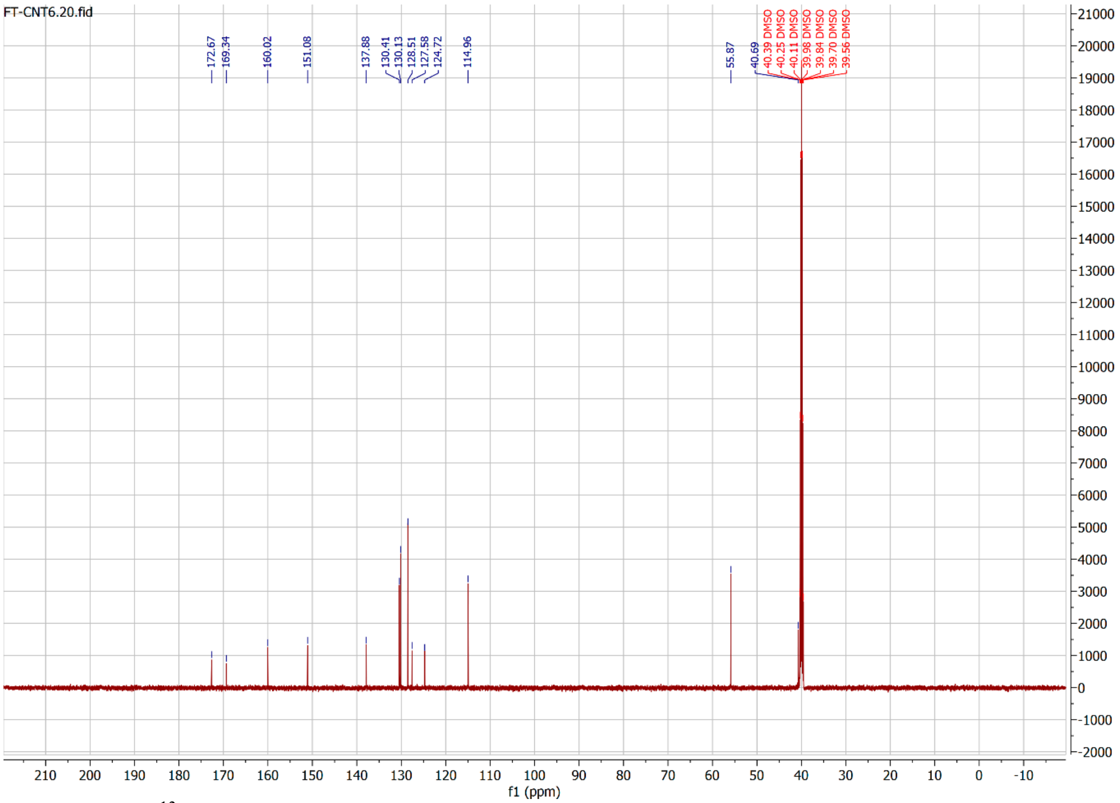

Figure S104. ^{13}C-NMR spectrum for **5f**

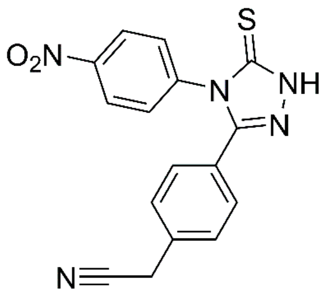

Figure S105. The chemical structure of compound **5g**

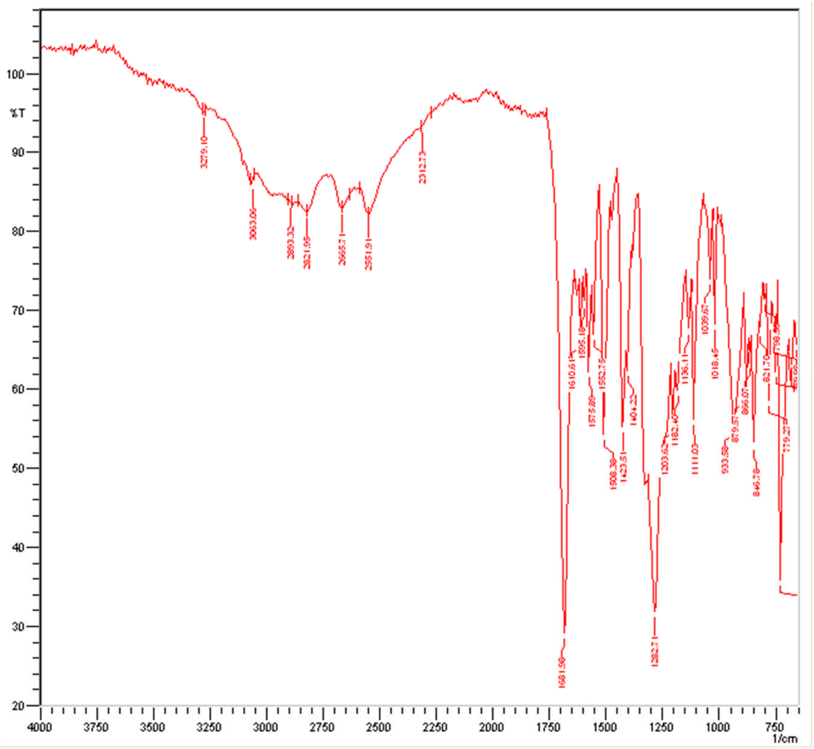

Figure S106. IR spectrum for **5g**

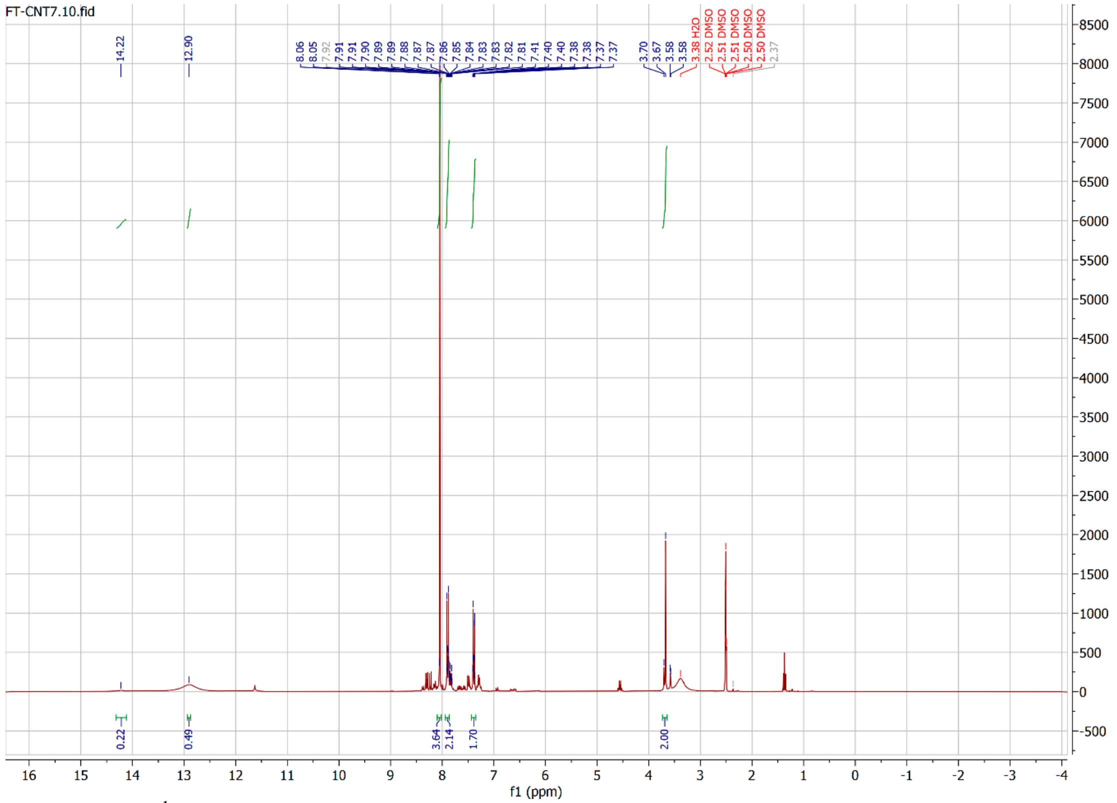

Figure S107. ^{1}H-NMR spectrum for **5g**

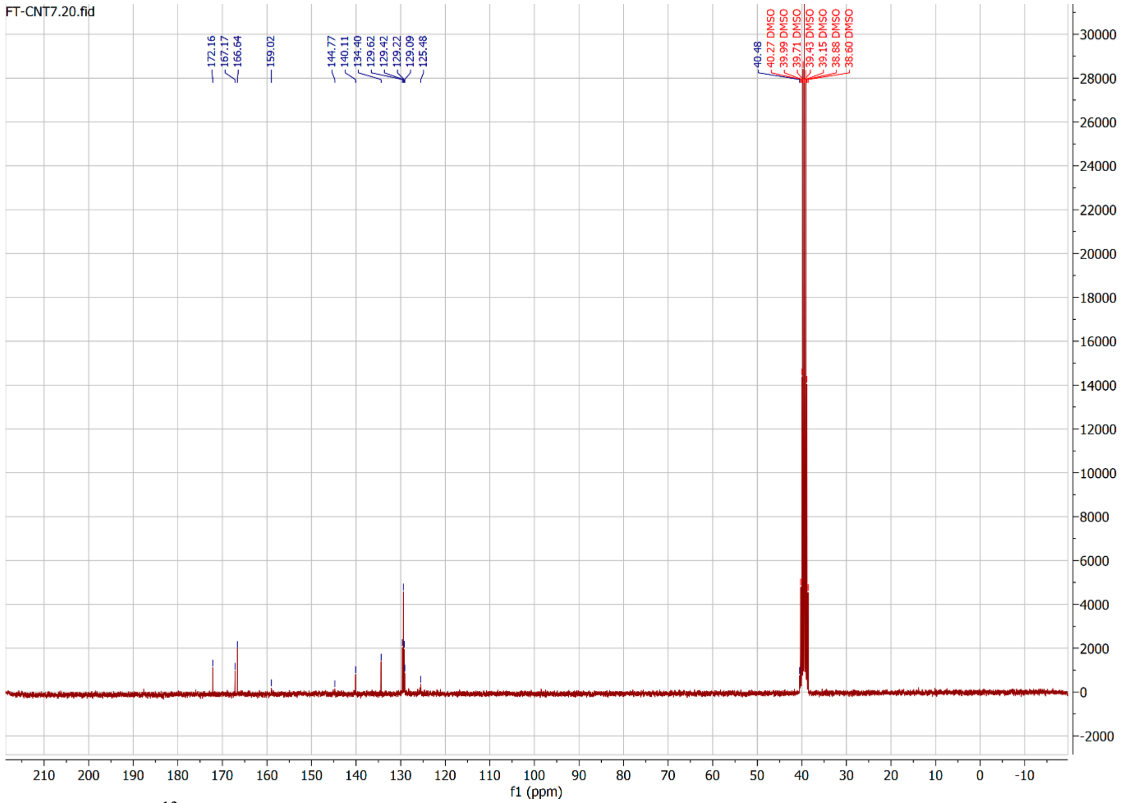

Figure S108. ^{13}C-NMR spectrum for **5g**

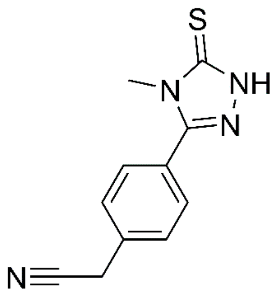

Figure S109. The chemical structure of compound **5h**

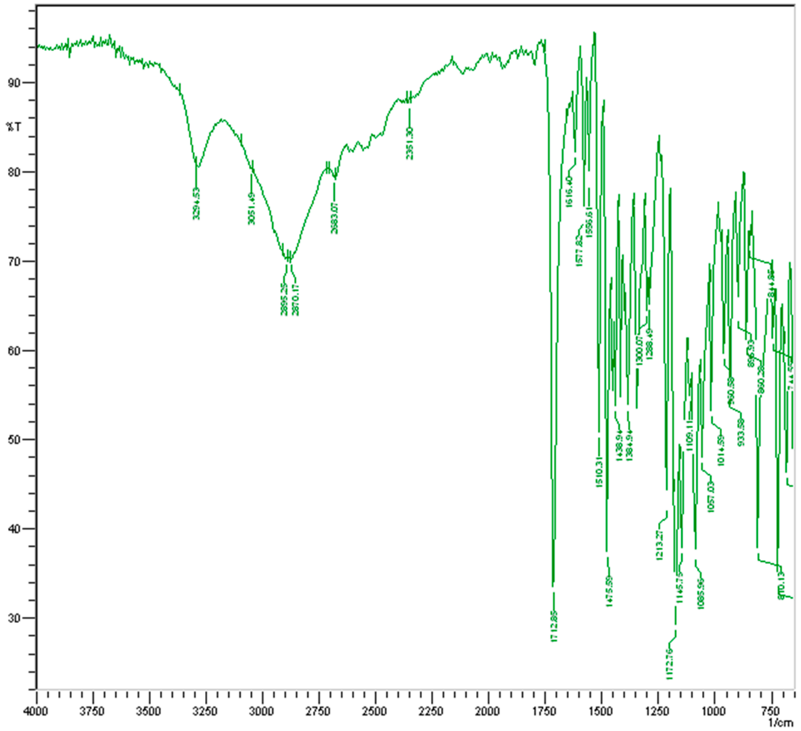

Figure S110. IR spectrum for **5h**

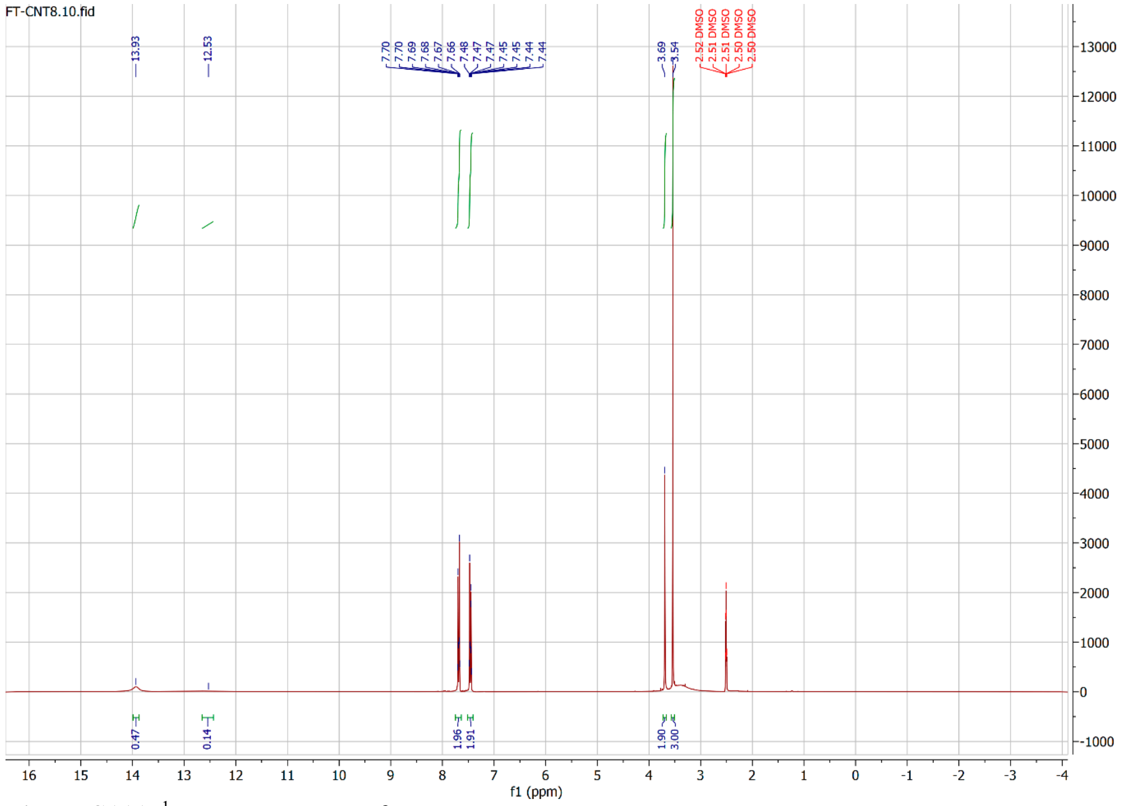

Figure S111. ^{1}H-NMR spectrum for **5h**

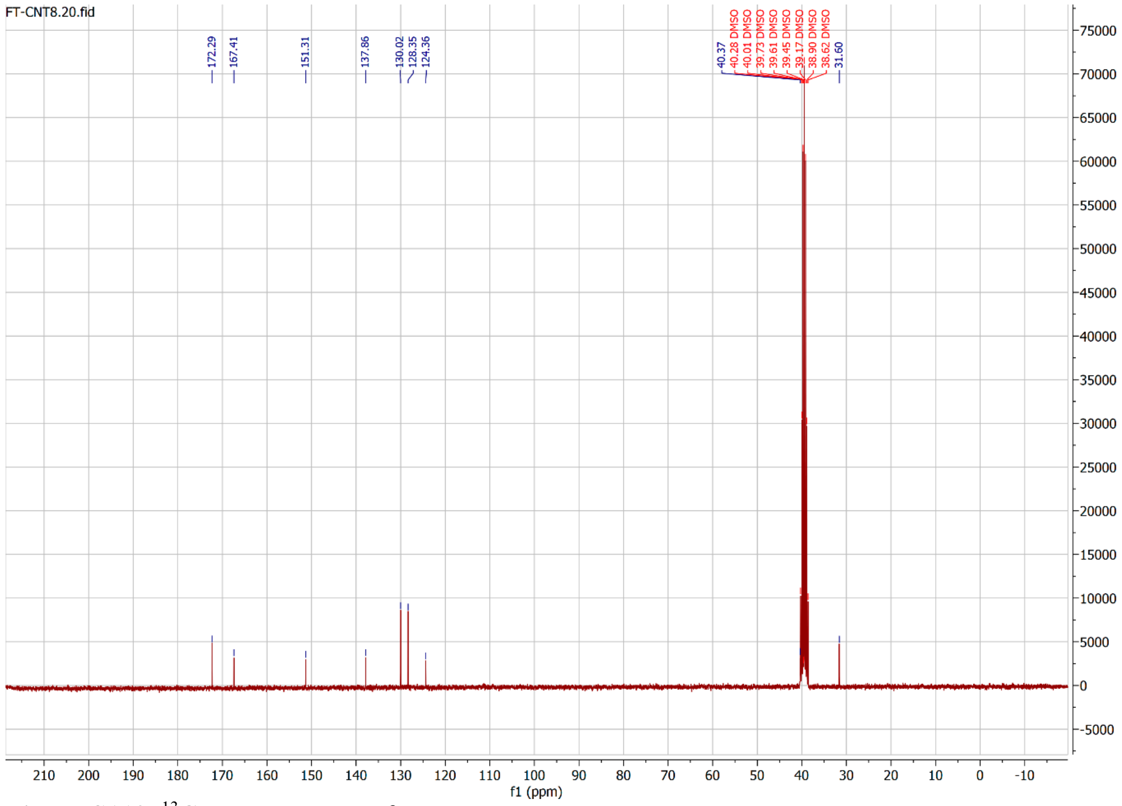

Figure S112. ^{13}C-NMR spectrum for **5h**

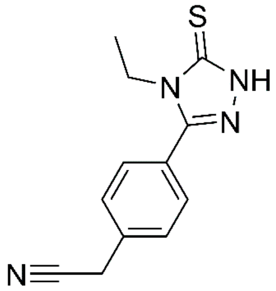

Figure S113. The chemical structure of compound **5i**

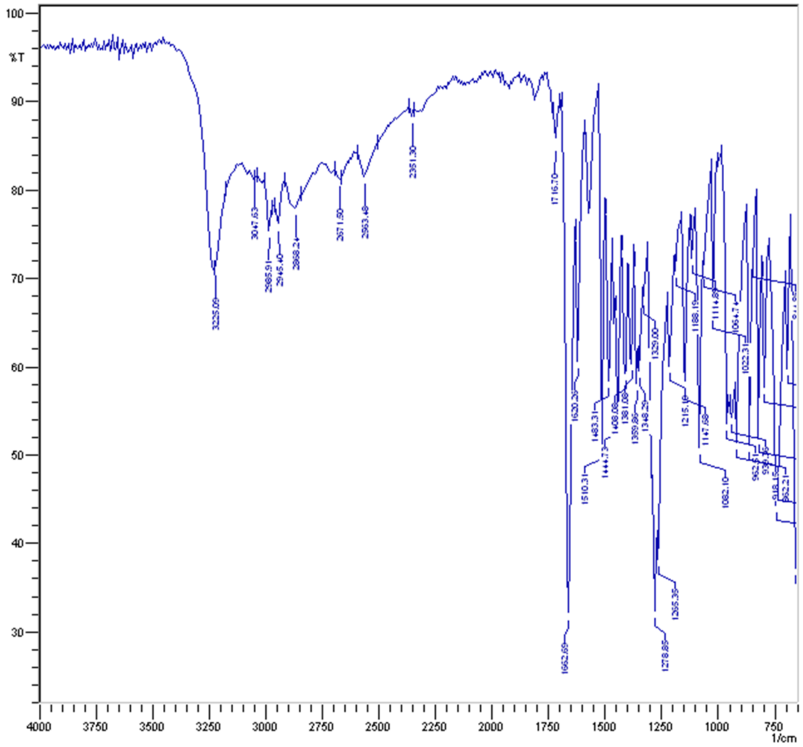

Figure S114. IR spectrum for **5i**

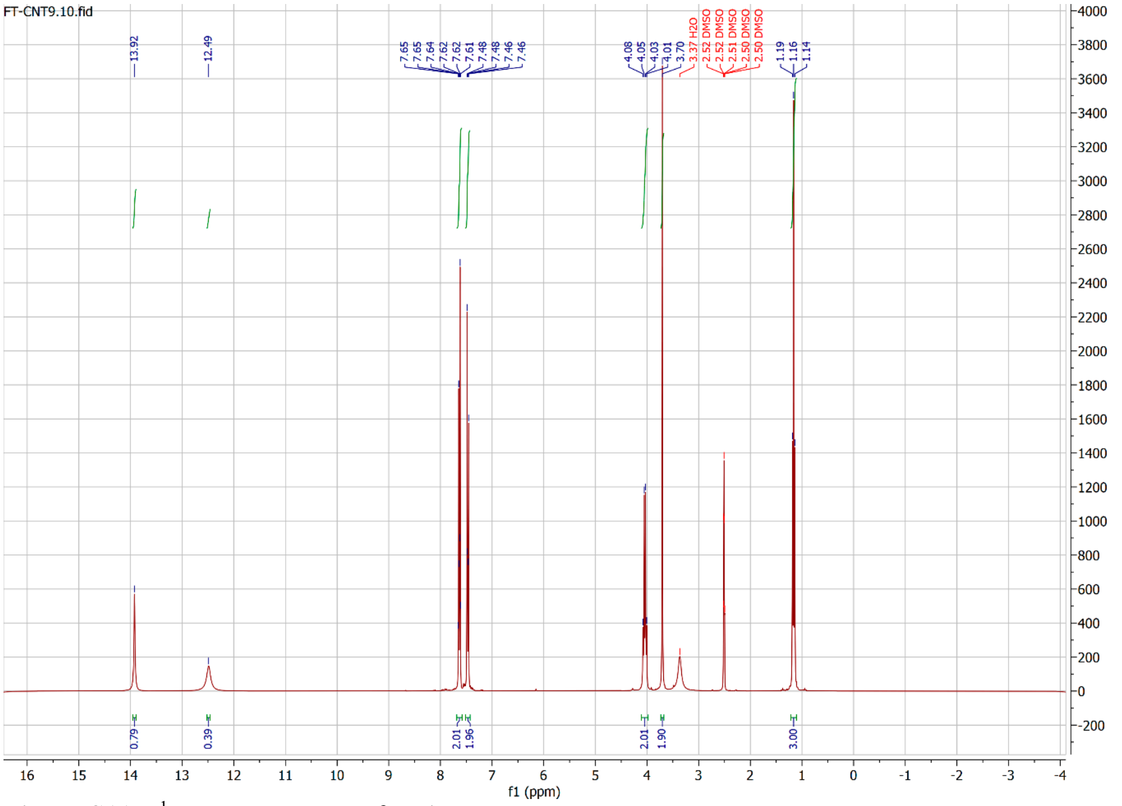

Figure S115. ^{1}H-NMR spectrum for **5i**

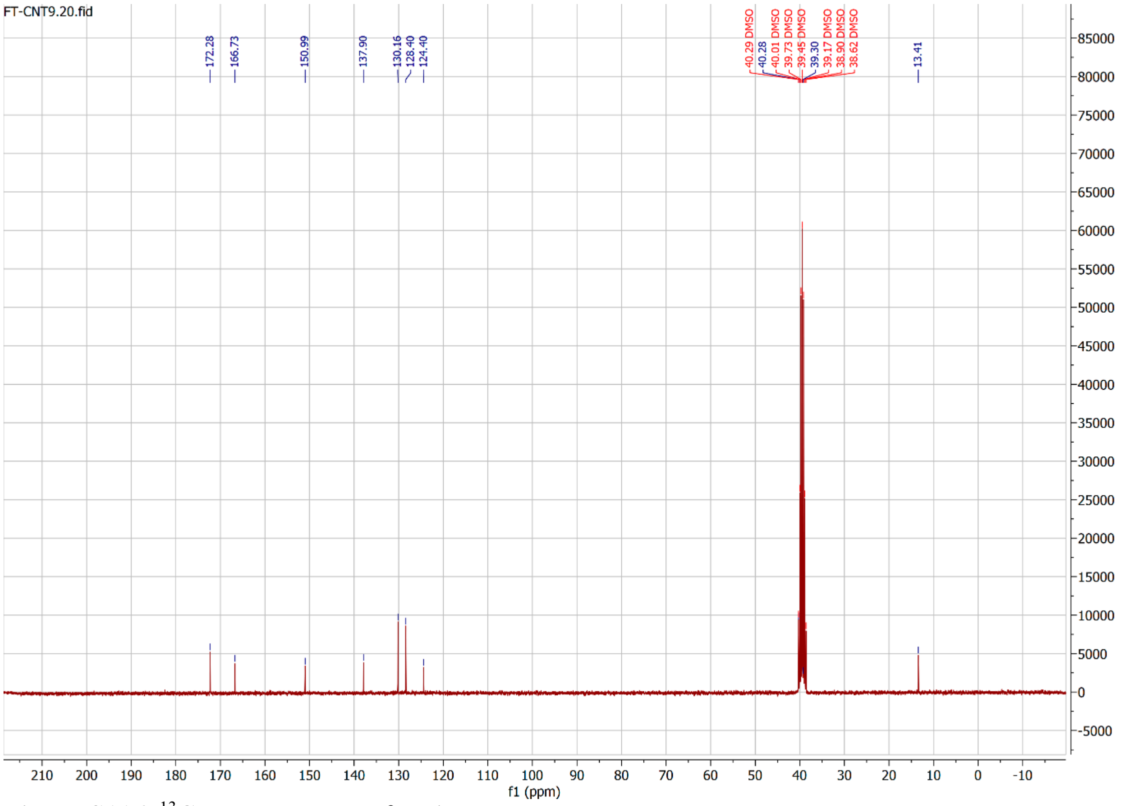

Figure S116. ^{13}C-NMR spectrum for **5i**

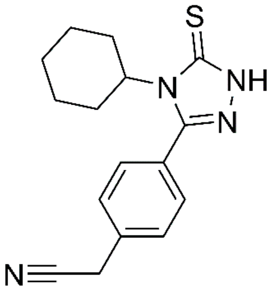

Figure S117. The chemical structure of compound **5j**

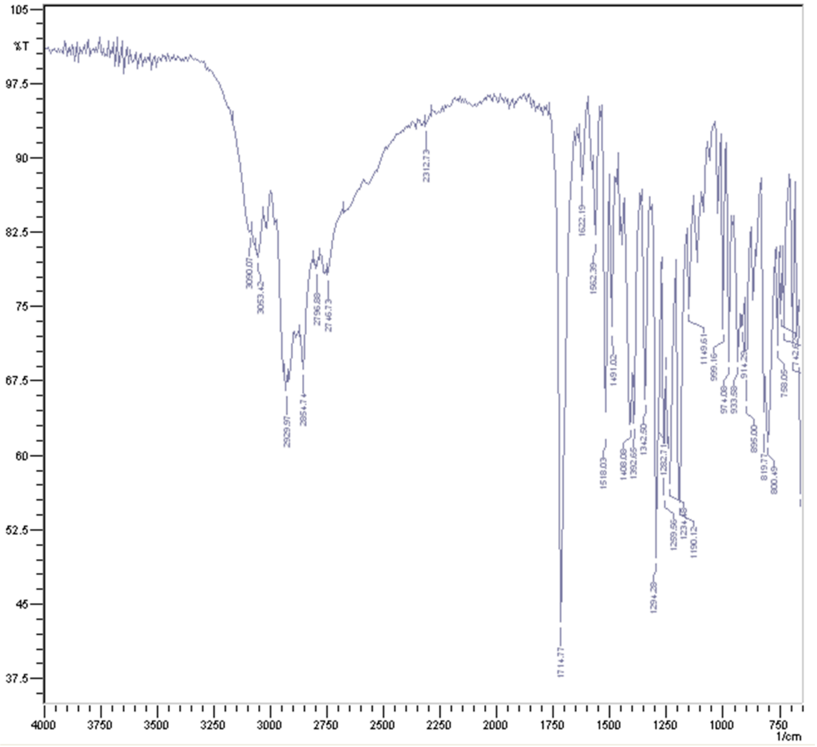

Figure S118. IR spectrum for **5j**

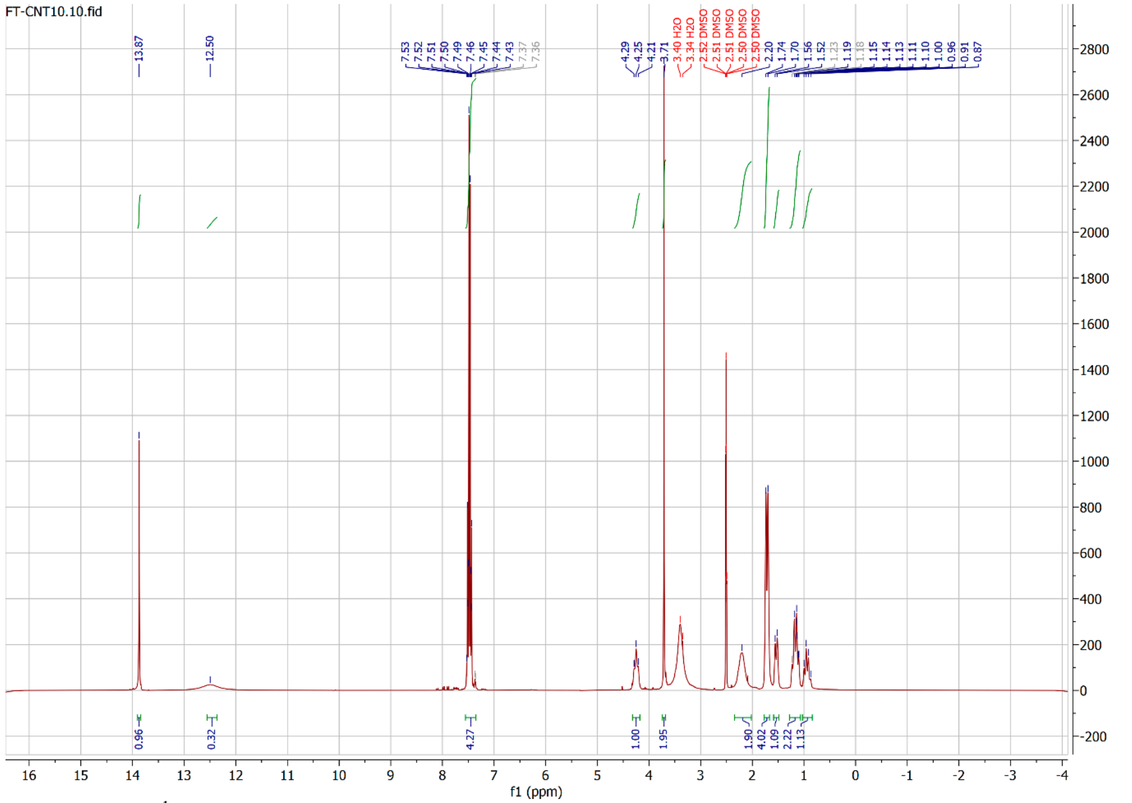

Figure S119. ^{1}H-NMR spectrum for **5j**

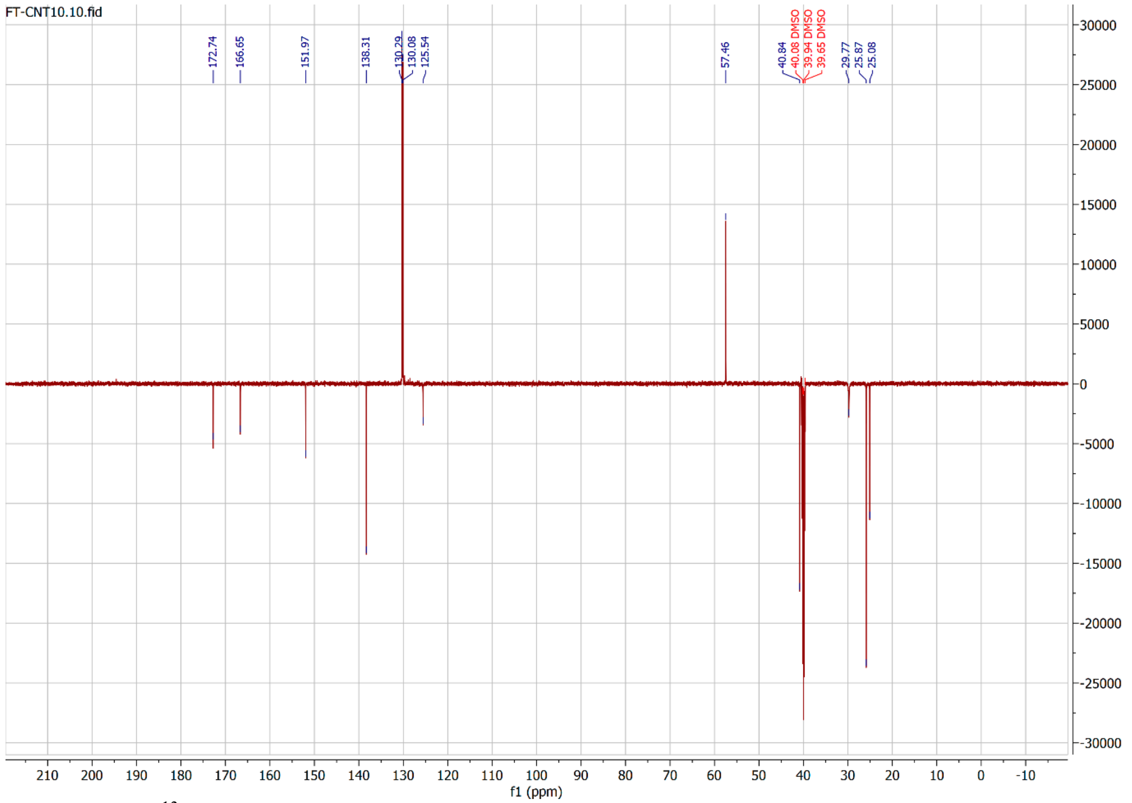

Figure S120. ^{13}C-NMR spectrum for **5j**

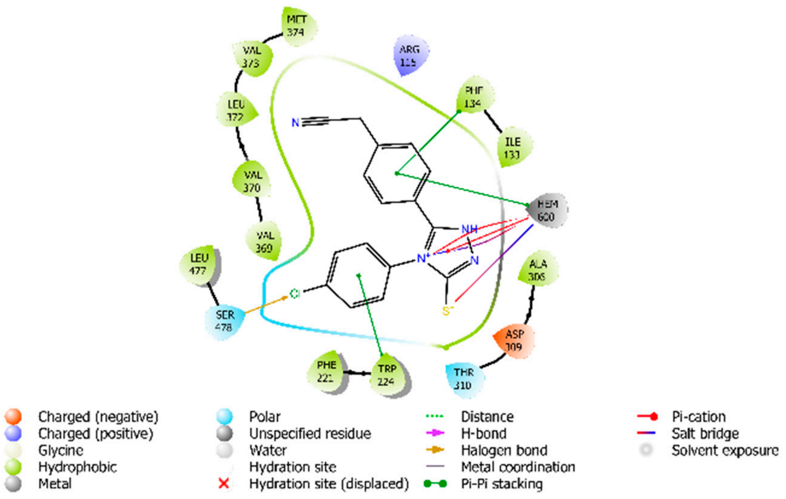
MET 374
VAL 373
ARG 115
PHE 134
LEU 372
ILE 133
VAL 370
HEM 600
VAL 369
LEU 477
ALA 306
SER 478
ASP 309
PHE 221
TRP 224
THR 310
Charged (negative)
Charged (positive)
Glycine
Hydrophobic
Metal
Polar
Unspecified residue
Water
Hydration site
Hydration site (displaced)
Distance
H-bond
Halogen bond
Metal coordination
Pi-Pi stacking
Pi-cation
Salt bridge
Solvent exposure

A

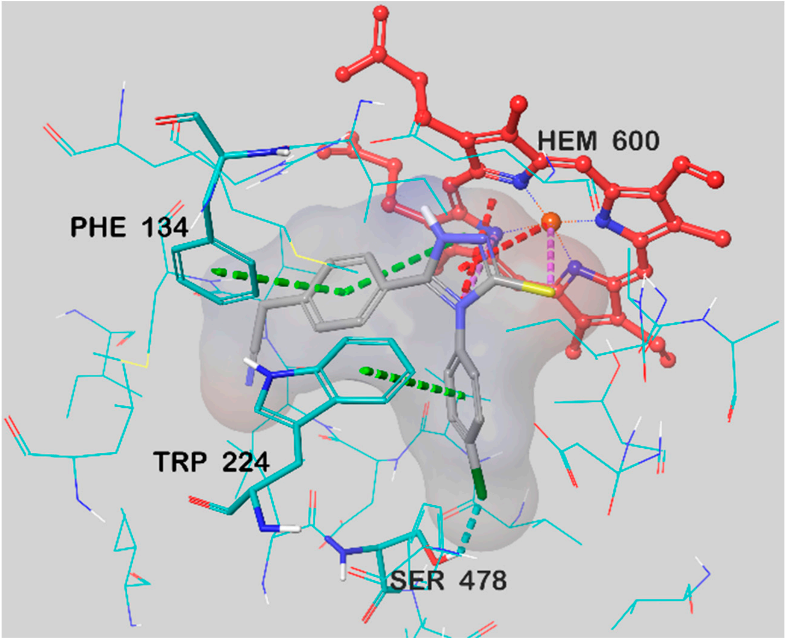
HEM 600
PHE 134
TRP 224
SER 478

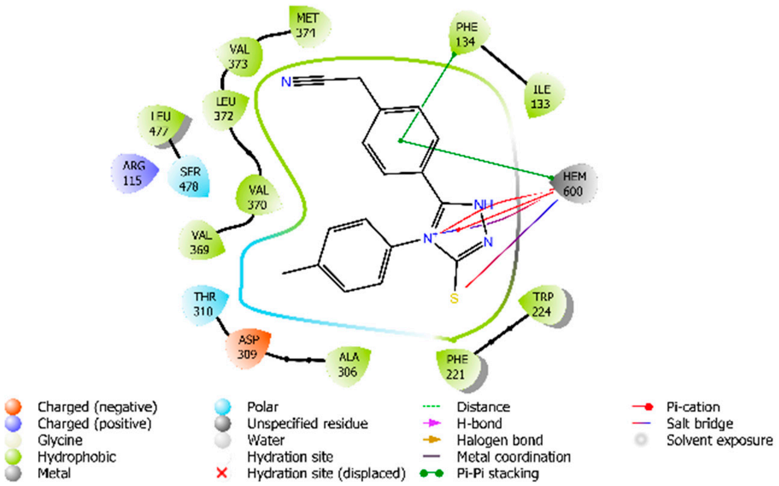
MET 374
VAL 373
PHE 134
LEU 372
ILE 133
LEU 477
ARG 115
SER 478
VAL 370
HEM 600
VAL 369
THR 310
TRP 224
ASP 309
ALA 306
PHE 221
Charged (negative)
Charged (positive)
Glycine
Hydrophobic
Metal
Polar
Unspecified residue
Water
Hydration site
Hydration site (displaced)
Distance
H-bond
Halogen bond
Metal coordination
Pi-Pi stacking
Pi-cation
Salt bridge
Solvent exposure

B

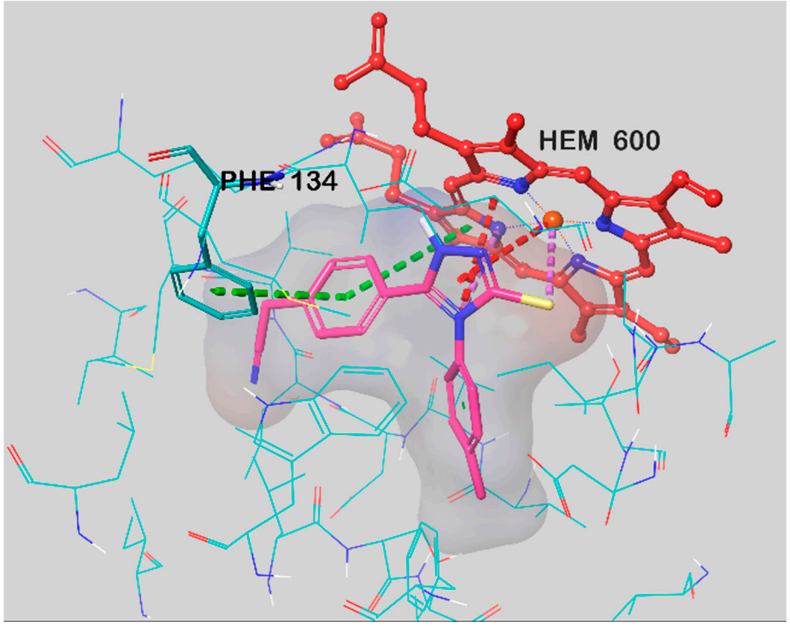
HEM 600
PHE 134

C

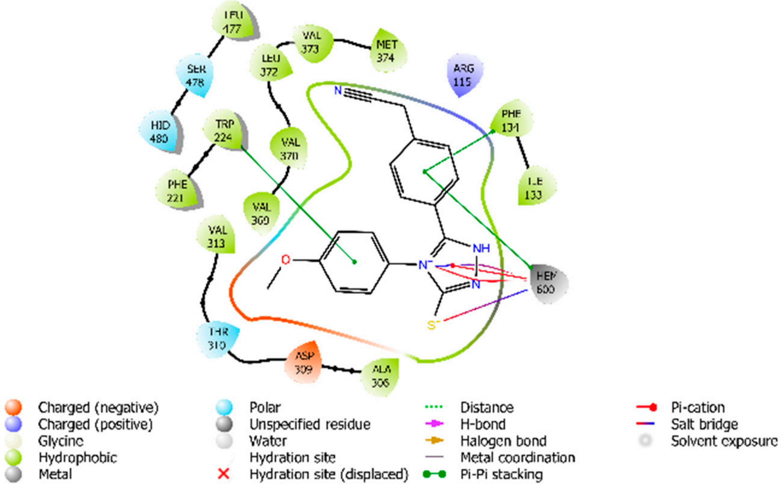
LEU 477
VAL 373
MET 374
SER 478
LEU 372
ARG 115
HID 480
TRP 224
VAL 370
PHE 134
PHE 221
VAL 369
ILE 133
VAL 313
HEM 600
THR 310
ASP 309
ALA 306
Charged (negative)
Charged (positive)
Glycine
Hydrophobic
Metal
Polar
Unspecified residue
Water
Hydration site
Hydration site (displaced)
Distance
H-bond
Halogen bond
Metal coordination
Pi-Pi stacking
Pi-cation
Salt bridge
Solvent exposure

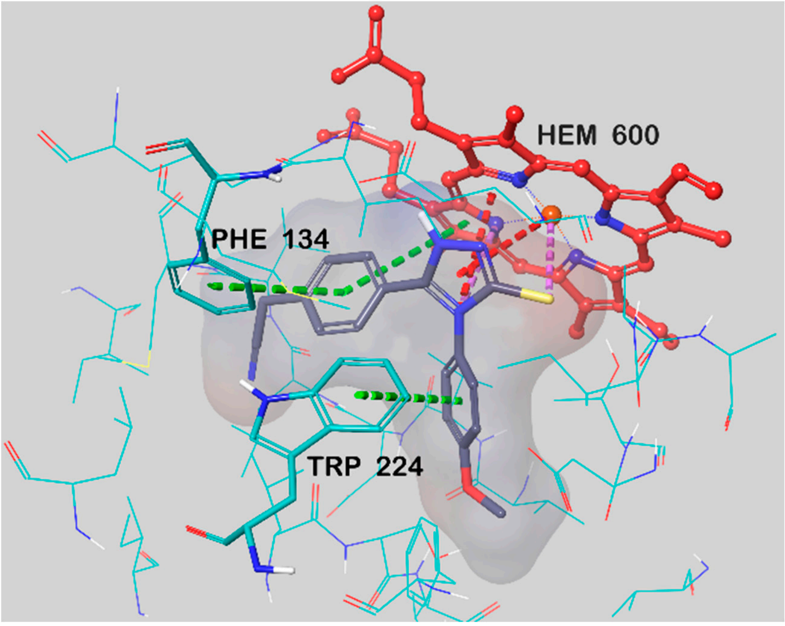

Figure S121. (**A**) Overlay of the binding poses of compounds **5c**, **5e** and **5f** with the aromatase enzyme. Two-dimensional and three-dimensional interaction profiles of derivatives **5c** (**A**), **5e** (**B**), and **5f** (**C**) with the active site of aromatase (PDB ID: 3EQM)
